# Impact of safe-anastomosis digital training on 30-day anastomotic leak after right colectomy: international prospective cohort study (EAGLE-2)

**DOI:** 10.1093/bjs/znag107

**Published:** 2026-09-11

**Authors:** ESCP EAGLE Safe Anastomosis Collaborative and NIHR Global Surgery Unit, Elizabeth Li, Sue Blackwell, Pamela Buchwald, Laura Castro, Sanjay Chaudhri, Sharfuddin Chowdhury, Dragomir Dardanov, Audrius Dulskas, Muhammed Elhadi, Alaa El-Hussuna, Caterina Foppa, Matteo Frasson, Gaetano Gallo, James Glasbey, James Keatley, Michael Kelly, Beatriz Silva Mendes, Ana Minaya, Peter Neary, Ionut Negoi, Francesco Pata, Thomas Pinkney, Gabrielle van Ramshorst, Shaji Sebastian, Baljit Singh, Dion Morton, Omar Omar, Bryar Kadir, Elizabeth Li, Dion Morton, James Keatley, Elizabeth Li, Sivesh Kamarajah, Eleanor Oliveira, Dion Morton, Simon Ng, Nicolas Avellaneda, Erman Aytac, Romina Bianchi, Pamela Buchwald, Peter Christensen, James Keatley, Suk-Hwan Lee, Varut Lohsiriwat, Surendra Kumar Mantoo, Klaus Matzel, Luqman Mazlan, Frederic Ris, Jun Won Um, Carolynne Vaizey, Jaw Yuan Wang, Janindra Warusavitarne, Jun Watanabe, Sofia Xenaki, Hongwei Yao, Nicolas Avelleneda, Maria Bubenova, Helio Moreira Junior, Martin Karamanliev, Peter Vlcek, Mark Bremholm Ellebæk, Baljit Singh, Mahmoud M Elsayed, George Tzovaras, Amrit Pipara, Marco Caricato, Gaetano Gallo, Francesco Pata, Paulius Žeromskas, April Roslani, Muhammed Elhadi, Johanne Bloemen, Miguel Cunha, Christina Fleming, Peter Neary, Ionut Negoi, Mohammed Sbaih, Ana Minaya Bravo, Jeremy Meyer, Pamela Buchwald, Ibrahim Mutwakil, Arda Isak, Sezai Leventoglu, Andriy Beznosenko, Farid Allam, Farouk Bakhti, Ghania Rahmoune, Dounia Aktouf, Hamid Aliyahia, Agustina Zabani, Oscar Silva, Patricia Orsingher, Candelaria Beruti, Carina Chwat, Diego Valli, Flavia Alexandre, Gustavo Lemme, Lucas Peirano, Mauro Ramírez Duarte, Antonela Di Gennaro, Claudia Carrizo, Fabio Leiro, Julieta Espino, Mauro Trama, Federico Yazyi, Magali Muthular, Alexis Braggio, Diana Alejandra, Vasquez Velasquez, Leandro Stawkowy, Oriana Comodo, Dario Barrera, Nicolás Luis Avellaneda, Sonia Antonela La Rosa, Ariel Enrique Grinblat, Rocio Ailen Gau, Augusto Javier Carrie, Chun Hin Angus Lee, Jason Cox, Anna Fiess, John Edington, Jasmine Tan, Jessica Paytner, Melissa Phu, David Proud, Lyons Nick, Amanda Kerton, Ranjan Guha, Ian Ong, Thomas Tiang, Dominik Weihs, Kristina Wrodnigg, Claudia Wette, Dirk Bernard, Daniel Schmid, Gergely Kovacs, Maria Bubenova, Bernhard Beer, Karin Kreuzhuber, Jürgen Simharl, Stefan Stättner, Gerd Pressl, Thomas Saini, Verena Wieser, Matthias Biebl, Clemens Rohregger, Wolfgang Zaglmair, Felix Aigner, Zoltan Horvath, Markus Braumille, Geza Gemes, Nicole Koter, Gabriele Moitzi, Elisabeth Wallner, Iliyan Iliev, Michaela Layr, Ines Stipic, Bernhard Dauser, Andrey Litvin, Valentin Bereshchenko, Valentin Dashkevich, Aleksander Misevich, João Fraga, Jeud Bravo, Marylin Da Silva, Zamir Fernando, Barbara Prais, Marcela Paula, Fábio Jabour, Fabio Lopes de Queiroz, Daniel Mauricio, Londoño Estrada, Alexandre Sette, Bruno lucena de souza Pires, Marco Antonio Miranda Dos Santos, Helida Aline Tomacheski Amaral, Antonio Lacerda Filho Felicio Rocho, Carlos Augusto Real Martinez, Danilo Toshio Kanno, Patricia Krause Askinis, Matheus Gobbo Cordeiro, Fernando Augusto Lima Marson, Camila Vantini Capasso Palamim, Roberto Kaiser Junior, Mikaell Faria, Ana Regina Bptista, Anderson Gomes, Manol Sokolov, Angel Arabadzhiev, Dimitrin Tonova, Gergana Ivanova, Svilen Maslyankov, Tsvetan Popov, Dobromir Dimitrov, Martin Karamanliev, Galya Pacheva, Slavina Yohanova, Meri Shoshkova, Paulina Vladova, Preslav Vasilev, Xiaodong Wang, Jianbo Liu, Yue Wen, Fei Liu, Zdeněk Kala, Martin Svoboda, Lucie Fraňková, Deana Slovjaková, Vladimír Procházka, Tomáš Grolich, Petr Vlček, Beata Hemmelová, Denisa Kliková, Miloš Chobola, Tamara Vystrčilová, Alena Berková, Michal Reška, Zuzana Šerclová, Aneta Tremerová, Martina Rumlová, Iveta Čechová, Petra Zusková, Jakub Freml, Jaroslav Kalvach, Kristýna Pončáková, Michal Ondica, Jan Votava, Matúš Murín, Mark Bremholm Ellebaek, Jens Kristian Baelum, Dorthe Kathrine Slot, Søren Rune Larsen, Lars Uldum Erlandsen Claville, Pedja Cuk, Rasmus Tofte Hansen, Aleksandar Dzodan, Michael Tvilling Madsen, Kathrine Nagel Host, Abdullah Mostafa Al-Ansari, Miguel Cordero Muñoz, Alexander Rodriguez, Sheila Palma, Gabriela Torres, Galal Abouelnagah, Mohamed Al Sayed, Engy Amgad, Ahmed Ibrahim, Hashem Altabbaa, Nour Eldein Saad, Albaraa Daradkeh, Mohamed Zarad, Enas Mohamed, Ghaida Hamid, Guma Khalid, Abd Elrahim Mohamadin, Mohamed Magdy Shaapan, Marwa Adel Arafa Ebrahim, Amr Abd Ellatif Mostafa, Abdelrahman Elmorshdy, Alaa Ibrahim, Sara Elmorshdy, Hossam El-Fol, Mohamed Bassiony, Khalid Mohamed, Mostafa Mansour, Mohamed Elkhouly, Salma Kemaz, Hatem Amr Elzahaby, Mohamed Hefzy, Marwan Abdou, Ahmed Khedr, Ahmed Moussa, Aya Bessa, Mohamed Badawy, Mohammed Elalamy, Mohamed Abdalsalam, Ibrahim Beshai, Aya Nassar, Mahmoud Abdelmodaber, Omnia Radwan, Ahmed Gomaa, Salma Abdou, Mahmoud M Elsayed, Ahmed Khalifa, Ahmed M Elsayed, Nermen Farag, Mohamed Sorour, Ahmed Nagah, Omar Hafez, AhmedSaber Mohamed Abdelrahman, Sarah Mansour, Kariman Saber, Mostafa Farag, Ahmed Hafez, Alaaeldin Esmail, Marwan Fakhry, Said Rashed, Yara Hani, Shahd Ramadan, Hasnaa Elshazly, Hesham A Elmeligy, Omar Younes, Samar Mohammed, Hend F Hassan, Hazem Korani, Islam Metwally, Mohamed Elbanna, Khaled Abdelwahab, Mosab Shetiwy, Mohamed Abdelkhalek, Saleh Elbalka, Sarah Magdy Abdelmohsen, Mohie El-Din Madany, Jamal Hussein, Tarek Hemaida, Aya Ziada, Mahmoud M Elsayed, Bushra Qabala, Hussein Hussein, Khalid Sarhan, Mohamed Sherif Ali Ahmed, Rashad G Mohamed, Ibrahim Saleh Alawadi, Mohamed W Omar, Ahmed Hewadi, Sherif Wael, Youstina Mohsen, Mohammed Yasser Abdelfattah, Abdulrahman Hesham Khairy, Ayman Taher Shemes, Hisham Hazem Warda, Mostafa Ismail Mahmoud Hassan, Mohamed Salama, Mohamed Attia Elfadali, Yara Sherif Elsayed, Mohamed Sherif Ali, Ahmed Shehta, Amr Sanad, Aziza El-Saey, Moataz Emara, Mohamed Sherif, Yara Sherif, Mostafa Ismail, Mohamed Naieem Abdelaal, Mohamed Talaat Abdelhalim, Mahmoud Rabie, Karim Khattab, Ahmed Abo Saif, Mohamed Nazeef, Heba Taher, Mujtaba Zakria, Walaa Mohamed Kamel, Sara Saleh, Moamen Hassan, Abobakr Saleh, Noureldin M Sayed, Ahmed Y Azzam, Osman Elamin, Omar S Elsayed, Mahmoud M Morsy, Adam Elswedy, Amr Kassem, Ahmed Ibrahim, Ahmed Ahmed, Ahmed Ebrahim, Emad Mostafa, Omar Zayed, Abdrahman Ramadan, Manar Zein, Youssef Eid, Karim Elhusseini, Mohamed Hamed, Ahmed Abostate, Ahmed Reda, Eslam Radwan, Ahmed Mohsen, Omar Saeed, Abdelrahman Saeed, Mahmoud Ramadan, Fitsum Terefe, Benjamin Muessle, Jan-Philipp Ruff, Annett Rauscher, Stephan Möller, Nuh Rahbari, Marko Kornmann, Emrullah Birgin, Ulf Kulik, Alexander Wagner, Winny Andrea, Hendrik Eismann, Christel Koczur, Regine Pfeiffer, Steffen Seyfried, Maike Hermann, Lena Lamm, Valentin Walter, Julia Hardt, Christoph Reißfelder, Frieder Pullig, Robby Langbecker, Emmanuel Kafui Ayodeji, Spiros Delis, Evgenia Charitaki, Alexia Nduska, Nikos Pentilas, Dimitrios Karakaxas, George Theodoropoulos, Maximos Frountzas, Petros Georrgitsogiannakos, Eleni Pagalou, Vasileios Kostoulas, Despoina Kanata, Konstantinos Stamou, Sotirios Sterpis, Eleni Kerasioti, Georgios Retzios, Vasileios Papadopoulos, Paraskevi Chatzikomnitsa, Savvas Mavromatidis, Dimitris Bliamplias, Dimitrios Giakoustidis, Alexandros Giakoustidis, Gregory Christodoulidis, Francesk Mulita, Levan Tchabshvili, Eleni Bekou, Admir Mulita, Elias Liolis, George Tzovaras, Ioannis Baloyiannis, Chamaidi Sarakatsianou, Eleni Arnaoutoglou, Konstantinos Perivoliotis, Ioannis Mamaloudis, Effrosyni Bompou, Elissaios Kontis, Ioannis Katsaros, Athina Oikonomou, Christos Maniatis, Charalampos Theocharopoulos, Athanasios Syllaios, Zoi Gkiafi, Irena Myrtaj, Asimina Valsamaki, Antonios Koutras, Stavros P Papadakos, Alexandros Pergaris, Thalia Petropoulou, Artemis Liapi, Lounta Bouznic, Aris Bakas, Dimitrios Schizas, Nikolaos Machairas, Adam Mylonakis, Panagiota Stratigopoulou, Stylianos Kykalos, Myrto D Keramida, Panagiotis Sakarellos, Ioannis Papaconstantinou, Leonidas Chardalias, Eirini Kalamara, Kassiani Theodoraki, Theodosios Theodosopoulos, Manousos Konstadoulakis, Dimitrios Politis, Manousos Christodoulakis, Andreas Strehle, Sofia Papantonaki, Orestis Ioannidis, Elissavet Anestiadou, Despoina Fragkiadaki, Ioanna Dimitropoulou, Konstantinos Siozos, Ourania Kerasidou, Aliki Brenta, Kaori Futaba, Nicole Miu Yee Cheng, Wing Kin Tse, Vivian Nga Man Lau, Balazs Banky, Tímea Drexler, Ágnes Kis, János Széll, Mátyás Hamar, András Fülöp, Ankur Garg, Rana M Rajneesh, Pankaj Kumar, Sandeep Kumar, Satish Subbiah Nagaraj, Naveen Pentakota, Arun Chandran, Karan Singla, Cherring Tandup, Yashwant Raj Sakaraj, Lileswar Kaman, Amit Gupta, Ammapalem Satish, Umesh Kumar Sedwal, Praveen Talawar, Deepak Rajput, Rahul Kumar, Ritu Thakur, Poorvikha S, Vinutha Gowda, Chandrakanta Gowda, Savitri Maridevegowda, Tushar Subhadarshan Mishra, Soumyajit Jana, Babu Lal Jakhar, Neha Singh, Dillip Kumar Muduly, Bramhadatta Pattnaik, Monika Gureh, Pradeep Saxena, Siddharth Kumar Sinha, Morpal Meena, Vaishali Waindeskar, Ajeet Pratap Maurya, Diwakar Pandey, Tushar N Suryawanshi, Rahul Singh, Hiten Mistry, Nivesh Agarwal, Subham Jakhar, Deepak Kumar, Gunjan Chaudhary, Mohinder Kumar Garg, Utkarsh Kumar, Lovenish Bains, Mohamed Mustaqeem, Sourabh Das, Lalit Gupta, Rahul Gupta, Deepak Gusain, Laxmi Goyal, Rahul Varshney, Ashwani Kumar, Manisha Aggarwall, Parth Dhamija, Lalit Garg, Ashwani Kumar, Amrit Pipara, Moameren, Huirongbam Madon Singh, Neha Desai, Shiv Rajan, Vijay Kumar, Devanshi Verma, Tanmay Tiwari, Naseem Akhtar, Mohit Singh, Abhilasha Tripathi, Sharada Sundaramurthy, Suvam Kumar, Eloni K, Samzai Lungalang, Handilu Rengma, Durgesh Ommi, Bharath S Veerabhadrappa, Badareesh L, Prema Keerthi Shetty, Rama Rani K, Stanley Mathew, Rauf Wani, Fazl Parrey, Gowhar Aziz, Showkat Gurcoo, L V Gouri, Mahadev Prasad Patra, Prativa Das, Arum Kumar Sahoo, Haris Chandra Dash, Jyotiranjan Mohapatra, Rojalin Parida, Santosh Kumar Roy, Chandrashekar Mahakalkar, Shivani Kshirsagar, Arun Teja Karusala, Akaksha Yachmanani, Arwa S Alabide, Ahmed Qasim Mohammed Alhatemi, Esraa S Alabide, Hashim Talib Hashim, Maytham A Al-Juaifari, Najah R Hadi, Mohammad Hussein Abd Munaf, Ghadeer Mohammed Abbas, Eathar Haidar Aljubori, Ahmed Sermed Al Sakini, Sandra Thair Al-Aish, Ali Al-Shammari, Anne Sermed Al Sakini, Aya Saad, Peter Neary, Haroon Ur Rashid, Collette Flynn, Vida Hamilton, Michael Kelly, Maeve O'Neill, Ben Creavin, Brian Mehigan, John Larkin, Paul McCormick, Francesco Autuori, Francesco Madeddu, Marco Nieco, Sabrina Pilloni, Gaetano Luglio, Michele Cricrì, Salvatore d'Angelo, Martina D'Aniello, Francesca Paola Tropeano, Antonio Miele, Giovanni Aprea, Marianna Capuano, Salvatore D'Angelo, Giuseppe De Simone, Giuseppe Palomba, Raffaele Basile, Alberto Aiolfi, Francesco Cammarata, Federica Grasso, Davide Gottardello, Michele Manara, Davide Bona, Giuseppe Sena, Giorgio Ammerata, Marco Scozzafava, Marco Pignataro, Diego Sasia, Ribero Dario, Bonardello Michela, Palmisano Sarah, Lucia Mir Mennea, Chiara Montini, Giacomo Bottero, Laura Fortuna, Francesco Coratti, Fabio Staderini, Gabriele Baldini, Giuseppe Sica, Giulia Bagaglini, Francesco Ferrara, Rosaria Lo Bianco, Mauro Podda, Eleonora Locci, Chiara Curcio, Salvatore Sardo, Adolfo Pisanu, Valentina Murzi, Paola Marongiu, Nicoletta Sveva Pipitone Federico, Roberta Tutino, Michela Forgione, Gelsomina di Ciero, Mauro Santarelli, Francesca Sbuelz, Bruno Scotto, Francesca Ascari, Angela Marzella, Giuseppina Giulino, Antonia Lavinia Zuliani, Ubaldo Marra, Carolina Bobbio, Roberto Santoro, Cristina Carruezzo, Riccardo Ciucciarelli, Guido Turi, Laura Antolino, Manuela Brighi, Domenico Giannotti, Andrea Bottari, Gianmario Poto, Elisabetta Panduri, Emilio Giorgio Guerra, Francesco Renzi, Alessandro Puzziello, Elio Donnarumma, Francesco Roca, Antonio Romanelli, Giovanni Fabbrocile, Alessandro Liguori, Silvia Palmisano, Manuela Mastronardi, Beatrice Modesti, Lucia Comuzzi, Nicolò de Manzini, Biagio Casagranda, Edoardo Osenda, Valeria Tonini, Lodovico Sartarelli, Wladimiro Siciliano, Angelo Antonio Ciccarese, Claudia Mongelli, Yuri Macchitella, Andrea Barina, Giacomo Ghio, Aurora Iris Giacomello, Edoardo Segato, Carola Cenzi, Nicola Passuello, Alice Pecorino, Federico Soldan, Alessandro De Cassai, Nirvana Maroni, Caterina Froiio, Silvia Sala, Andrea Galimberti, Andrea Pisani Ceretti, Daniela Rega, Ernesto De Giulio, Pasqualina Perrotta, Francesca Bifulco, Paolo Delrio, Carmela Cervone, Silvia de Franciscis, Gennaro Martines, Giovanni Tomasicchio, Pellegrini Alessandro, Pappagallo Leonardo, Andrea Muratore, Marcello Calabrò, Cristina Valentino, Alberto Modini, Giulia Marchiori, Sofia Gamba, Patrizia Marsanic, Ciferri Enrico, Longhin Roberta, Epis Lorenzo, Emiliana Parodi, Barberis Andrea, Rossi Lisa, Antonio Amato, Paola Batistotti, Vitantonio Cirillo, Tiziana D'amato, Davide Cuneo, Ruggero Bollino, Helen Yu, Carla De Carlo, Andrea Viani, Giuseppe Evola, Ettore Gula, Carla Di Stefano, Marco Vacante, Luigi Piazza, Andrea Leonardi, Salvatore Lo Bianco, Gaya Spolverato, Francesco Celotto, Andrea Mingoli, Pierfrancesco Lapolla, Giuseppina Carta, Francesco Pugliese, Gioia Brachini, Simona Meneghini, Marco Milone, Anna D'Amore, Salvatore D'Angelo, Carmine Lacovazzo, Pietro Anoldo, Michele Manigrasso, Giovanni Domenico De Palma, Luigi Zorcolo, Simona Deidda, Giulia Murgia, Francesca Piras, Angelo Restivo, Valerio Argiolas, Ugo Grossi, Patrizia Pelizzo, Massimo De Paoli, Paolo Zanatta, Giulio Aniello Santoro, Eleonora Mollica, Marco Piccino, Giovanni Gambino, Rizzo Giovanna, Bertino Vanessa, Moncada Valentina, Michele De Capua, Jacopo Andreuccetti, Alessio Giordano, Davina Perini, Pasquale Punzo, Lorenzo Foti, Francesca Cammelli, Maximilian Scheiterle, Jacopo Martellucci, Carcato Marco, Gabriella Teresa, Capolupo, Alessandra Inzeo, Renato Ricciardi, Filippo Carannante, Gianluca Costa, Valentina Miacci, Vincenzo Lizzi, Nicola Tartaglia, Anna Maria Cairelli, Antonella Cotoia, Alessandra Giuliani, Giovanna Pavone, Mario Pacilli, Matteo Viti, Valentina Rampulla, Antonella Bosco, Zoltan Ghenkis Lance Eric Varga, Marco Mariani, Federica Ferracci, Ilaria Tersigni, Giusy Fortunato, Fabio Pacelli, Claudio Fiorillo, Lodovica Langellotti, Roberto Persiani, Alberto Biondi, Anna Paola Sanesi, Paola Aceto, Laura Lorenzon, Roberto Pezzuto, Flavio Tirelli, Francesco Quaglino, Federico Festa, Salome Pfannkuche, Antonella Evangelista, Luca Cestino, Alex Bruno Bellocchia, Beatrice Degan, Guglielmo Clarizia, Alessandro Longhini, Federica Giossi, Veronica Raveglia, Alessandro Spolini, Emanuele Asti, Pamela Milito, Tiziana Nania, Luca Di Girolamo, Daniele Tiziano Bernard, Stefano Siboni, Marco Sozzi, Massimiliano Di Paola, Chiara De Lucia, Barbara Proietto, Eugenia Puleo, Luca Giordano, Gherardo Romeo, Nunzio Maria Angelo Rinzivillo, Luigi Masoni, Matteo Cinquepalmi, Alessandra Pastone, Luca Ruggeri, Federico Maggi, Giulia Lauteri, Giovanni Guglielmo Laracca, Silvio Guerriero, Zuleyka Bianchi, Chiara Di Clemente, Silvia Di Rosa, Raffaele Galleano, Elena Ronda, Emanuela Massa, Paolo Minuto, Omar Ghazouani, Bianca Angelone, Marco De Prizio, Lorenzo Maria Fatucchi, Michela Boncompagni, Sara Alunno, Riccardo Malatesti, Alessio Mazzoni, Rezart Sulce, Camillo Leonardo Bertoglio, Lorenza Zampino, Paola Lampugnani, Giancarlo Razionale, Antonella Nisi, Martina Pardo, Edoardo Saladino, Giuseppe Cuticone, Elisa Bertilone, Orazio Bonanno, Maurizio Ronconi, Silvia Casiraghi, Magda Manfredini, Fabio Carbone, Roberto Santalucia, Anna Platto, Amerigo Galla, Simona Borin, Marco Realis Luc, Mariano Fortunato Armellino, Francesco Guida, Sara di Matteo, Serena Testa, Roberta Abete, Gianpaolo Marte, Andrea Tufo, Diletta Cassini, Chiara Limongi, Elisabetta Confalonieri, Stefano Clementi, Francesca De Stefano, Vincenzo Tondolo, Gianluca Rizzo, Luca Emanuele Amodio, Stefano Zaccone, Dario Sarli, Giovanna Cosetta, Filippo Alberghini, Andrea Pierre Luzzi, Giovanni Spiezio, Marina Pighin, Stefano Amore Bonapasta, Ilaria Peluso, Viola Tordi, Emiliao Mantovani, Matteo Gregori, Simone Santoni, Marco Venza, Stefania Angela Piccioni, Ludovico Carbone, Elena Boffa, Franco Roviello, Francesco Fleres, Carmelo Mazzeo, Serena Recupero, Vincenzo F Tripodi, Teresa Sinicropi, Santino A Biondo, Eugenio Cucinotta, Mario Annecchiarico, Giulio Argenio, Antonio Varricchio, Claudia Cirillo, Francesco Carafa, Nicola Cillara, Antonello Deserra, Maria Provenzano, Nicola Scalas, Francesca D'Agostino, Angela Pezzolla, Annunziata Panebianco, Lulia Lucarelli, Angela Di Ciaula, Ferdinando Massimiliano Anelli, Mara Sblendorio, Mario Testini, Giovanna Di Meo, Arianna Pontrelli, Angela Gurrado, Francesco Paolo Prete, Alessandro Pasculli, Norma Depalma, William Sergi, Federica Cagnazzo, Nicolas Adam, Marcello Giuseppe Spampinato, Stefano D'ugo, Claudia De Giorgi, Christian Cotsoglou, Beatrice Torre, Loredana Monina, Francesco Curto, Massimiliano Veroux, Roberta Granata, Ludovica Alessandro, Luigi La Via, Giordana Riccioli, Marianna Scribano, Costanza Distefano, Matteo Rottoli, Alice Gori, Gianni Di Croce, Andrea Zanoni, Giacomo Calini, Stefano Cardelli, Nicola Greco, Guido Mantovani, Marianna Cippini, Federica BenazziI, Antonio Francipane, Luigi Boccia, Aaanlisa Pascariello, Alberto Porcu, Teresa Perra, Vincenzo Pazzola, Alessandra Grazia Giuseppina Garau, Silvia Oggianu, Nicolò Canzoneri, Francesca Sassu, Marco Palucci, Celeste Del Basso, Gabriele Viticchié, Mirco Leo, Igor Monsellato, Gianluca Buzzi, Simone Targa, Barbara Mantovan, Mara Olga Bernasconi, Andrea Sanna, Andrea Rizzi, Simone Gianazza, Sonia Costabeber, Luca Botta, Luca Broggi, Marco Corbella, Mariacarla Besozzi, Dario Scala, Valerio Sciascia, Stefania Gregorio, Crescenza Guida, Francesco Bianco, Alessandra Novi, Rosario Esposito, Mario Salvatelli, Antonio Cappiello, Simona Gili, Ersilia Corrado, Antonio Zanghi, Andrea Cavallaro, Mariangela Giummarresi, Luigi La Via, Alessandro Cappellani, Simone Di Majo, Massimiliano Mistrangelo, Francesca Farnesi, Francesca Gioia, Gerardo Cortese, Elisabetta Seno, Marco Ettore Allaix, Mario Morino, Akihiko Horiguchi, Takahiro Tashiro, Hiroshi Komekura, Hiroyuki Kato, Takayuki Ochi, Toki Kawai, Omar Said Mansour, Yazan Alnairah, Orwa Aladaileh, Yaman Al Ghunimat, Younes Shatat, Fadi Maaita, Mohammad Debian, Tamer Almoumani, Sadeel Alrawashdeh, M S El Muhtaseb, Lana Al-Sabe, Bourhan Alrayes, Sief-Addeen Al-Tahayneh, Anas Alhjooj, Ahmad Alsamnah, Suhel Batarseh, Yanof Al-Naggar, Mohammad Salah, Ayoub Akwaisah, Ahmed Ahmayda, Arwi Omar Kara, Alhassan Ahmed Kashbour, Alhussen Ahmed Kashbour, Faraj Abdurabbah Faraj, Mohammed Younus Alabeedi, Mohamed Moftah Shanana, Amal Emran Khalleefah Alwirfili, Wanesa Abraheem, Abduljalel Abdulkarim Alzwai, Amira Hamad, Narjis Husayn, Asraa Alwirfili, Abtisam Alharam, Nagat Milad, Fatimah Alzahra Abduljawad, Hibah Bileid Bakeer, Farah Alshaeri, Malak Alkuwafe, Hawa Beheih, Eman Alkubti, Sharefa Al Teerah, Hibah Bileid Bakeer, Haitam Shames, Aya Mohammed Alqaarh, Hashim Aborkhis, Haitam Shames, Akram Alkaseek, Laila Shalabi, Sarah Aldressi, Mohammed Aziz, Hussin Boaza, Hakim Ibrahim, Naji Elshelmani, Mohamed Alsori, Reem Ghmagh, Rawia Jamal Ghmagh, Abdulqudus Deeknah, Eman Abdulwahed, Entisar Alshareea, Yamen Mohammed, Monia Ramadan, Ghazalah Masoud, Haeba Khteab, Antisar Almahdi Aeqaab, Marwa Saleh, Najwa Swidek Hamed, Ahmed Abdurrahman Said Algeblawi, Adnan Shareif, Mostafa Shafeik, Laila Aoheda, Talat Abu Salem, Mohamed Abed, Mohamed Basha, Mahmoud Alorani, Saifaleslam Elsahli, Ezzeddin Elfagih, Amal Abdelaziz, Malak Baie, Hajer Abusnina, Abdulrahman Yousf Jaber, Svitlana Cheshkivska, Bashir Albakosh, Majdolin Almahjoub, Najat Ben Hasan, Marway Iqreewi, Saulius Svagzdys, Henrikas Pauzas, Rima Budrikienė, Andrius Macas, Tadas Latkauskas, Paulius Lizdenis, Justas Zilinskas, Mohd Syakir Mohd Azahar, Muhammad Anwar Azrin, Nurul Azmeera Zamali, Collins Chong, Hizami Amin Tai, Nik Qisti Fathi, Mohd Hady Shukri Abdul Satar, April Camilla Roslani, Sui Weng Wong, Norhayati Mohd Hashim, Pui San Loh, Nora Abdul Aziz, Mohamed Rezal Abdul Aziz, Ruben Gregory Xavier, Sanjay Dev Singh, Mohamad Akmal Aizat Bakhori, Arbayah Laluroh, Pok Xiang Yan, Mohammad Izwan Isa, Jothinathan Muniandy, Koh Chee Keong, Marcus Gabriel Pitchai Joseph, Nanthini Ganapathy, Chetawati Binti Abu, Andee Dzulkarnaen Zakaria, Ong Xin Zen, Kamal Ariff Isa, Mohd Zulfakar Mazlan, Zaidi Zakaria, Wan Mokhzani Wan Mokhter, Mohd Nizam Md Hashim, Nabil Mohammad Azmi, Zairul Azwan Mohd Azman, Nur Afdzillah Abdul Rahman, ⁠Ismail Sagap, ⁠Soma Balaganapthi, Diana Melissa Dualim, Josephine Psaila, Sarah Bowman, Marianne Debono, Anne-Marie Camilleri-Podesta, Predrag Andrejevic, Charles Cini, Matthew Sammut, Oscar Posadas Trujillo, Danilo Tueme de la Peña, Diana Estrada Gutiérrez, Diana Díaz Arizmendi, Noel Salgado Nesme, Oscar Santes Jasso, Alberto Nájera Saldaña, Francisco Emmanuel Alvarez-Bautista, Mario Trejo-Avila, Erika Hernández-Montiel, Jorge M Antolinez-Motta, Alejandra Nuñez-Venzor, Roberto Punin-Neira, Asya Zubillaga-Mares, Jesus Alonso Valenzuela Perez, Juan Carlos Cardozo Aguilar, Patricia Esquivel de Alba, Edson Roberto Zendejas Avelar, Emmanuel Guadalupe Gutierrez Lopez, Jaime Alejandro Florian Lopez, Javier Luna Garcia, Carlos M Nuño-Guzmán, Luis Bravo-Cuéllar, María del Rosario Rojas-Martínez, Elvia Rocío Flores-Fonseca, Alejandro González-Ojeda, Sergio Vázquez-Sánchez, Kathia Morfin-Meza, Evelia Romo Ascencio, Clotilde Fuentes-Orozco, Luis Suarez Carreón, Edgar Cortes Torres, Joop Konsten, Maarten van Heinsbergen, Danique Smets, Sjef Schlooz, Muhammad Daniyan, George Duke Mukoro, Joy Uwanamodo, Idris Mohammad, Bashir Mohammed, Stephen Gana, Linus Ukwubile, Jerry Gofrey Makama, Bashiru Yeshua Aminu, Samaila Joshua, Ifeanyi Kene Aghadi, Stephen Akau Kache, Joseph Dekah, Damai Chinyio, Michael Aghahowa, Kayode Olofin, Eunice Olokun, Rosemary Nwokorie, Bata Gali, Muslimat Alada, Okechukwu Hyginus Ekwunife, Benedict Mmuodumogu, Chidiogo Ezennwa, Cyril Nwachukwu, Ochonma Egwuonwu, Chiemelu Emegoakor, Kenneth Ugwuanyi, Ahmed Siddique Ammar, Ahmad Faraz, Muhammad Faisal Khan, Farhat Nazneen, Sayed Shah Husnain, Muhammad Attaullah Khan, Ayaz Gul, Ihtisham ul Huq, Syed Ali Naqi Zaidi Zaidi, Ihsan Baloch, Adnan Ali Memon, Adeel ur Rehman, Ghina Awais, Ghina Shamsi, Nazish Iqbal, Reinaldo Isaacs Beron, Mariela Hurtado, Yazmin Arauz, Renata Castaño, César Huaroto Landeo, Juan Carlos Luna Cydejko, Maria Amalia Espinoza Huamán, Carolina Picasso Arias, Wiktor Krawczyk, Joanna Kosciolek, Genowefa Greif, Aleksandra Pluta, Jakub Wantulok, Andrzej Organ, Michał Nycz, Karolina Snopek-Miśta, Monika Rozkoszny, Ewa Musioł, Mariusz Kryj, Dariusz Waniczek, Paweł Musialski, Jakub Migoń, Michał Bąk, Adrianna Pielech, Wiktoria Kulińska, Jan Nicikowski, Manuel Rosete, Rita Andrade, Leandro Ferreira, Ana Luisa Almeida, Catarina Lopes, Inês Prior, Tiago Antunes, Beatriz Caldeira, Inês Matias, Cristina Ribeiro, Mariana Silva, Manuel Cotovio, Joana Bolota, Sofia Leandro, Vânia Fernandes, Marta Martins, Carla Rebelo, Nuno Ribeiro, Sílvia Silva, Álvaro Gonçalves, Inês Romero, André Caiado, João Maciel, Sandra Paranhos, Ana Gaspa, Diogo Gomes, Pedro Custódio, Maria José Duarte, José Guerreiro, Rodrigo Nemésio, Isabel Paixao, Jorge Balaia, Pedro Santos, Paula Magro, Célia Carvalho, Francisco Neves, João Martins, Bernardo Patrício, Teresa Carvalho, Hélder Além, Sofia Reis, Ana Carvalho, Alda Martins, Sara Patrocínio, Janine Carmelino, Frederico Nazareth, Filipe Ribeiro, Ana Moreira, Margarida Ventura, Catarina Bastos, Maria Costa, Maria Reigota, Maria Carvalho, Sofia Boligo, Gonçalo Figueirôa, Letícia Ribeiro, Daniela Zuzarte, Guilherme Fialho, Hugo Capote, Hermínia Telo, Júlio Soares, Cristina Costa, Sara Morais, Tamiris Mogne, Sara Brás, Ana Catarina Pinho, Vanessa Sousa, Ricardo Dias, Sofia Pina, Beatriz Mendes, Ana Sanches, Gonçalo Mesquita, Juan Rachadell, Edgar Amorim, Miguel Cunha, Andreia Ferreira, Jéssica Ricardo, Maria João Martins, Nuno Matias, Mariana Claro, Alberto Silva, Ana Nunes Vieira, Ana Cláudia Soares, Alexandra Terra, Ana Rita Pinheiro, Diogo Galvão, Débora Melo, António Mora, Sonia Fortuna Martins, Helena Devesa, Susana Pepino, Alberto Roxo, Sonia Marques, Renato Barradas, João Nobre, Patrícia Bárbara, Inês Damásio, Cátia Domingues, Inês Sousa, Maria Inês Coelho, Fernando Melo, Sara Andrade, Natércia Santos, Ana Lopes, Daniela Pais, Inês Colaço, Margarida Luís, Pedro Caldes, João Andrade, Paula Nobre, Ana Rita Saraiva, Maria Adelaide Campos, Sónia Bispo, Maria João Vale, Horacio Perez, Joana Peliteiro, Marina Ângelo, Alexandra Amaral, Rui Rainho, Vanessa Bettencourt, Tatiana Neves, Carlos Serra, Bernardo Maria, Beatriz Silva, João Barbosa, Pedro Febra, Rita Galama, Carla Pires, Sérgio Faria Baptista, Carlos Bôto, Maria Inês Seixo, Urânia Fernandes, Carolina Marques, Ana Isabel Rodrigues, Anabela Santos, Rita Marques, Ricardo Vaz-Pereira, Daniela Martins, Nicolae Bacalbasa, Irina Balescu, Maria Mandroc, Cristina Martac, Octav Ginghina, Andrei Bogdan Vacarasu, Ruxandra Pop, Sorela Radoi, Marius Zamfir, Mara Mardare, Irina Bondoc, Yuriy Voronin, Kazimagomed Zubailov, Marina Alexandrovna, Alexandr Butiaikin, Petr Tsarkov, Vladimir Balaban, Petr Tsugylya, Sergey Barkhatov, Inna Tulina, Valery Nekoval, Sergei Katorkin, Leonid Lichman, Vasilisa Kuper, Tatiana Shnitman, Pavel Andreev, Olga Davydova, Andrei Zhuravlev, Aleksei Karachun, Aleksandra Olkina, Marina Smolina, Sergey Rosengard, Evgeniy Zagaynov, Gleb Samsonov, Evgeniy Drozdov, Ker-Kan Tan, Kai Yin Lee, Siew Ching Wong, Ne-Hooi Will Loh, Chermaine Ang, Neng Wei Wong, Jin Yang Crystal Sia, Hehe Shang, Alex Joseph, Chi Yong James Ngu, Nan Zun Teo, Abdirahman Mohamed Osman, Abdikhaliq Koshin, Ahmed Abdirahman Hussein, Nasteho Mohamed Abdi, Mohamed Abdikarin Ismail, Ismael Mora-Guzman, Julian de Pedro, Santiago Alonso, Daniela Rodriguez, Alicia Rodriguez, Daniel Arcal, Virginia Jiménez Carneros, Guillermo Medina Sanchez, Maria Vanesa García Marrero, Susana Pretus Rubio, Ainhoa Valle Rubio, Alba Manuel Vazquez, Sara Gortazar de las Casas, María Luisa De Fuenmayor-Valera, Elena Viejo-Martinez, Begoña Toribio-Combarro, Javier Ripollés-Melchor, Javier Barambio-Buendía, Beatriz Fernández-Escudero, Felipe Acedo-Fernández de Pedro, Manuel Losada, Enrique Calcerrada, Ana García-Nuñez, Amaya Alcojor, Beatriz Diéguez, Alberto Vaquero, Alfredo Alonso, Victor Eduardo Gonzabay Campos, Alba Torroella Vallejo, Gloria Padillo, Alejandro Carvi, Cesar Ginesta Marti, Gabriel Cárdenas Rivera, Gustavo Mastroiani, Elena Sagarr Eisaa Cebolla, Eduardo de San Pío Carvajal, Cristina Palomar Puertas, Marta Cuartero Castro, Raquel Rios Blanco, José Manuel Muros Bayo, Eneida Bra Insa, Manuel Diez Alonso, Fernando Mendoza Moreno, Silvestra Barrena Blazquez, Javier Hernandez, Belen Matias, Alberto Vilar Tabanera, Alma Blazquez Martin, Mercedes Estaire-Gómez, María Dolores Cancelas-Felgueras, Marina de la Fuente-Rodríguez, José Miguel Valverde-Mantecón, Beatriz de Andres-Asenjo, Guillermo Cabezudo, Cristina Santos-Gómez, Andrea Sánchez-Miguel, Carlos Jezieniecki, Alejandro Romero, Andrea Vazquez, Patricia Tejedor, María Sánchez-Rodríguez, Gladys Anhuaman Centeno, Julio Orallo Martínez, Luis Miguel Jiménez Gómez, Elena Hurtado, Silvia Kayser, Milagros Carrasco, Isabel Jiménez, Pilar Martínez, Rebeca Celdrán, Pedro Parra, José Manuel Muñoz, María Ramírez, Ana Minaya-Bravo, Enrique González-Gonzalez, Mirian Marta Alba, Ana Sánchez Gollarte, Susana Prieto, Carlos Guijarro, Raul Díaz-Pedrero, Ana Minaya-Bravo, Veronica Ongil, Ernesto Josue Ochoa, Diego Cordova, Laura Jimenez, Ricardo Alvarado, David Moro-Valdezate, Vicente Pla-Martí, Celia San Antonio, Andrea Gutiérrez-Valcárcel, Stephanie García-Botello, José Martín-Arévalo, Leticia Pérez-Santiago, Pablo Calvo Espino, Maria Donat Garrido, Maria Concepción Olmo García, Ricardo Villamarín Tibaduiza, Javier Páramo Zunzunegui, Justyna Drewniak Jakubowska, Ana Isabel Ariza Ibarra, Héctor Aguado López, Beatriz Aguado Rodriguez, Maria Díaz Ruiz, Arelis Pérez Fernández, Francisco Javier Ruescas García, Andrés García Marín, Graciela Valero-Navarro, Victoriano Soria-Aledo, Rocio Garcia-Capitan, Fernando Garcia-Sanchez, Enrique Pellicer-Franco, Mónica Mengual-Ballester, Jose Andres Garcia-Marin, Selvaratnam Srishankar, Pramodh Chandrasinghe, Aniradha Neligama, Nayana Abeysinghe, Bhagya Gunetilleke, Dakshitha Wickramasinghe, Dileesha Wickramasinghe, Rasika Nilangani, Vihara Dasanayaka, Adam AbdAla, Baraa Ali, Ibrahim Ahmed, Mai Salah, Iman Abdalla, Sara Yousif, Samar Ali, Abuzaid Mohamedali, Yasen Kamal, Renad Fagery, Ehssan Mohamedelnazir, Mawada Abdulhadi, Mohammed Osman Ahmed Osman, Mohaned Mustafa, Ibrahim Nagmeldin Hassan, Khalid Alrwa, Omsalama Ibrahim, Mohamed Ibrahim, Abdulrahman Abouh, Mohammedbabalrahma Koko, Thuriya, Modawi, Mai Bushra, Abdalla Adam, Ahmed Idris, Ahmed Mohamed Ibrahim Mohamed, Mihad Abdalla Mahmoud, Khalid Omer, Yassin Moner, Bahaaldeen Osman Hassan Abker, Mohamed Abdalbagi Ahmed Ibrahim, Mohamed Musa Yassin, Hassan Salih Hussein Eisa, Mohamed Musa Yassin, Mohamed Almahdi, Aljarrah Mohammed, Mohamad Alhadi, Faihaa Siddig, Islam Daboka Fono, Alshima Siddig Mohamed Khalifa, Kamal Ali Mohammed Salih, Zeinab Siddig Ahmed Bakhiet, Anfal Yousif Ahmd Ezat, Mohamad Abdelbagi Sidahmed Ali, Samah Elhussein Mohamed Ahmed, Aziza Ibraheem Suliman Abdalla, ElSagad Eltayeb Gady, Mohanned Kamal Mahmoud Elmuzamil, Hossam Aldeen Alhareth, Ahmed Alsisi, Nehal Mohamed, Majdi Abuidris Abderrahim Dafa Allah, Ibrahim Mutwakil Gamal Ahmed, Mohaned Awad Ahmed Daoud, Waleed Abdalla Altayeb, Mustafa Khalifa Mohamed Kheir Hamed, Gamal Mutwakil Gamal Ahmed, Mazin Alzebeer, Hebatallah Fadhl, Muhammed Ali, Magdi Essam, Asmaa Altegani, Ibrahim Gareeb, Hadeel Omara, Sami Galal Eldin, Yusra Badri Elhuda Abdelaziz, Abd Elrahim, Razan Khalid, Abdelrahim Abdelrazig Ahmed Ibrahim, Sara Osman, Musab Hafiz, Asaad Ahmed, Yousef Hamed, Mohamed Hadal, Sanabil Mohamed, Ahmed Hassan, Yasmin Ammar, Najia Azhar, Carl-Viktor Hellberg, Jenny Lindgren, Silvia Marchesi, Pamela Buchwald, Valentinus Valdimarsson, Pernilla Hansdotter Andersson, Sofia Xenaki, Antonio Nocito, Alex Ochsner, Andreas Keerl, Andrea Wirsching, Hala Almobi Alsaid Mushaweh, Youssef Zeeb, Modar Al Ali, Oubada Elmolla, Mjd Al Khateeb, Samir Kanaan, Hussein Mohammad, Rawia Dooba, Mohammad Ibrahim, Hamed Tarboosh, Noor Haidar, Ammar Kanaan, Altayeb Alhaj Zain, Ahmad Alusef, Nawwar Reslan, Saleem Alabdullah, Hadi Alabdullah, Alaa Aldirani, Mohammad Alawir, Ehsan Albakri, Mouhamad Al-Khatib, Yulian Mhana, Mohannad Nasani, Jamal Houari, Diaa Mohammad Hamad, Mohammad Tammam Saffour, Nazeeh Alaktaa, Mohamed Alaktaa, Merei Aweni, Jaw-Yaun Wang, I-Chieh Lee, PengJen Huang, Pojung Chen, Anis Hasnaoui, Racem Trigui, Sherif Ben Salem, Mohamed Amine Sassi, Chaima Yakoubi, Mohamed Ali Mseddi, Raja Younsi, Chokri Elomri, Rami Guizani, Rakia Siala, Amine Sebai, Ahmed Ben Mahmoud, Mariem Ben Brahim, Souhaib Atri, Montasser Kacem, Anis Haddad, Houcine Maghrebi, Abdelkader Mizouni, Marwa Azri, Asma Ounaies, Habiba Ben Hamada, Mehdi Ben Latifa, Amal Bouazzi, Sami Lagha, Yunus Emre Altuntaş, Nail Can Adigüzel, Esra Kunduz, Mücahit Okçu, Omar Alomari, Muhammed Edib Mokresh, Gizem Duran, Alp Ömer Cantürk, Fatih Altıntoprak, Meryem Çelik, Ali Fuat Erdem, Erhan Eröz, Kayhan Özdemir, Banu Yigit, Serhat Meric, Guler Erol, Merve Gokce, Ahmet Sencer Ergin, Alparslan Saylar, Nihat Bugdayci, Elif Colak, Mine Gizem Bidil, Sadan Somuncuoglu Ozbilgin, Hale Kefeli Celik, Ahmet Burak Ciftci, Kursat Yemez, Huseyin Eraslan, Serdar Şenol, Korhan Tuncer, Gizem Kılınç Tuncer, Özge Yolcu, Hakan Aygün, Cem Tuğmen, Eyüp Kebapçı, Serdar Ünlü, Yunushan Furkan Aydoğdu, Ömer Kubat, Gizem Kılıç Aydoğdu, Burak Ersun, Semra Demirli Atici, Aras Emre Canda, Özgür Bolat, Mustafa Cem Terzi, Afag Aghayeva, Bilgi Baca, Ebru Kirbiyik, Hasan Cem Guneyli, Mustafa Ege Seker, Bengi Agca, Metincan Erkaya, Hüseyin Kerem Tolan, Enes Sertkaya, Sevgi Öztürk, Mustafa Deniz Dinkçi, Mert Gedik, Emre Furkan Kırkan, Muhammed Oğuz çelikkaya, Osman Bozbiyik, Bahadir Emre Baki, Demet Yurtsever, Alev Atalay, Cemil Caliskan, Erhan Akgün, Mustafa Korkut, Server Sezgin Uludağ, Ergin Erginöz, Dilara Kılınç, Şafak Emre Erbabacan, Seda Erdil, Serkan Akkuş, Mahmut Alper Güldağ, Ali Cihat Yildirim, Muhammed Alperen Taş, Hülya Zorbas, Serkan Telli, Sezgin Zeren, Cihangir Akyol, Mehmet Ali Koc, Selma Dincel, Suheyla Karadag Erkoc, Siyar Ersoz, Kaan Sunter, Selcuk Gulec, Fatma Ayca Gultekin, Yigit Ozaydın, Ozkan Guler, Keziban Bollucuoglu, Ufuk Tali, Veysel Barış Turhan, Ertuğrul Gazi Alkurt, Furkan Uğur, Murtaza Salih Kepez, Semin Turhan, Bahadır Kartal, Mehmet Yalvaç, Murat Kendirci, Fevzi Cengiz, Örgün Güneş, Meltem Ulus, Murat Aksun, Rıfat Peksöz, Muhammet Yıldırım, Nurbanu Uzunget, Ali Ahıskalıoğlu, Sabri Selçuk Atamanalp, Yavuz Albayrak, Can Sahin, Umut Emreol, Esra Gunduz, Semih Aydemir, Furkan Akin, Tahsin Colak, Cumhur Ozcan, Ruhnaz Okyay, Levent Ozdemir, Enver Reyhan, Hilmi Bozkurt, Mustafa Berkesoglu, Sami Acar, Cagil Karaevli, Merve Karanfil, Ahmet Gultekin, Ufuk Memiş, Mücahit Taş, Seçkin Karakus, Alparslan Koç, Ökkeş Sezai Leventoğlu, Denizcan Bozkurt, Şerife Naz Bozkurt, Metin Alkan, Ramazan Kozan, Aydın Yavuz, Ahmet Çağrı Büyükkasap, Tayfun Bisgin, Sevgi Deniz Köse, Sevda Özkardesler, Selman Sokmen, Berke Manoğlu, Caner Bektaş, Omer Faruk Ozkan, Merve Karadag, Ozlem Akoglu, Suheyla Abitagaoglu, Haluk Kerim Karakullukcu, Hanife Seyda Ulgur Genc, Hilmi Bora Isceviren, Ahmet Necati Sanli, Yusuf Iskender Tandogan, Erdem Durum, Deniz Altiok Tandogan, Alparslan Ertenlice, Stepan Grytsenko, Yurii Nedoshytko, Oksana Shymchuk, Rostyslav Yuryk, Andrii Beznosenko, Veronika Rozhkova, Olena Rybachuk, Ivan Lisnyy, Anatoli Shudrak, Vitaliy Zvirych, Sergii Podpriatov, Olexandr Ivanko, Nataliia Kudelia, Olena Levytska, Andrii Martynenko, Sergey Trepet, Oleksandr Hryb, Ievgen Ilin, Alina Semeniuk, Oksana Grusha, Genadij Farina, Victor Zhylchuk, Oleksandr Tkach, Ramy Shaalan, Claire Carden, Lauren Harper, Joanna Lynch, Tarek Khalil, Morven Stewart, Neville Ekpete, Jayan Jayasinghe, Joussi Ramzi, Pamela Buyson, Charlotte Dempsey, Mohamed Adnan Thaha, Annamaria Minicozzi, Prashanth Chowdary, Prem Thambi, Mohamed Mansour, Rebecca Metcalfe, Ahmed Elfoly, Peter Bennett, Parth Gada, Frank McDermott, Antonios Gklavas, Emily Fisher, Tom Clark, Rob Bethune, Polly Estridge, Priya Nandoskar, Ahmed Nada, Lahreb Aktar, Sophie Bennett, Christopher Harmer, Gary Nicholson, Mishal Shahid, Finlay Young, John-Patrick Byars, Aidan Doye, Mewhish Ansar, Claire Kilbride, Tom Kennedy, Rifat Mohamed Oluwaseun, Prashant Nair, Panna Pateel, Siddhartha Handa, Karen Robinson, Wael Abdelrhman, Ralph Obure, Chrysanthi Karagianni, Faris Mohammed Abdulwali Alhajami, Nezar Mohammed Mohammed Mahyoub, Sultan Ahmed Ibrahim Al-Hamidi, Hemier Mannsor Nasr Al-Shaif, Hussein Abdullah Ahmed Doan, Gamal Abdulrahman Khaled Al-Qubati, Mohammed Abdullah Aqlan Al-Hershi

## Abstract

**Background:**

Anastomotic leak (AL) is the leading cause of major morbidity after right colectomy, affecting 5–10% of patients and accounting for up to one-third of postoperative deaths. The multicentre EAGLE randomized trial demonstrated reductions in AL when surgeons completed a digital safe-anastomosis training package, but its effectiveness outside a trial setting remains untested.

**Methods:**

EAGLE-2 was an international prospective cohort study conducted across participating centres over an 8-week intervals (June–September 2024). Consecutive adults undergoing right colectomy surgery with primary anastomosis were included. The EAGLE safe-anastomosis training platform was made open access and surgeons were categorized according to whether they had completed the modules before surgery. The primary outcome was 30-day AL, defined as a defect of intestinal wall integrity at the anastomotic site leading to frank AL and/or intra-abdominal collection. Secondary outcomes included collection alone, training uptake, and implementation of the recommended checklist. Mixed-effects logistic regression adjusting for ASA grade, urgency, and intraoperative contamination was performed with hospital as a random effect.

**Results:**

A total of 2875 patients were recruited across 332 hospitals in 60 countries. Of these, 2242 (78.0%) operations were performed by trained surgeons and 633 (22.0%) by untrained surgeons. AL/collection occurred in 16.4% of the non-trained group *versus* 9.1% of the trained group, giving a difference of 7.3% (*P* < 0.001). Adjusted analysis found training was associated with almost a halving of the OR for AL (adjusted OR (aOR) 0.56 (95% c.i. 0.41 to 0.78)), as well as lower reoperation (aOR 0.64) and readmission (aOR 0.65) rates.

**Conclusion:**

Completion of a brief digital training programme was associated with a clinically and statistically significant lower rate of AL after right colectomy, alongside lower rates of reoperation, readmission, and death. Scalable digital education should be embedded into colorectal quality improvement programmes worldwide.

## Teaser

Adjusted analysis demonstrating a statistically significant reduction in anastomotic leak when the operating surgeon undertook the safe-anastomosis online learning modules.

## Introduction

Right hemicolectomy and ileocaecal resection are among the most commonly performed colonic procedures worldwide, reflecting the high global burden of colorectal disease, with colorectal cancer accounting for approximately 1.9 million new cases annually^1^, 20–40% of which involve the right colon^2^. Collectively termed ‘right colectomy’^3,4^, these operations are carried out by both general and colorectal surgeons^2^, for indications that commonly include malignancy, inflammatory bowel disease, volvulus, and trauma^2,4–7^. Despite being regarded as technically straightforward compared with left-sided resections, right colectomy carries a substantial risk of postoperative morbidity^5–9^. Anastomotic leak (AL) occurs in 5–10% of patients, the risk rising with patient co-morbidity, sepsis, and emergency surgery^2,5–9^. AL occurrence is associated with a ten-fold increase in the risk of postoperative death^5,6^. AL also impairs cancer-specific survival, increases local recurrence, and substantially worsens postoperative quality of life through prolonged recovery and permanent stoma formation^2,5,8^.

The 2015 European Society of Coloproctology (ESCP) audit of right colectomy and ileocaecal resection reported an overall AL and abdominal collection rate of 8.1%, alongside striking variation in surgical practice^5^. Fourteen distinct anastomotic configurations were identified, nine of which were used by <10.0% of surgeons^5,6^. Stapled anastomoses were associated with higher AL rates than handsewn joins, most notable in the emergency setting^6^. Surgeon specialism was associated with differing outcomes, with general surgeons having a 1.5-fold higher AL risk^5^. These findings suggested that variation in operative approach and training could be key drivers of better outcomes.

The Education and Assessment for Gastrointestinal Leak Elimination (EAGLE) RCT evaluated a digital educational package, the safe-anastomosis programme, in 332 hospitals across 64 countries worldwide^7,10,11^. This intervention provided the best current clinical evidence and introduced surgeons to three recommended interventions: a preoperative risk stratification calculator; evidence-based operative techniques; and a structured intraoperative safety framework. Centres in which >80% of surgeons completed the training saw the highest levels of intervention implementation and also the highest reduction in AL rate from 12.2% to 5.1%, which is a >50% reduction after adjusted analysis^7^.

However, despite these encouraging results, several questions remained unanswered. The EAGLE trial was delivered in a controlled research setting with active recruitment and structured oversight^7^. It was unclear whether busy surgeons would voluntarily complete the training outside such a framework and whether its benefits would persist across diverse, non-research, resource-varying environments.

The EAGLE-2 study was therefore designed as a pragmatic, independent, snapshot validation project. It sought to determine whether safe-anastomosis training would be undertaken outside a structured research setting and could lead to a reduced rate of AL in real-world clinical practice.

## Methods

### Study design and oversight

EAGLE-2 was an international, investigator-initiated, prospective cohort study conducted over intervals that spanned 8 weeks each between June and September 2024. The study was designed in accordance with the STROBE guidelines, for observational research, and is open access (*Table S1*). It formed part of the wider EAGLE initiative, coordinated by the ESCP and supported by the National Institute for Health and Care Research (NIHR) Global Surgery Unit^7,11–13^.

The study protocol was reviewed and approved by the ESCP Safe Anastomosis Steering Committee and the NIHR Global Health Research Unit in Surgery^12,13^. As an anonymized, collaborative audit of standard practice, the project met national service evaluation criteria in most countries; local ethical approval was obtained where required.

### Setting and participating centres

Hospitals were recruited through the ESCP membership, regional surgical societies, and open calls via professional networks and social media. All institutions performing right colectomy were eligible to participate, regardless of surgical volume, income level, or previous involvement in the EAGLE-1 trial. Each centre was required to register a named local lead responsible for team coordination, data validation, and ensuring consecutive patient inclusion during the study window.

### Eligibility criteria

Adults aged ≥18 years, planned for right hemicolectomy, ileocaecal resection or extended right colectomy with a single primary ileocolic or ileotransverse anastomosis, were eligible. Indications included malignant, inflammatory, or benign pathology such as cancer, Crohn’s disease, volvulus, or trauma. Both elective and emergency procedures were included, irrespective of operative approach (open, laparoscopic, robotic, or converted).

Patients were excluded if an anastomosis was not planned (planned stoma formation), if more than one intestinal anastomosis was created, if right colectomy was combined with multivisceral resection, cytoreductive surgery, or hyperthermic intraperitoneal chemotherapy (HIPEC), or if synchronous colorectal resections were undertaken. Patients could only be enrolled once during the study interval.

### Intervention and exposure

The exposure of interest was completion of the safe-anastomosis training modules by the operating surgeon. This digital training programme was developed by the ESCP and the NIHR Global Surgery Unit as part of the EAGLE educational initiative^12,13^. It was made freely accessible at https://www.escp.eu.com/eagle/introduction for the purposes of this cohort study. It consisted of five interactive e-learning modules within an online learning environment.

Each module included video demonstrations, case-based questions, and self-assessment components, requiring approximately 90 min to complete in total. Surgeons were required to declare if they had completed training at the time of surgery; those who had not were categorized as having ‘not completed training’. This was confirmed and completed by the in-theatre case report form (CRF) completed contemporaneously by the theatre team.

### Study rationale and objectives

The EAGLE-2 audit was designed as a pragmatic, global validation study to assess the effectiveness of safe-anastomosis training in real-world surgical practice and to assess associations between training completion and key postoperative outcomes. The primary objective was to determine whether completion of the training by the operating surgeon was associated with a lower rate of AL after right colectomy.

### Outcomes

The primary outcome was 30-day AL, defined as a ‘defect of the intestinal wall at the anastomotic site (including suture and staple lines of neorectal reservoirs) leading to a communication between the intra- and extraluminal compartments, with a pelvic abscess adjacent to the anastomosis also considered an AL (with or without demonstrated communication)’^5,14^. This definition aligned with ESCP consensus and the Centers for Disease Control (CDC) criteria for organ-space infection^15^. Leak and intra-abdominal collection were combined because postoperative collections may represent clinically relevant occult or contained anastomotic failure after right colectomy.

Secondary clinical outcomes included intra-abdominal collection without proven leak, unplanned critical care admission, 30-day reoperation, hospital readmission, postoperative length of stay, and all-cause 30-day mortality. This is in line with multiple previous studies for right colectomy^5–7^.

### Data collection and validation

Data were prospectively entered into a secure REDCap database (a secure, web-based software platform created at Vanderbilt University used worldwide to build and manage online surveys and databases for clinical and academic research) hosted by the University of Birmingham. The electronic CRF was structured into four phases: preoperative demographics and co-morbidities; operative details; postoperative complications; and outcomes at 30 days. Each site designated at least two collaborators responsible for data collection and verification.

Built-in range and logic checks ensured data accuracy, with mandatory field completion preventing missing critical variables. Sites with >5% missing data of key outcome variables were asked to complete the missing data. If they persistently were unable to complete the missing data, they were excluded from analysis to prevent unintentional bias.

Data governance adhered to the General Data Protection Regulation (GDPR) and local data protection frameworks. All data were anonymized before submission and unique study identifiers were used in place of patient identifiers.

### Sample size

Sample size was based on previous ESCP audits indicating a baseline leak rate of 8–10%^5^. The absolute AL rate in the EAGLE-1 trial was 10.1% (170 of 1691) during data collection before the intervention. It was anticipated that the leak rate for this study would be around 8% as the majority of the participating surgeons would have completed the training modules. It was assumed that 8% of the participants in the population would have an AL, therefore the study would require a sample size of 2828 for estimating the expected proportion with 1% absolute precision and 95% confidence. It was assumed that 5% would be lost to follow-up, giving a total of 2977 participants that would be required. EAGLE-1 collected data for 3039 patients who underwent right colectomy, submitted by 355 hospitals. Each collected data over a 2-month interval. This equated to a mean of 8.5 patients per 2 months. A mean of 8.5 patients per hospital was required for each 2-month recruitment interval, which was around 1 patient per week.

### Statistical analysis

Descriptive statistics are used to summarize patient characteristics and outcomes. Categorical variables are summarized as *n* (%) and were compared using a chi-squared test or Fisher’s exact test, whereas continuous variables are summarized as median (interquartile range (i.q.r.)) and were compared using a Mann–Whitney *U* test or a Kruskal–Wallis test.

The association between training completion and AL was assessed using mixed-effects logistic regression, adjusting for ASA grade (I–II *versus* III–V), urgency of surgery (emergency *versus* elective), and intraoperative contamination (clean/clean-contaminated *versus* contaminated/dirty). Hospital was entered as a random intercept.

All analyses were performed using ‘Stata 18’ (StataCorp, College Station, TX, USA) and ‘R 4.3.0’ (R Foundation for Statistical Computing, Vienna, Austria). Statistical significance was set at *P* < 0.050.

## Results

### Study population

**Figure 1:**
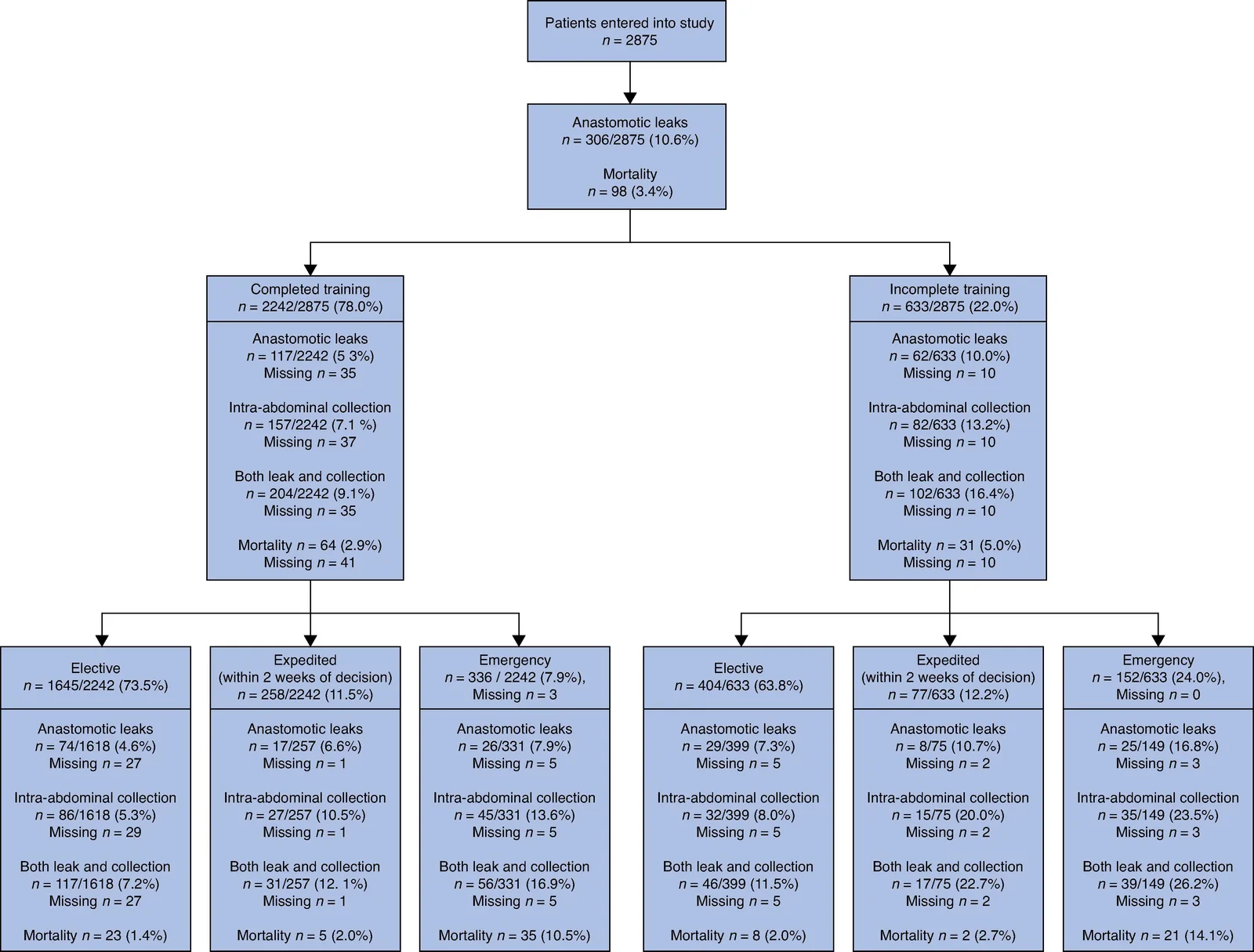
Study flow diagram—showing patient inclusion and final analysis population

A total of 2875 patients were recruited across 332 hospitals in 60 countries (*Table S2*, *Fig. 1*, and *Fig. S1*), between 1 June 2024 and 30 September 2024. No hospitals had missing data of >5% and therefore no hospitals were excluded from this analysis. Of the 332 hospitals, 209 hospitals recruiting 1810 of 2875 patients (63.0%) had not taken part in EAGLE-1. The median age was 67 (i.q.r. 54–76) years and 45.4% were female. Common co-morbidities included diabetes in 23.2%, ischaemic heart disease or cerebral vascular accident in 21.4%, and obesity (BMI >30 kg/m^2^) in 20.2% (*Table 1*). Malignancy was the indication for surgery in 78.3% of cases (*Table 2*). Emergency procedures accounted for 17.0% of procedures. Minimally invasive surgery (laparoscopic or robotic surgery) was used for completion of the operation in 47.6% of operations, and 7.5% were converted to open. Baseline and operative characteristics were well balanced between patients operated on by EAGLE-trained and non-trained surgeons, except that those presenting for emergency surgery were over-represented in the ‘untrained’ cohort (152 (24.0%) *versus* 336 (15.0%)) (*Table 1*, *Table 2*, and *Table S3*).

**Table 1:** Baseline patient and operative characteristics according to whether the surgeon completed or did not complete training

Factors	Levels	Operating surgeon did NOT complete training (*n* = 633)	Operating surgeon completed training (*n* = 2242)	Total (*n* = 2875)	*P*
Age (years)	Median (i.q.r.)	66.0 (54.0–76.0)	67.0 (54.0–76.0)	67.0 (54.0–76.0)	0.772
	Missing (*n*)	0	0	0	
Sex	Female	291 (46.0)	1014 (45.2)	1305 (45.4)	0.774
	Male	342 (54.0)	1228 (54.8)	1570 (54.6)	
	Missing (*n*)	0	0	0	
Previous abdominal surgery	No	386 (61.0)	1374 (61.3)	1760 (61.3)	0.906
	Yes	247 (39.0)	866 (38.7)	1113 (38.7)	
	Missing (*n*)	0	2	0	
BMI >30 kg/m^2^	No	503 (79.5)	1791 (80.0)	2294 (79.8)	0.828
	Yes	130 (20.5)	449 (20.0)	579 (20.2)	
	Missing (*n*)	0	2	0	
Known diabetes*	No	506 (79.9)	1701 (76.0)	2207 (76.8)	0.042
	Yes	127 (20.1)	538 (24.0)	665 (23.2)	
	Missing (*n*)	0	3	3	
History of IHD or CVA	No	501 (79.1)	1756 (78.4)	2257 (78.6)	0.724
	Yes	132 (20.9)	484 (21.6)	616 (21.4)	
	Missing (*n*)	0	2	0	
Oral anticoagulants†	No	523 (82.6)	1923 (85.9)	2446 (85.2)	0.048
	Yes	110 (17.4)	316 (14.1)	426 (14.8)	
	Missing (*n*)	0	3	3	
Preoperative total protein level (g/dl)	≤5.9	158 (25.0)	708 (31.6)	867 (30.1)	0.108
	6–6.9	139 (22.0)	645 (28.8)	784 (27.2)	
	≥7	163 (25.8)	591 (26.4)	754 (26.2)	
	Missing (*n*)	173 (27.3)	298 (13.3)	474 (16.5)	
Preoperative haemoglobin (g/dl)	<79	21 (3.3)	84 (3.8)	105 (3.7)	0.179
	80–99	142 (22.4)	467 (20.8)	609 (21.2)	
	100–119	191 (30.2)	774 (34.5)	966 (33.6)	
	120–139	176 (27.8)	615 (27.4)	791 (27.5)	
	≥140	97 (15.3)	284 (12.7)	381 (13.2)	
	Missing (*n*)	6 (1.0)	18 (0.8)	27 (0.9)	

Values are *n* (%) unless otherwise indicated. *Diagnosis of diabetes on diet management only, oral medication or subcutaneous treatment. †Any treatment taken orally/subcutaneously or intravenously that is aimed at thinning blood therapeutically. IHD, ischaemic heart disease; CVA, cerebral vascular accident.

**Table 2:** Operative characteristics of patients operated on by trained *versus* non-trained surgeons

Factors	Levels	Operating surgeon did NOT complete training (*n* = 633)	Operating surgeon completed training (*n* = 2242)	Total (*n* = 2875)
Timing of surgery	Elective (planned)	404 (63.8)	1645 (73.5)	2049 (71.3)
	Expedited (within 2 weeks of decision)	77 (12.2)	258 (11.5)	335 (11.7)
	Emergency (unplanned)	152 (24.0)	336 (15.0)	488 (17.0)
	Missing (*n*)	0	3	3
Indication for surgery	Malignancy	464 (73.4)	1784 (79.6)	2248 (78.3)
	Inflammatory bowel disease	53 (8.4)	179 (8.0)	232 (8.1)
	Other	115 (18.2)	277 (12.4)	392 (13.6)
	Missing (*n*)	1	2	3
Bowel preparation	None	355 (56.1)	1117 (49.9)	1472 (51.2)
	Mechanical bowel preparation only	162 (25.6)	520 (23.2)	682 (23.7)
	Mechanical bowel preparation with oral antibiotics	116 (18.3)	603 (26.9)	719 (25.0)
	Missing (*n*)	0	2	2
ASA grade	I–II	347 (54.8)	1345 (60.0)	1693 (58.8)
	III–V	286 (45.2)	895 (40.0)	1183 (41.1)
	Missing (*n*)	0	2	2
Primary operating surgeon	Consultant colorectal surgeon	246 (38.9)	1245 (55.6)	1491 (51.9)
	Trainee colorectal surgeon	47 (7.4)	203 (9.1)	250 (8.7)
	Consultant general surgeon	261 (41.2)	620 (27.7)	881 (30.7)
	Trainee general surgeon	79 (12.5)	172 (7.7)	251 (8.7)
	Missing (*n*)	0	2	2
Operative approach	Open	309 (48.8)	979 (43.7)	1288 (44.8)
	Laparoscopic (completed)	245 (38.7)	939 (41.9)	1184 (41.2)
	Laparoscopic (converted to open)	47 (7.4)	160 (7.1)	207 (7.2)
	Robotic (completed)	32 (5.1)	152 (6.8)	184 (6.4)
	Robotic (converted to open)	0 (0.0)	10 (0.4)	10 (0.3)
	Missing (*n*)	0	2	2
Operative field contamination	Clean-contaminated	527 (83.3)	1961 (87.5)	2488 (86.6)
	Contaminated	67 (10.6)	201 (9.0)	268 (9.3)
	Dirty	39 (6.2)	78 (3.5)	117 (4.1)
	Missing (*n*)	0	2	2
Anastomosis formed	Stapled	404 (63.8)	1491 (66.6)	1895 (66.0)
	Handsewn	162 (25.6)	566 (25.3)	728 (25.3)
	No anastomosis	67 (10.6)	183 (8.2)	250 (8.7)
	Missing (*n*)	0	2	2

Values are *n* (%) unless otherwise indicated.

### AL rate

**Figure 2:**
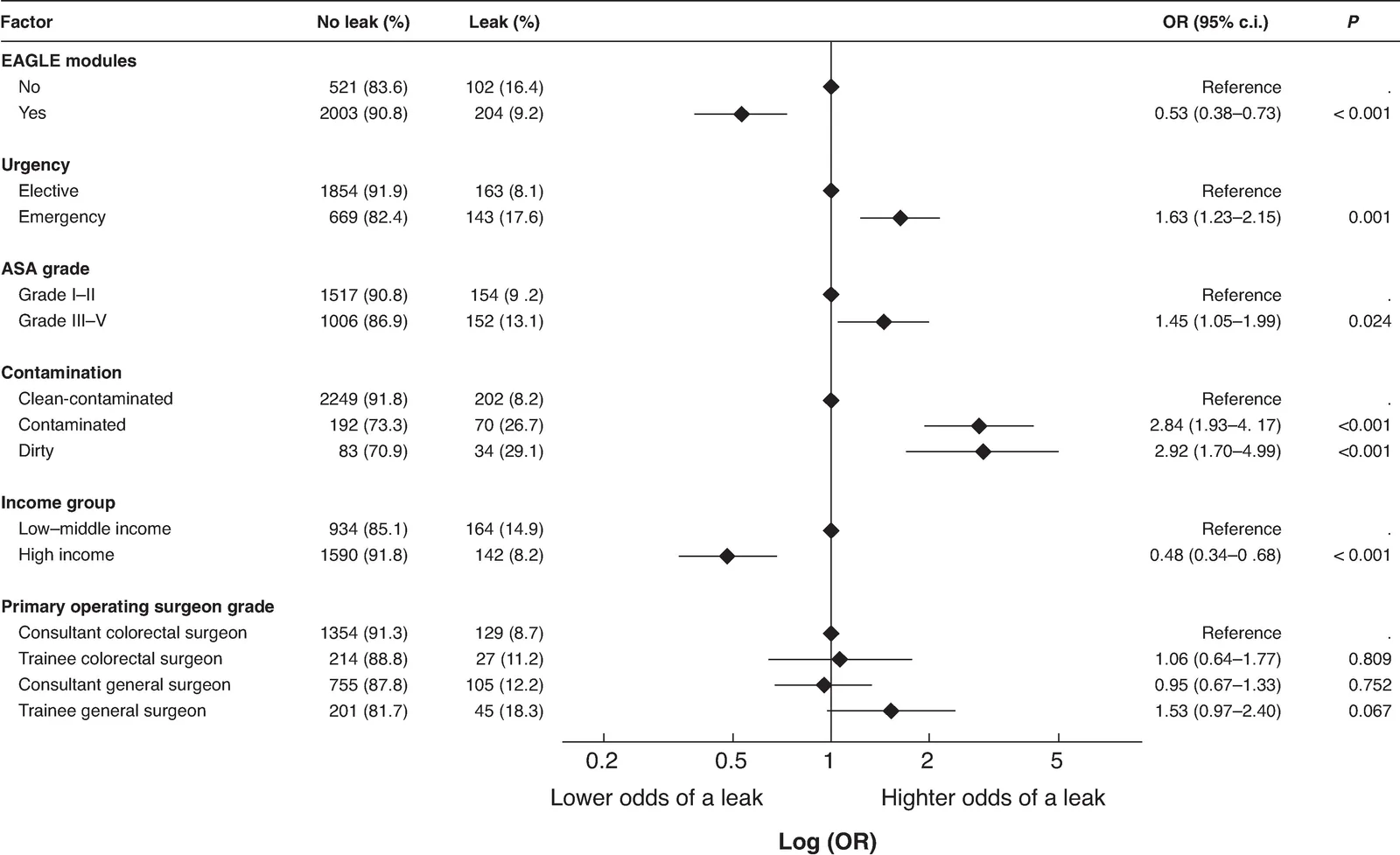
Forest plot of subgroup analyses—displaying ORs for anastomotic leak according to urgency, ASA grade, contamination, income group, and training status

The overall 30-day AL rate (including intra-abdominal collections) was 10.8% (306 of 2830). There were 633 (22.0%) operations performed by surgeons who had not previously undertaken the training with an AL rate of 16.4% (102 of 623). There were 2242 (78.0%) operations performed by surgeons who had completed the safe-anastomosis training with an AL rate of 9.1% (204 of 2207), representing a difference of 7.3% (*P* < 0.001). After adjustment for pre-specified confounders, including ASA grade (I–II *versus* III–V), urgency of surgery (emergency *versus* elective), and intraoperative contamination (clean/clean-contaminated *versus* contaminated/dirty), completion of the online EAGLE training was associated with halved odds of leak (adjusted OR (aOR) 0.56 (95% c.i. 0.41 to 0.78)) (*Table 3* and *Fig. 2*). These findings were consistent across elective and emergency procedures (*Table S4*).

**Table 3:** Clinical and operative outcomes

Factors	Levels	Operating surgeon did NOT complete training (*n* = 633)	Operating surgeon completed training (*n* = 2242)	Total (*n* = 2875)	*P*
Postoperative critical care admission	None	429 (68.9)	1543 (69.9)	1972 (69.6)	0.002
	Planned from theatre	152 (24.4)	592 (26.8)	744 (26.3)	
	Unplanned from theatre	28 (4.5)	50 (2.3)	78 (2.8)	
	Unplanned from ward	14 (2.2)	24 (1.1)	38 (1.3)	
	Missing (*n*)	10	33	43	
Total length of hospital stay	Days (up to 30 postoperative days), median (i.q.r.)	7.0 (5.0–11.0)	7.0 (5.0–10.0)	7.0 (5.0–11.0)	0.367
	Missing	0	0	0	
Anastomotic leak	No	561 (90.0)	2090 (94.7)	2651 (93.7)	<0.001
	Yes	62 (10.0)	117 (5.3)	179 (6.3)	
	Missing (*n*)	10	35	45	
Grade of the anastomotic leak	None	0 (0.0)	9 (7.6)	9 (5.0)	0.081
	Grade A*	10 (16.1)	27 (22.7)	37 (20.4)	
	Grade B*	12 (19.4)	21 (17.6)	33 (18.2)	
	Grade C*	40 (64.5)	62 (52.1)	102 (56.4)	
If yes: How was the leak diagnosed?	Clinical diagnosis only	13 (21.0)	19 (16.5)	32 (18.1)	0.490
	Ultrasonographic imaging	2 (3.2)	10 (8.7)	12 (6.8)	
	CT imaging	46 (74.2)	83 (72.2)	129 (72.9)	
	MRI	0 (0.0)	0 (0.0)	0 (0.0)	
	Intraoperative diagnosis	1 (1.6)	3 (2.6)	4 (2.3)	
Intra-abdominal or pelvic collection	No	541 (86.8)	2048 (92.9)	2589 (91.5)	<0.001
	Yes	82 (13.2)	157 (7.1)	239 (8.5)	
	Missing (*n*)	10	37	47	
Grade of intra-abdominal or pelvic collection	None	1 (1.2)	4 (2.5)	5 (2.1)	0.268
	Grade A*	27 (32.9)	64 (40.8)	91 (38.1)	
	Grade B*	17 (20.7)	38 (24.2)	55 (23.0)	
	Grade C*	37 (45.1)	51 (32.5)	88 (36.8)	
If yes: How was the collection diagnosed?	Clinical diagnosis only	10 (12.2)	15 (9.7)	25 (10.6)	0.529
	Ultrasonographic imaging	11 (13.4)	32 (20.8)	43 (18.2)	
	CT imaging	58 (70.7)	103 (66.9)	161 (68.2)	
	MRI	0 (0.0)	0 (0.0)	0 (0.0)	
	Intraoperative diagnosis	3 (3.7)	4 (2.6)	7 (3.0)	
Anastomotic leak or collection	No	521 (83.6)	2003 (90.8)	2524 (89.2)	<0.001
	Yes	102 (16.4)	204 (9.1)	306 (10.8)	
	Missing (*n*)	10	35	45	
Reoperation within 30 days	No	559 (89.7)	2054 (93.3)	2613 (92.5)	0.003
	Yes	64 (10.3)	147 (6.7)	211 (7.5)	
	Missing (*n*)	10	41	51	
Readmission within 30 days	No	566 (90.9)	2057 (93.5)	2623 (92.9)	0.032
	Yes	57 (9.1)	144 (6.5)	201 (7.1)	
	Missing (*n*)	10	41	51	
Mortality within 30 days	No	592 (95.0)	2137 (97.1)	2729 (96.6)	0.016
	Yes	31 (5.0)	64 (2.9)	95 (3.4)	
	Missing (*n*)	10	41	51	

Values are *n* (%) unless otherwise indicated. *Grade A—requiring no further intervention, grade B—requiring radiological reintervention, and grade C—requiring surgical reintervention.

The lower AL associated with safe-anastomosis training was also reflected by lower intra-abdominal collections. Intra-abdominal or pelvic collection occurred in 7.0% (EAGLE trained) and 13.0% (untrained) respectively (*Table S4*). The difference persisted across operative contexts with AL rates of 7.2% in the training group compared with 14.4%, and collections 12.1% *versus* 21.8%. When combined, total major postoperative intra-abdominal events (leak or collection) occurred in 9.1% of trained *versus* 16.4% of non-trained procedures (*Table S4* and *Fig. S3*).

### Secondary outcome measures

Completion of online EAGLE training, including the in-theatre checklist and the AL risk calculator (*Tables S5–S9*), was associated with a lower frequency of adverse secondary outcomes and also reflected the online training engagement rates (*Tables S7–S9*). Reoperation within 30 days occurred in 10.3% of patients in the non-trained group compared with 6.7% in the trained group, an effect that remained after adjustment (aOR 0.63 (95% c.i. 0.44 to 0.90)) (*Fig. 2*). Readmission within 30 days was lower, 6.5% compared to 9.1% (aOR 0.72 (95% c.i. 0.53 to 0.98)), while mortality at 30 days was 2.9% compared to 5.0% (aOR 0.58 (95% c.i. 0.36 to 0.93)). AL was associated with a 11.9% risk of 30-day mortality, whereas those without a leak had a 2.9% risk. The median length of stay was 1 day shorter among patients treated by trained surgeons, at 7 (i.q.r. 5–10) days compared with 8 (i.q.r. 5–11) days in the non-trained group (*P* = 0.041). AL risk was higher in a low- or middle-income setting; however, this is confounded by more emergency surgery being performed in low- and middle-income countries.

The proportion of stoma formation without anastomosis was higher in patients where the AL risk calculator was used *versus* when it was not used (86 of 771 (11.2%) *versus* 164 of 2102 (7.8%); *Table S8*) and where the in-theatre checklist was completed (83 of 687 (12.1%) *versus* not completed 167 of 2186 (7.6%)).

### Risk factors

Subspecialization was not identified as a risk factor for AL in this study population. General surgery residents did see a higher rate of AL (*Fig. 2* and *Table S7*), but this was for a small subgroup of patients. EAGLE training uptake was greater among consultant surgeons (2372) than resident surgeons (501), but a lower rate of AL was seen both for consultants and for trainees after EAGLE training (*Table 2*). No hospital imposed restrictions on surgeon participation, regarding whether they were a trainee or a consultant.

Independent predictors of AL included the urgency of surgery, ASA grade, and intraoperative contamination. Emergency operations carried significantly greater risk (aOR 1.67 (95% c.i. 1.22 to 2.28)), as did patients with ASA grade III–V (aOR 1.36 (95% c.i. 1.01 to 1.83)). The presence of a contaminated or dirty operative field conferred the highest risk (aOR 3.21 (95% c.i. 2.28 to 4.53)) (*Fig. 2* and *Table S4*). The large majority of the contaminated and dirty surgery was performed as an emergency (287 of 823 (34.9%)) and led to a substantial portion of the ALs (*Fig. S2*). By contrast, neither hospital income classification nor operative approach (open *versus* minimally invasive) retained independent significance after adjustment.

### Timing of EAGLE training

In hospitals that participated in EAGLE-1 and EAGLE-2, the surgeons undertook the initial training ≥1 year before participating in EAGLE-2 (1273 patients). This contrasts with hospitals that only participated in EAGLE-2, where the training was undertaken within weeks of participation in the study. Despite this gap (in EAGLE-1 hospitals) between training and undertaking the surgery, a lower AL was still evident and comparable to that seen in sites that only participated in EAGLE-2 (*Table S6*). When outcomes were examined across hospitals participating in EAGLE-1, EAGLE-2, or both, a sustained pattern of benefit from training was evident. Among centres new to EAGLE-2, the leak rate was 15.2% in the control group compared with 9.7% in the intervention group; in hospitals that had participated in both EAGLE-1 and EAGLE-2, leak rates also declined (from 11.5% in the control group to 8.4% in the intervention group).

## Discussion

This large, international cohort study demonstrates that surgeons worldwide will undertake virtual training outside a structured research protocol. Moreover, completion of the safe-anastomosis training by the operating surgeon is associated with a clinically meaningful and significantly lower rate of AL after right colectomy. The effect size was robust, with an AL rate of 16.1% among non-trained surgeons and 9.4% among those who completed the modules. Importantly, these results were observed in routine clinical practice across 332 hospitals in 60 countries^16,17^, of which 63% were hospitals that had not taken part in EAGLE-1. This was a function of the expanding influence of the ESCP Global Reach Committee that reached out to multiple national surgical societies in different countries and the increasing use and rapid spread of social media. These findings further confirm that Crew Resource Management (CRM) tools in surgery constitute an essential means of reducing complications. The high uptake among operating surgeons also suggests that brief online training can be implemented without major disruption to routine surgical workflows.

Beyond the lower AL, training was also associated with lower rates of reoperation, readmission, and mortality, as well as a shorter hospital stay. These outcomes have marked economic benefit, given the high costs associated with treating AL-related complications. The results are consistent across different operative settings and income classifications, emphasizing the scalability of this approach. Furthermore, the use of the in-theatre checklist and the preoperative leak risk calculator both reflected the rates of online training and were associated with fewer adverse outcomes. Though we did not collect granular data on duration spent by individual surgeons on the online training materials, nor test them on their learning, the adoption of the learning into clinical practice by way of the checklist and calculator indicates strong engagement by surgeons and direct translation into real-world settings. The results validate the findings from the EAGLE-1 RCT^7^ in a second patient population and outside a clinical trial setting. However, unlike EAGLE-1, the present study was observational and should be interpreted as supportive evidence of effectiveness rather than definitive causal proof.

The magnitude of benefit associated with safe-anastomosis training compares favourably with other interventions proposed to reduce AL, including intraoperative fluorescence angiography or reinforcement sutures^18–21^. Unlike these approaches, the training package requires no specialized equipment by leveraging behavioural optimization. The consistency across two large international studies strengthens the argument that structured digital education can change behaviour and improve outcomes^7^. This places the safe-anastomosis training on a par with similar interventions in surgery that have made a significant impact on clinical outcomes such as the WHO checklist, enhanced recovery after surgery programmes, and surgical-site infection bundles^22–25^. The success of these interventions is strongly associated with how well the interventions were implemented^26–28^.

Several mechanisms may explain the observed effect. Structured modules can highlight key intraoperative technical steps, such as ensuring adequate blood supply, avoiding tension, and reinforcing the anastomosis. Training also emphasized intraoperative leak recognition, prompting preventative corrective measures. The in-theatre checklist performed just before the anastomosis decision and formation then spotlights this key step in the operation to the entire theatre team, focusing everyone on the most hazardous operative step. There was also an increase in formation of stomas in those for whom the preoperative leak risk was calculated and the in-theatre checklist was completed. This is most likely a function of additional consideration of leak risks and formation of riskier anastomoses abandoned in lieu of stomas. Thus, this would correlate with fewer ALs in these groups. In addition, engaging surgeons may foster a culture of attentiveness and accountability, minimizing omissions in technique that predispose to leak.

EAGLE-2 has several strengths. It was prospectively conducted with an a priori analysis plan, it included a large and diverse international cohort, and it achieved high data completeness. The inclusion of hospitals across different income settings and operative environments increases the external validity of the findings. Outcomes were defined using internationally recognized standards, ensuring comparability with other series. The data indicate that the effect was reproducible and transferable. However, limitations must be acknowledged. As an observational study, residual confounding cannot be excluded, even after multivariable adjustment. Training was voluntary, raising the possibility that motivated surgeons were both more likely to complete modules and to deliver better outcomes, although these findings were mirrored in the EAGLE-1 RCT where confounders were mitigated by randomization. Training exposure is self-reported by the surgeon and not objectively verified, and there are no data on timing of training, depth of engagement, or partial completion. The overall AL rate was higher in EAGLE-2 than EAGLE-1. This effect is likely a consequence of EAGLE-1 being conducted across an interval that included the SARS-CoV-2 pandemic and thus captured patients’ outcomes when they were highly selected for operations. In contrast, EAGLE-2 is a representation of real-world outcomes and captured routine patient care well after the end of the pandemic restrictions.

EAGLE-2 has strong generalizability because it was delivered as a pragmatic, prospective, international cohort study across income settings, specialist colorectal units and general hospitals, and both elective and emergency practice. The inclusion of consecutive patients reflects real-world practice rather than a highly selected trial population.

Important questions remain. The impact of training on left-sided resections has not yet been established and will be addressed by planned follow-on audits. Future studies could explore the durability of knowledge gained, the potential role of refresher modules, and integration with video-based assessments of operative performance.

## Collaborators

See Appendix.

## Supplementary Material

znag107_Supplementary_Data

## Data Availability

Data sharing requests will be considered by the management group upon written request to the corresponding author.
