# Supplementary material for: Impact of safe-anastomosis digital training on 30-day anastomotic leak after right colectomy: international prospective cohort study (EAGLE-2)
Source: Br J Surg. 2026 Sep 11;113(9):znag107. doi: 10.1093/bjs/znag107 (PMC13564221; doi:10.1093/bjs/znag107)
Supplement: Supplementary file 1 [file znag107_supplementary_data.zip › EAGLE 2 Collaborator List - 12052026 - All Countries.docx]

**List of authors:**

**Writing Group**

Elizabeth Li*, Sue Blackwell, Pamela Buchwald, Laura Castro, Sanjay Chaudhri, Sharfuddin Chowdhury, Dragomir Dardanov, Audrius Dulskas, Muhammed Elhadi, Alaa El-Hussuna, Caterina Foppa, Matteo Frasson, Gaetano Gallo, James Glasbey, James Keatley, Michael Kelly, Beatriz Silva Mendes, Ana Minaya, Peter Neary, Ionut Negoi, Francesco Pata, Thomas Pinkney, Gabrielle van Ramshorst, Shaji Sebastian, Baljit Singh, Dion Morton**

*First and corresponding author. **Senior author and overall guarantor.

**Data analysis**

Omar Omar, Bryar Kadir, Elizabeth Li

**Management Team**

Dion Morton, James Keatley, Elizabeth Li, Sivesh Kamarajah, Eleanor Oliveira

**ESCP Global Reach Committee**

Dion Morton, Simon Ng, Nicolas Avellaneda, Erman Aytac, Romina Bianchi, Pamela Buchwald, Peter Christensen, James Keatley, Suk-Hwan Lee, Varut Lohsiriwat, Surendra Kumar Mantoo, Klaus Matzel, Luqman Mazlan, Frederic Ris, Jun Won Um, Carolynne Vaizey, Jaw Yuan Wang, Janindra Warusavitarne, Jun Watanabe, Sofia Xenaki, Hongwei Yao

**National Leads**

Nicolas Avelleneda (Argentina), Maria Bubenova (Austria), Helio Moreira Junior (Brazil), Martin Karamanliev (Bulgaria), Peter Vlcek (Czechia), Mark Bremholm Ellebæk (Denmark), Baljit Singh (United Kingdom), Mahmoud M. Elsayed (Egypt), George Tzovaras (Greece), Amrit Pipara (India), Marco Caricato, Gaetano Gallo, Francesco Pata (Italy), Paulius Žeromskas (Lithuania), April Roslani (Malaysia), Muhammed Elhadi (MENA region), Johanne Bloemen (Netherlands), Miguel Cunha (Portugal), Christina Fleming, Peter Neary (Ireland), Ionut Negoi (Romania), Mohammed Sbaih (Saudi Arabia), Ana Minaya Bravo (Spain), Jeremy Meyer (Switzerland), Pamela Buchwald (Sweden), Ibrahim Mutwakil (Sudan), Arda Isak, Sezai Leventoglu (Turkey), Andriy Beznosenko (Ukraine)

**Algeria**

Farid Allam, Farouk Bakhti, Ghania Rahmoune, Dounia Aktouf, Hamid Aliyahia (Etablissement Hospitalier Spécialisé SALIM ZEMIRLI)

**Argentina**

Agustina Zabani, Oscar Silva, Patricia Orsingher (Hospital "Dr. Alberto Balestrini"); Candelaria Beruti (0009-0005-9588-3946), Carina Chwat (0000-0002-2123-0388), Diego Valli, Flavia Alexandre, Gustavo Lemme, Lucas Peirano, Mauro Ramírez Duarte, (Hospital Universitario Austral); Antonela Di Gennaro (0009-0006-2945-9546), Claudia Carrizo (0000-0003-2314-0324), Fabio Leiro (0000-0002-9477-2997), Julieta Espino (0000-0002-5929-0773), Mauro Trama (0000-0001-5197-3185)(Penna Hospital); Federico Yazyi (0000-0002-2368-1158) Magali Muthular (0000-0001-6934-095X), Alexis Braggio (0009-0003-1164-2957), Diana Alejandra, Vasquez Velasquez (0009-0000-8712-8127), Leandro Stawkowy (0000-0002-9862-3083), Oriana Comodo (0009-0006-6511-4203) (Hospital General de Agudos Dr. Cosme Argerich); Dario Barrera (0009-0005-5873-4562), Nicolás Luis Avellaneda (0000-0002-6802-7125), Sonia Antonela La Rosa, Ariel Enrique Grinblat, Rocio Ailen Gau, Augusto Javier Carrie (Hospital Universitario CEMIC)

**Australia**

Chun Hin Angus Lee, Jason Cox, Anna Fiess, John Edington, Jasmine	Tan, Jessica Paytner, Melissa Phu (Bendigo Health); David Proud (0000-0003-3561-0725), Lyons Nick (0000-0003-3921-106X), Amanda Kerton, Ranjan Guha (0000-0002-6732-5606), Ian Ong (0000-0002-7347-9863), Thomas	Tiang (0000-0001-7702-3479)(Austin Hospital)

**Austria**

Dominik Weihs	, Kristina Wrodnigg, Claudia Wette, Dirk Bernard, Daniel Schmid, Gergely Kovacs (KH der Barmherzigen Brueder St Veit Glan); Maria Bubenova (0000-0002-3317-2285), Bernhard Beer (0009 0005 1944 3471), Karin Kreuzhuber. Jürgen Simharl (0009-0008-3061-4384), Stefan Stättner (0000-0002-5226-7597)( Salzkammergut Klinikum Vöcklabruck); Gerd Pressl, Thomas Saini, Verena Wieser, Matthias	Biebl, Clemens Rohregger, Wolfgang Zaglmair (Ordensklinikum Linz Elisabethinen); Felix Aigner (0000-0001-8931-736X), Zoltan Horvath, Markus Braumille, Geza Gemes (0000-0002-1881-8615), Nicole Koter (0009-0003-0443-1609), Gabriele Moitzi, Elisabeth Wallner (Barmherzige Brüder Graz); Iliyan Iliev (0000-0001-6377-2874), Michaela Layr (0009-0007-4016-3436), Ines Stipic, Bernhard	Dauser	(0000-0002-8324-6203)(St. John of God’s Hospital)

**Belarus**

Andrey	Litvin (0000-0002-9330-6513), Valentin Bereshchenko (0000-0001-8269-8075), Valentin Dashkevich (0000-0002-1926-7341), Aleksander Misevich (0000-0002-3577-8635)(Gomel Specialized Regional Clinical Hospital)

**Brazil**

João Fraga (0000-0001-9753-0128), Jeud Bravo (0009-0006-5885-2216), Marylin Da Silva (0009-0007-5086-7656), Zamir Fernando (0009-0004-6899-9129), Barbara Prais (0009-0006-5453-6865), Marcela Paula (0009-0006-2165-3205), Fábio Jabour (0000-0001-9733-0636)(Hospital Maternidade Therezinha de Jesus); Fabio Lopes de Queiroz (0000-0002-5061-9552), Daniel Mauricio, Londoño Estrada (0000-0002-2241-4509), Alexandre Sette,	Bruno lucena de souza Pires, Marco Antonio Miranda Dos Santos, Helida Aline Tomacheski Amaral, Antonio	Lacerda Filho (Felicio Rocho); Carlos Augusto Real Martinez (0000-0001-8088-427X),	Danilo Toshio Kanno (0000-0001-7120-4017), Patricia Krause Askinis (0009-0000-8067-0677), Matheus Gobbo Cordeiro (0009-0008-2102-8746), Fernando Augusto Lima Marson (0000-0003-4955-4234), Camila Vantini Capasso Palamim (0000-0001-6825-1154)(Hospital Universitário São Francisco de Assis na Providência de Deus); Roberto Kaiser Junior, Mikaell Faria, Ana Regina Bptista, Anderson Gomes (Kaiser Hospital Dia)

**Bulgaria**

Manol Sokolov (0000-0002-2608-333X), Angel	Arabadzhiev (0000-0003-2186-3799),	Dimitrin Tonova	(0000-0001-8724-4320), Gergana Ivanova (0000-0001-8464-0328), Svilen Maslyankov (0000-0002-9510-9943), Tsvetan Popov (0000-0002-5037-150X)(University Hospital Alexandrovska); Dobromir Dimitrov (0000-0003-3313-1093), Martin Karamanliev (0000-0001-5166-0752), Galya Pacheva (0009-0008-7776-7003), Slavina	Yohanova (0000-0002-4722-1295), Meri Shoshkova (0000-0002-1629-0204), Paulina Vladova (0000-0002-2200-8156), Preslav Vasilev	(0000-0001-6603-4893)(University Hospital Dr Georgi Stranski)

**China**

Xiaodong Wang (0000-0002-5617-3627), Jianbo Liu (0000-0002-0507-1508), Yue Wen (0009-0008-7567-5383), Fei Liu (0009-0007-9670-465X)(West China Hospital, Sichuan University)

**Czechia**

Zdeněk	Kala (0000-0003-4187-388X),	Martin	Svoboda (0000-0001-5607-3450), Lucie Fraňková, Deana	Slovjaková, Vladimír Procházka (0000-0001-7747-6072), Tomáš Grolich (0000-0001-5465-5879)(University Hospital, Brno); Petr Vlček (0000-0002-7344-9752), Beata Hemmelová, Denisa	Kliková, Miloš	Chobola, Tamara Vystrčilová, Alena Berková, Michal Reška (St. Anna's University Hospital); Zuzana Šerclová (0009-0001-6789-6847), Aneta	Tremerová (0009-0001-4529-065X), Martina Rumlová	(0009-0003-9064-3818), Iveta	Čechová (0009-0003-9162-5275), Petra Zusková (0009-0005-4444-9576), Jakub	Freml (0009-0008-9979-9989)(University Hospital Královské Vinohrady); Jaroslav	Kalvach (0000-0003-3541-9939), Kristýna Pončáková	(0009-0002-9040-377X), Michal Ondica (0009-0001-4489-8719), Jan Votava (0000-0001-7840-3262), Matúš Murín (0009-0002-6096-0065)(Surgery Department 2nd Faculty of Medicine Charles University and Central Military Hospital)

**Denmark**

Mark Bremholm Ellebaek (0000-0001-6550-7505); Jens Kristian Baelum (0000-0003-0135-2430),	Dorthe Kathrine Slot (0000-0001-8634-2164),	Søren Rune Larsen (0009-0008-8059-0472),	Lars Uldum Erlandsen	Claville (0009-0006-0329-5352), Pedja Cuk (0000-0003-0582-604X),	Rasmus Tofte Hansen (0009-0002-7213-0624)(Odense and Svendborg University Hospital); Aleksandar	Dzodan (0009-0002-5190-2242), Michael Tvilling Madsen (0000-0003-0652-1234), Kathrine Nagel Host (0009-0006-5991-5581), Abdullah Mostafa Al-Ansari (0009-0009-9638-5120)(Slagelse Hospital)

**Ecuador**

MIGUEL CORDERO MUÑOZ (0000-0002-6339-3871),	Alexander Rodriguez (0009-0006-7257-1891), Sheila Palma (0009-0001-1671-9111), Gabriela Torres (0009-0004-5999-4027)(HOSPITAL DE ESPECIALIDADES PORTOVIEJO

**Egypt**

Galal Abouelnagah (0000-0001-8017-7444), Mohamed Al Sayed (0000-0001-9361-7007), Engy Amgad	(0009-0000-5805-8377), Ahmed Ibrahim (0009-0008-0136-3010), Hashem Altabbaa (0000-0002-8031-838X), Nour Eldein Saad (0009-0009-7005-7049), Albaraa Daradkeh (0000-0002-8282-8236)(Alexandria Main University Hospital); Mohamed Zarad (0000-0001-8438-0386), Enas Mohamed (0009-0001-4300-3303), Ghaida Hamid (0009-0000-7135-5843), Guma Khalid (0009-0001-1999-1364); Abd Elrahim Mohamadin (0000-0002-6566-5578), Mohamed Magdy Shaapan (0009-0004-2787-2906), Marwa Adel Arafa Ebrahim (0009-0000-6043-9769), Amr Abd Ellatif Mostafa (0009-0007-5945-8541), Abdelrahman Elmorshdy (0009-0009-2521-7904), Alaa Ibrahim (0009-0005-6658-1746), Sara Elmorshdy (0009-0005-8138-0547)(Mataria Teaching Hospital); Hossam El-Fol (0009-0003-7362-8704), Mohamed Bassiony (0009-0000-6607-1715), Khalid Mohamed (0009-0006-6574-3093),Mostafa Mansour (0000-0003-1660-0476), Mohamed Elkhouly (0009-0008-7235-1690), Salma Kemaz (0009-0003-3014-5630),Hatem Amr Elzahaby (0000-0003-1719-625X)(Menofiya University Hospital); Mohamed Hefzy (0000-0003-1249-8662), Marwan Abdou (0000-0003-4196-9339), Ahmed Khedr (0000-0002-0056-8902), Ahmed Moussa (0009-0009-3642-0422), Aya Bessa (0009-0000-5429-6482), Mohamed Badawy (0009-0005-3728-8639), Mohammed Elalamy (0000-0002-0969-7081)(Alexandria Medical Research Institute); Mohamed Abdalsalam (0009-0007-2627-2038), Ibrahim Beshai (0000-0001-6101-4316), Aya Nassar (0009-0007-3486-6944), Mahmoud Abdelmodaber (0009-0001-9962-5574), Omnia Radwan (0009-0008-1569-2050), Ahmed Gomaa	(0000-0002-7026-4664), Salma Abdou (0009-0008-4062-3620)(Mansoura Health Insurance Hospital); Mahmoud M. Elsayed (0000-0001-9585-185X), Ahmed	Khalifa (0009-0004-5134-2139), Ahmed M. Elsayed (0009-0004-7692-7117), Nermen Farag (0009-0008-4423-8519),	Mohamed Sorour (0009-0008-4423-8519), Ahmed Nagah (0009-0009-0909-1272), Omar Hafez (0009-0005-0435-0803)(Cairo University Hospital); AhmedSaber Mohamed Abdelrahman (0000-0003-2049-6085), Sarah Mansour (0000-0002-6520-1423), Kariman Saber, Mostafa Farag (Giza International Hospital); Ahmed Hafez (0009-0002-9503-5240), Alaaeldin Esmail (0009-0002-9601-1400), Marwan Fakhry (0009-0009-2485-6261), Said Rashed (0009-0002-9503-5240), Yara Hani (0009-0003-8981-0340), Shahd Ramadan (0009-0008-5092-9260), Hasnaa Elshazly (0009-0001-3499-8003)(El-Salam Oncology Center); Hesham A.Elmeligy (0000-0002-1824-6602), Omar Younes (0009-0009-4154-8359), Samar Mohammed (0001-0001-7294-9256), Hend F. Hassan	(0000-0002-1469-0597	), Hazem Korani (0009-0009-4154-8359)(Theodor Bilharz Research Institute); Islam Metwally (0000-0002-5981-9614), Mohamed Elbanna (0000-0002-3199-8120), Khaled Abdelwahab, Mosab Shetiwy (0000-0003-4465-0980), Mohamed Abdelkhalek, Saleh Elbalka (Oncology Center Mansoura University); Sarah Magdy Abdelmohsen (0000-0001-5208-5307), Mohie El-Din Madany (0000-0001-9930-6779), Jamal Hussein (0000-0001-6639-0599), Tarek Hemaida (0000-0003-0255-6339)(Aswan University Hospital); Aya Ziada (0009-0001-0448-1580), Mahmoud M.	Elsayed (0000-0001-9585-185X), Bushra Qabala (0009-0002-0817-4032), Hussein Hussein (0009-0005-2822-1993), Khalid Sarhan (0009-0005-5474-4755), Mohamed Sherif Ali Ahmed (0009-0007-7258-039X), Rashad G. Mohamed (0009-0004-2962-0075)(Mansoura Specialized Hospital); Ibrahim Saleh Alawadi (0009-0003-7040-2566), Mohamed W Omar (0000-0002-6610-8554), Ahmed Hewadi (0009-0005-0611-7917), Sherif Wael	(0009-0009-9900-0820), Youstina Mohsen (0000-0002-5949-1794),	Mohammed Yasser Abdelfattah (0009-0004-6711-1159), Abdulrahman Hesham Khairy (0009-0005-7012-808X)(Mansoura University Hospital – team 1); Ayman Taher Shemes (0000-0002-6824-7865), Hisham Hazem Warda (0000-0003-2016-5518), Mostafa Ismail Mahmoud Hassan (0009-0001-0148-4639), Mohamed Salama (0000-0003-3734-4889), Mohamed Attia Elfadali (0000-0002-2532-0831), Yara Sherif Elsayed (0009-0006-6417-6824), Mohamed Sherif Ali (0009-0007-7258-039X)(Mansoura University Hospital – team 2); Ahmed Shehta (0000-0002-9184-8597), Amr Sanad	(0000-0003-4545-2556), Aziza	El-Saey (0009-0006-1006-4608), Moataz Emara (0000-0001-8911-1812), Mohamed Sherif (0009-0007-7258-039X), Yara Sherif (0009-0006-6417-6824), Mostafa Ismail (0009-0001-0148-4639)(Gastrointestinal Surgery Center, Mansoura); Mohamed Naieem Abdelaal (0000-0001-9267-1116), Mohamed	Talaat Abdelhalim (0009-0004-2687-1442), Mahmoud Rabie (0009-0002-5066-7850), Karim Khattab (0009-0004-2650-4888), Ahmed Abo Saif (0009-0002-1706-8450), Mohamed	Nazeef	(0009-0004-1915-0129)(Tanta University Hospital); Heba Taher (0000-0001-7654-5670), Mujtaba Zakria (0000-0002-8761-9281), Walaa Mohamed Kamel (0000-0003-1331-6277), Sara Saleh (0009-0003-4082-299X), Moamen Hassan (0009-0000-1193-9468), Abobakr Saleh (0000-0002-6336-5694), Noureldin M. Sayed (0009-0007-1267-9072)(Kasr Alainy Hospital); Ahmed Y Azzam (0000-0002-4256-0159), Osman Elamin (0000-0002-7845-2192), Omar S Elsayed (0009-0000-4635-6545), Mahmoud M. Morsy (0009-0001-4356-5306), Adam Elswedy (0009-0008-0154-0507)(Elsafa Hospital); Amr Kassem (0000-0002-6540-8889), Ahmed Ibrahim (0009-0007-8278-381X), Ahmed Ahmed (0009-0007-8278-381X), Ahmed Ebrahim (0009-0007-8278-381X)(Egyptian Liver Hospital); Emad Mostafa (0009-0007-4124-5740), Omar Zayed (0000-0002-0614-6575), Abdrahman Ramadan (0009-0001-5077-9812), Manar Zein (0009-0009-9597-1806), Youssef Eid (0009-0003-0760-7139), Karim Elhusseini (0009-0003-7262-180X), Mohamed Hamed (0009-0009-7292-7220)(October 6 University Hospital); Ahmed Abostate (0000-0002-1405-438X), Ahmed Reda	(0009-0002-0143-4209), Eslam Radwan (0009-0002-6211-871X), Ahmed Mohsen (0009-0001-7678-9111), Omar Saeed	(0009-0001-6247-424X), Abdelrahman Saeed (0009-0007-5079-9462), Mahmoud Ramadan (0009-0000-6191-2685)(Banha University Hospital)

**Ethiopia**

Fitsum	Terefe (0000-0003-3716-6795)(Yekatit 12 Hospital Medical College)

**Germany**

Benjamin Muessle (0009-0005-9783-0485), Jan-Philipp Ruff (0009-0009-3460-7875), Annett Rauscher, Stephan Möller (0009-0008-1564-1771), Nuh Rahbari (0000-0002-4703-9039), Marko Kornmann (0000-0002-9533-8711), Emrullah Birgin (0000-0002-0338-3727)(University Hospital Ulm); Ulf Kulik, Alexander Wagner, Winny Andrea, Hendrik Eismann, Christel	Koczur, Regine Pfeiffer (Medizinische Hochschule Hannover); Steffen Seyfried (0000-0003-3239-6727), Maike Hermann, Lena Lamm, Valentin Walter, Julia Hardt (0000-0003-0997-3776), Christoph Reißfelder (0000-0003-4591-1046)(Mannheim University Medical Center (Universitätsmedizin Mannheim)); Frieder Pullig (0000-0002-8499-0931), Robby Langbecker (0009-0003-0308-2873)

**Ghana**

Emmanuel Kafui Ayodeji (0000-0001-9698-1920)(Pentecost Hospital, Madina)

**Greece**

Spiros Delis (0000-0001-9498-7763), Evgenia Charitaki (0000-0002-5375-0097), Alexia Nduska, Nikos Pentilas	(0000-0002-5381-9517), Dimitrios Karakaxas (0009-0005-8109-6298)(Konstantopouleio General Hospital of Athens); George Theodoropoulos (0000-0003-4900-9711), Maximos	Frountzas (0000-0001-9157-9860), Petros Georrgitsogiannakos, Eleni Pagalou, Vasileios Kostoulas, Despoina Kanata (Hippocratio General Hospital); Konstantinos Stamou (0000-0002-8302-0319), Sotirios Sterpis (0000-0001-6285-1490), Eleni Kerasioti (0009-0000-4873-6250), Georgios Retzios (0009-0007-3795-3357)(MITERA Hospital); Vasileios Papadopoulos	(0000-0002-1009-1685	), Paraskevi Chatzikomnitsa (0000-0001-7879-0426), Savvas	Mavromatidis (0000-0002-6435-8349), Dimitris Bliamplias (0000-0003-1326-7022), Dimitrios Giakoustidis	(0000-0002-6023-4744), Alexandros Giakoustidis (0000-0002-3786-4609),	Gregory Christodoulidis (0000-0003-3413-0666)(Papageorgiou General Hospital); Francesk Mulita (0000-0001-7198-2628), Levan Tchabshvili (0000-0001-5312-630X),	Eleni Bekou (0009-0002-1290-1802), Admir	Mulita	(0009-0003-0331-5008), Elias	Liolis (0000-0001-7863-6402)(General Hospital of Eastern Achaia - Unit of Aigio); George Tzovaras (0000-0001-5322-8269), Ioannis Baloyiannis (0000-0002-6050-3000), Chamaidi	Sarakatsianou	(0000-0002-9509-581X), Eleni Arnaoutoglou	(0000-0003-0441-9442), Konstantinos Perivoliotis (0000-0002-6622-5734), Ioannis Mamaloudis (0000-0002-2630-6185), Effrosyni Bompou (0000-0002-2887-0978)(General University Hospital of Larissa); Elissaios	Kontis	(0000-0002-2619-1058), Ioannis	Katsaros (0000-0002-6743-8942), Athina Oikonomou (0009-0005-7977-8556	), Christos Maniatis (0000-0003-0482-1458), Charalampos Theocharopoulos (0009-0007-6725-2228)(Metaxa Cancer Hospital); Athanasios Syllaios (0000-0002-5053-0953), Zoi Gkiafi (0009-0007-8335-3889), Irena Myrtaj	(0000-0002-7763-9385), Asimina Valsamaki (0000-0001-8814-0371),	Antonios Koutras (0000-0002-7229-9983), Stavros P.	Papadakos (0000-0003-1583-1125),	Alexandros Pergaris (0000-0002-4960-672X)(Lamia General Hospital); Thalia Petropoulou (0000-0001-5486-5905),	Artemis Liapi (0009-0008-1044-3901),	Lounta	Bouznic, Aris Bakas (Athens Euroclinic); Dimitrios Schizas (0000-0002-7046-0112), Nikolaos	Machairas (0000-0003-3239-3905), Adam Mylonakis (0000-0002-0272-3259), Panagiota Stratigopoulou (0000-0002-5240-7844), Stylianos Kykalos (0000-0002-8687-685X), Myrto D.	Keramida (0000-0003-1834-7443), Panagiotis	Sakarellos (0000-0001-6427-8798)(Laiko University Hospital); Ioannis	Papaconstantinou (0000-0002-4614-9041), Leonidas	Chardalias (0000-0002-3079-6180), Eirini Kalamara	(0000-0001-7171-3620), Kassiani Theodoraki	(0000-0003-4890-4642), Theodosios Theodosopoulos	(0000-0001-5570-402X), Manousos Konstadoulakis (0000-0002-9999-5022), Dimitrios	Politis (0000-0001-8520-2071)(Aretaieion Hospital); Manousos Christodoulakis, Andreas Strehle (0000-0001-6781-9596), Sofia Papantonaki (0000-0003-2883-7518)(Creta InterClinic); Orestis Ioannidis (0000-0002-0861-8783), Elissavet	Anestiadou (0000-0001-5650-7319), Despoina Fragkiadaki (0009-0009-2101-5993), Ioanna Dimitropoulou (0009-0003-0587-3740), Konstantinos Siozos, Ourania Kerasidou (0009-0008-7958-8017),	Aliki Brenta

**Hong Kong**

Kaori Futaba (0000-0002-7358-5439),	Nicole Miu Yee	Cheng	(0000-0002-1137-330X), Wing Kin Tse (0000-0003-4559-0684), Vivian Nga Man Lau (0000-0003-1612-4780)(Prince of Wales Hospital)

**Hungary**

Balazs	Banky (0000-0002-4297-9072), Tímea	Drexler	(0009-0007-1948-5289), Ágnes Kis, János Széll (0000-0002-8493-0971),	Mátyás	Hamar	(0000-0003-0145-3442), András Fülöp (0000-0003-1510-960X)(1st Department of Surgery - Semmelweis University)

**India**

Ankur Garg (0000-0002-6649-2701),	Rana M	Rajneesh (0009-0000-6838-2939), Pankaj Kumar, Sandeep Kumar (0000-0003-2445-6785)(Ram Manohar Lohia Hospital); Satish Subbiah Nagaraj (0000-0003-2363-982X), Naveen Pentakota (0000-0002-3872-6208),	Arun Chandran	, Karan Singla (0000-0002-5877-678X), Cherring Tandup (0000-0003-0230-7949), Yashwant Raj Sakaraj (0000-0003-1095-7810), Lileswar Kaman (0000-0003-1837-2614)(Postgraduate Institute of Medical Education & Research, Chandigarh); Amit Gupta (0000-0002-4847-4120), Ammapalem Satish (0009-0004-9342-4837), Umesh Kumar Sedwal (0009-0000-4471-7594), Praveen Talawar (0000-0002-9931-2316), Deepak Rajput (0000-0001-9630-2932), Rahul Kumar, Ritu Thakur (All India Institute Of Medical Sciences, Rishikesh); Poorvikha S (0000-0002-8205-1148),	Vinutha Gowda	(0009-0009-3684-8509), Chandrakanta Gowda (0009-0009-1481-3962), Savitri Maridevegowda (0009-0003-8455-7032)(St John's Medical College Hospital); Tushar Subhadarshan	Mishra	(0000-0001-9215-1647), Soumyajit Jana (0009-0006-3239-1853), Babu Lal Jakhar (0009-0001-3567-6647), Neha Singh (0000-0003-2013-4295), Dillip Kumar Muduly (0000-0001-6427-5714), Bramhadatta	Pattnaik (0000-0003-2461-1001), Monika Gureh (0000-0002-1580-6037)(All India Institute Of Medical Sciences, Bhubhaneshwar); Pradeep	Saxena (0000-0002-7838-9752), Siddharth Kumar Sinha (0009-0004-4603-7287), Morpal Meena (0009-0009-4181-6210), Vaishali Waindeskar (0000-0002-0556-7159), Ajeet Pratap	Maurya	(0000-0002-5845-7236)(All India Institute Of Medical Sciences, Bhopal); Diwakar Pandey (0000-0002-0090-0530), Tushar N Suryawanshi (0009-0007-8432-1942), Rahul Singh (0009-0008-1457-0609), Hiten Mistry (0009-0000-7059-7517)(Sanjeevani CBCC USA Cancer Hospital); Nivesh Agarwal (0000-0002-8477-3574), Subham Jakhar (0000-0002-1989-3175), Deepak Kumar (0000-0002-6120-8863), Gunjan Chaudhary	(0000-0002-0630-748X), Mohinder Kumar Garg (0000-0003-3299-2908), Utkarsh Kumar (0000-0003-4324-418X)(Kalpana Chawla Government Medical College); Lovenish Bains (0000-0002-8627-0452), Mohamed	Mustaqeem, Sourabh Das, Lalit Gupta (Maulana Azad Medical College); Rahul Gupta (0000-0001-7461-2083), Deepak Gusain	(0009-0006-6255-5548), Laxmi Goyal, Rahul Varshney (0000-0001-7468-399X)(Synergy Institute of Medical Sciences); Ashwani Kumar (0000-0003-8217-6573), Manisha Aggarwall (0000-0001-9655-1381), Parth	Dhamija (0000-0002-7678-7140), Lalit	Garg (0000-0002-0109-9466), Ashwani Kumar (Government Medical College Patiala); Amrit Pipara (0000-0002-0782-4980), Moameren (0009-0001-4031-0893), Huirongbam Madon Singh (0000-0002-6360-4261), Neha Desai (0000-0002-2163-928X)(Tata Medical Center); Shiv Rajan (0000-0003-2513-6281), Vijay Kumar (0000-0001-6477-8274), Devanshi Verma (0000-0002-4605-0674), Tanmay Tiwari (0000-0001-6234-5010), Naseem Akhtar (0000-0001-8724-3248), Mohit Singh (0000-0002-0660-4011), Abhilasha Tripathi (0000-0002-6072-5252)(King George's Medical University); Sharada Sundaramurthy (0000-0002-8167-4861), Suvam Kumar (0000-0003-2765-4939), Eloni K (0009-0009-2048-3445), Samzai Lungalang	(0009-0002-6383-6510), Handilu Rengma (0000-0002-6727-5901), Durgesh Ommi (0000-0002-7808-9223)(Christian Institute of Health Sciences and Research); Bharath S Veerabhadrappa (0000-0003-4645-6655), Badareesh L (0000-0002-0762-0427), Prema Keerthi Shetty (0009-0000-9838-8899), Rama Rani K (0000-0001-6291-8544), Stanley	Mathew (0000-0002-3207-1378)(Kasturba Medical College Hospital), Rauf Wani (0000-0003-1182-7003), Fazl Parrey (0000-0003-3657-7808),	Gowhar Aziz (0000-0002-3416-5765),	Showkat Gurcoo (0000-0001-7873-3552)(Sher-i-Kashmir Institute of Medical Sciences); L. V. Gouri, Mahadev Prasad Patra, Prativa Das, Arum Kumar Sahoo (Pandit Raghunath Murmu Medical College and Hospital); Haris Chandra Dash, Jyotiranjan Mohapatra, Rojalin Parida, Santosh Kumar Roy (Life Institute of Gastroenterology & Gynaecology); CHANDRASHEKAR MAHAKALKAR	(0000-0002-9969-9725), Shivani Kshirsagar (0000-0003-2565-9509), Arun Teja Karusala (0009-0009-2644-7445), Akaksha Yachmanani (0000-0002-9344-4406)(Acharya Vinoba Bhave Rural Hospital)

**Iraq**

Arwa S. Alabide (0009-0001-2700-4111), Ahmed Qasim Mohammed Alhatemi, Esraa S. Alabide, Hashim Talib Hashim (Al-Nassiryah Teaching Hospital); Maytham A. Al-Juaifari (0000-0002-8106-9354), Najah R Hadi (0000-0002-8415-5311), Mohammad Hussein Abd Munaf	(0009-0009-0744-7650), Ghadeer Mohammed Abbas (0009-0000-4555-9869), Eathar Haidar Aljubori (0009-0004-3838-9661)(Al-Sader Medical City); Ahmed Sermed Al Sakini (0009-0006-7740-1453),	Sandra	Thair Al-Aish (0009-0008-1667-644X),	Ali Al-Shammari (0000-0002-4298-5456), Anne Sermed Al Sakini (0009-0005-1780-283X), Aya Saad (0009-0001-2303-2916)(Baghdad Medical City)

**Ireland**

Peter Neary, Haroon Ur Rashid (0000-0003-4174-7576), Collette Flynn, Vida	Hamilton (University Hospital Waterford/University College Cork); Michael Kelly (0000-0002-0757-6411), Maeve O'Neill, Ben Creavin, Brian Mehigan, John Larkin, Paul	McCormick (Trinity St James Cancer Institute)

**Italy**

Francesco Autuori, Francesco	Madeddu, Marco Nieco, Sabrina Pilloni (CTO); Gaetano Luglio (0000-0002-5931-9599), Michele Cricrì (0000-0003-2359-1504), Salvatore d'Angelo, Martina D'Aniello, Francesca Paola Tropeano, Antonio	Miele (Federico II University of Naples – team 1); Giovanni Aprea (0000-0003-2487-1006), Marianna Capuano (0000-0002-7623-7747), Salvatore D'Angelo, Giuseppe De Simone, Giuseppe Palomba (0000-0003-3954-5166),	Raffaele Basile (Federico II University of Naples – team 2); Alberto Aiolfi (0000-0002-7764-6075), Francesco Cammarata (0000-0002-4055-8485), Federica Grasso (0009-0001-8732-1514), Davide Gottardello (0009-0007-6677-9802), Michele	Manara (0000-0002-4924-1919), Davide Bona (0000-0003-4501-1187)(Istituto Clinico Sant'Ambrogio); Giuseppe Sena (0000-0001-9793-3250), Giorgio Ammerata, Marco Scozzafava, Marco	Pignataro (University 'Magna Graecia' of Catanzaro); Diego Sasia, Ribero Dario, Bonardello Michela, Palmisano Sarah, Lucia Mir Mennea (0009-0001-2812-9805), Chiara Montini (0009-0005-4228-5635), Giacomo Bottero (0009-0006-7781-8814)(Santa Croce e Carle Hospital); Laura Fortuna (0000-0003-0624-7445), Francesco Coratti	(0000-0001-5980-7333), Fabio	Staderini (0000-0002-2438-2563), Gabriele Baldini (0009-0004-6342-9696)(Azienda Ospedaliera Universitaria Careggi); Giuseppe Sica	(0000-0002-7407-0584), Giulia Bagaglini	(0000-0002-0185-9103)(Policlinico Tor Vergata Hospital); Francesco Ferrara	(0000-0002-8208-1945	), Rosaria Lo Bianco (Department of Surgical, Oncological and Oral Sciences. University of Palermo); Mauro Podda (0000-0001-9941-0883),	Eleonora Locci (0000-0001-5309-2138), Chiara Curcio (0009-0003-5680-1640), Salvatore Sardo (0000-0001-7664-8928), Adolfo Pisanu (0000-0002-6212-9753), Valentina Murzi (0000-0002-4021-2273), Paola Marongiu (0000-0001-9025-129X)(Cagliari University Hospital); Nicoletta Sveva Pipitone Federico (0000-0002-1697-2846), Roberta Tutino (0000-0002-0891-4689), Michela Forgione (0009-0009-6229-9216), Gelsomina di Ciero (0009-0007-4935-3877), Mauro	Santarelli (0000-0001-7984-2240), Francesca Sbuelz (0009-0005-3981-8365)(Città della Salute e della Scienza); Bruno Scotto (0000-0001-9998-9645), Francesca Ascari (0000-0002-9959-0922), Angela Marzella, Giuseppina Giulino, Antonia Lavinia	Zuliani	(0000-0003-2136-9694), Ubaldo Marra (0009-0003-1319-2718	), Carolina Bobbio (Bernardino Ramazzini); Roberto Santoro, Cristina Carruezzo (0000-0001-9565-2439), Riccardo Ciucciarelli (0009-0006-0737-1076), Guido Turi (0009-0003-2311-9530), Laura Antolino (0000-0002-9559-3595), Manuela Brighi (0009-0009-7906-3920), Domenico Giannotti (0000-0003-0295-0811)(Ospedale Belcolle - ASL Viterbo); Andrea	Bottari	(0000-0003-2784-5954), Gianmario Poto (0000-0001-8515-2414), Elisabetta Panduri, Emilio Giorgio Guerra	, Francesco Renzi (0000-0001-6568-3461)(Valdarno Hospital); Alessandro Puzziello, Elio Donnarumma (0000-0001-6126-3873), Francesco	Roca (0009-0003-4370-4550), Antonio Romanelli (0000-0002-6895-6485), Giovanni Fabbrocile (0009-0000-7731-1542), Alessandro Liguori (0009-0007-7611-2534)(AOU Ruggi); Silvia	Palmisano (0000-0003-4749-4258), Manuela Mastronardi (0000-0003-3525-3692), Beatrice	Modesti, Lucia	Comuzzi, Nicolò	de Manzini (0000-0002-8362-9044), Biagio Casagranda, Edoardo Osenda (Cattinara University Hospital); Valeria Tonini (0000-0003-3130-2928), Lodovico	Sartarelli (0000-0003-1625-9239), Wladimiro Siciliano (0000-0002-0269-2018), Angelo Antonio Ciccarese	(0000-0001-9822-531X), Claudia Mongelli (0000-0001-8459-3103), Yuri Macchitella (0009-0007-9242-9961)(Santissima Annunziata); Andrea Barina	(0000-0003-2295-7367), Giacomo Ghio, Aurora Iris Giacomello (0009-0009-3415-2452), Edoardo Segato, Carola Cenzi (0000-0002-6000-3444)(IOV - Istituto Oncologico Veneto); Nicola Passuello (0000-0002-8259-1064), Alice Pecorino (0009-0003-3432-9808), Federico Soldan (0009-0002-0694-8428),	Alessandro De Cassai	(0000-0002-9773-1832)(Azienda Ospedaliera di Padova); Nirvana Maroni, Caterina Froiio, Silvia Sala, Andrea Galimberti, Andrea Pisani Ceretti (ASST Nord Milano, Ospedale Bassini); Daniela Rega	(0000-0002-2342-9390), Ernesto De Giulio (0000-0001-7027-2640), Pasqualina Perrotta (0009-0002-7880-6883), Francesca Bifulco (0000-0003-1116-3502), Paolo Delrio (0000-0002-4271-4939), Carmela Cervone (0000-0001-8271-0070), Silvia de Franciscis (0000-0002-0353-4759)( Colorectal Surgical Oncology Unit - Istituto Nazionale Tumori Fondazione, Pascale-I.R.C.C.S); Gennaro Martines (0000-0001-9669-9079), Giovanni Tomasicchio (0000-0001-7017-2690),	Pellegrini Alessandro, Pappagallo Leonardo (University of Bari 'Aldo Moro'); Andrea Muratore (0000-0002-8049-6549), Marcello	Calabrò, Cristina Valentino (0000-0000-0000-0000), Alberto Modini (0000-0000-0000-0000), Giulia Marchiori (0000-0000-0000-0000), Sofia	Gamba	(0000-0000-0000-0000), Patrizia Marsanic (Edoardo Agnelli); Ciferri	Enrico (0000-0002-4873-0489), Longhin Roberta, Epis Lorenzo (0000-0001-7591-9335),	Emiliana Parodi, Barberis Andrea (0000-0003-0472-0120), Rossi Lisa	(0000-0002-7341-3194)(Galliera Hospital); Antonio Amato (0000-0001-9566-7008), Paola Batistotti (0000-0001-7439-6325), Vitantonio Cirillo (0009-0006-6930-4987), Tiziana D'amato (0009-0008-2761-6063), Davide	Cuneo	(0000-0002-0790-3355)(Ospedale di Imperia); Ruggero Bollino (0000-0002-5585-1262), Helen Yu (0009-0009-5195-4916), Carla De Carlo, Andrea Viani	(0009-0007-3795-4958)(Azienda Unità Sanitaria Locale - IRCCS di Reggio Emilia); Giuseppe Evola (0000-0002-3648-7063), Ettore Gula (0009-0000-0336-123X), Carla Di Stefano (0009-0004-2691-3251), Marco Vacante	(0000-0002-6815-5012), Luigi Piazza (0000-0002-6768-6972), Andrea Leonardi (0009-0006-2588-7711), Salvatore Lo Bianco (0000-0002-0161-0535)(ARNAS Garibaldi); Gaya Spolverato (0000-0002-7275-2573), Francesco Celotto (0000-0002-1881-0605)(University of Padova); Andrea Mingoli (0000-0001-5437-9085), Pierfrancesco Lapolla (0000-0002-3088-5263), Giuseppina Carta (0000-0002-8062-4651), Francesco	Pugliese (0000-0002-8076-1250), Gioia Brachini (0000-0001-6724-5588), (Bruno Cirillo (0000-0003-1889-2644), Simona Meneghini (0000-0002-0287-8517)(Policlinico Umberto I); Marco Milone (0000-0001-5612-1926),	Anna D'Amore	(0000-0003-1264-4295	), Salvatore D'Angelo (0000-0003-0991-1807), Carmine Lacovazzo (0000-0002-5032-1668), Pietro Anoldo (0000-0001-6727-2973),	Michele Manigrasso (0000-0001-8204-7942), Giovanni Domenico De Palma (0000-0001-7474-7180)(Federico II University of Naples – team 3); Luigi	Zorcolo (0000-0002-4086-9144), Simona Deidda	(0000-0002-7756-9727), Giulia Murgia	, Francesca Piras, Angelo Restivo (0000-0001-9165-818X), Valerio Argiolas (Cagliari University Hospital – team 2); Ugo Grossi (0000-0001-5372-2873), Patrizia Pelizzo (0000-0002-7839-0761), Massimo De Paoli (0000-0003-3099-4010), Paolo Zanatta (0000-0002-9522-3690), Giulio Aniello	Santoro (0000-0002-0086-3522), Eleonora Mollica	(0009-0006-3517-0888), Marco Piccino (0000-0002-2902-5143)(Regional Hospital ULSS 2 Marca Trevigiana); Giovanni Gambino (0000-0003-0400-5178), Rizzo Giovanna, Bertino Vanessa , Moncada Valentina (UOC General and Oncological Surgery AOOR Villa Sofia-Cervello); Michele De Capua (0000-0001-6392-3388),	Jacopo	Andreuccetti (ASST Spedali Civili, Ospedale di Brescia); Alessio	Giordano (0000-0002-9505-791X), Davina Perini (0000-0003-0967-6798), Pasquale	Punzo, Lorenzo Foti, Francesca Cammelli (0000-0001-5149-0888), Maximilian Scheiterle (0000-0001-6522-9891), Jacopo Martellucci (0000-0002-7437-9098)(Azienda Ospedaliera Universitaria Careggi – team 2); Carcato Marco, Gabriella Teresa, Capolupo, Alessandra	Inzeo, Renato Ricciardi, Filippo Carannante, Gianluca Costa, Valentina Miacci (Campus Biomedico, Rome); Vincenzo Lizzi (0000-0001-9387-9314), Nicola Tartaglia (0000-0001-6845-7080), Anna Maria Cairelli (0009-0006-4683-2357),	Antonella Cotoia (0000-0002-0154-0418), Alessandra Giuliani	(0000-0001-8712-8637), Giovanna Pavone (0000-0003-2523-4914), Mario Pacilli (0000-0002-1473-6962)(Ospedali Riuniti Azienda Ospedaliera Universitaria); Matteo Viti, Valentina Rampulla (0000-0001-7386-1969), Antonella Bosco, Zoltan Ghenkis Lance Eric Varga (0009-0005-8590-3739), Marco Mariani (0000-0002-0813-069X)(Asst bg Ovest); Federica Ferracci (0000-0002-8040-2231),	Ilaria Tersigni (0009-0003-2617-9627), Giusy Fortunato (0000-0003-3171-7971), Fabio Pacelli (0000-0002-2013-6525)(Fondazione Policlinico Universitario Agostino Gemelli – team 1); Claudio Fiorillo (0000-0001-7681-3567), Lodovica Langellotti (0009-0007-9571-8718)(Fondazione Policlinico Universitario Agostino Gemelli – team 2); Roberto Persiani (0000-0001-5584-6095), Alberto	Biondi (0000-0002-2470-7858), Anna Paola Sanesi (0000-0003-0907-2310), Paola Aceto (0000-0002-0228-0603), Laura Lorenzon (0000-0001-6736-0383), Roberto Pezzuto (0000-0003-0585-3769),	Flavio Tirelli (0000-0001-6160-5992)(Fondazione Policlinico Universitario Agostino Gemelli – team 3); Francesco Quaglino (0000-0002-4661-7511), Federico Festa (0000-0001-7685-588X), Salome Pfannkuche (0009-0009-1767-7441), Antonella Evangelista (0009-0002-5711-9865), Luca Cestino (0000-0003-3119-8606), Alex Bruno Bellocchia (0000-0002-4815-4856), Beatrice Degan (0009-0001-2851-0169)(ASL Città di Torino - Ospedale Maria Vittoria); Guglielmo Clarizia	(0000-0003-0592-2904); Alessandro Longhini (0000-0002-4293-0754); Federica Giossi, Veronica Raveglia (0009-0001-0460-6574); Alessandro Spolini (0000-0001-8534-3528)(Ospedale di Sondrio (ASST Valtellina e Alto Lario); Emanuele Asti	(0000-0002-2485-8269), Pamela Milito (0000-0002-5926-3943), Tiziana Nania (0000-0002-9982-9147),	Luca Di Girolamo (0000-0002-8047-5545), Daniele Tiziano Bernard (0000-0002-1109-7974), Stefano Siboni (0000-0003-0532-3135), Marco Sozzi (0000-0003-1153-123X)(U.O. di Chirurgia Generale e d'Urgenza, IRCCS Policlinico San Donato); Massimiliano	Di Paola, Chiara De Lucia, Barbara Proietto, Eugenia Puleo, Luca Giordano, Gherardo Romeo, Nunzio Maria Angelo Rinzivillo (San Pietro Fatebenefratelli); Luigi Masoni	(0000-0001-9866-4224), Matteo Cinquepalmi	(0000-0003-3267-7481), Alessandra Pastone (0009-0000-1670-8781), Luca Ruggeri (0009-0004-0748-1086), Federico Maggi (0009-0002-4103-3979), Giulia Lauteri (0009-0003-9349-335X), Giovanni Guglielmo Laracca (0000-0002-4508-2180)(Ospedale San Carlo di Nancy GVM); Silvio Guerriero, Zuleyka Bianchi (0009-0000-7153-8121), Chiara Di Clemente	, Silvia Di Rosa	(0009-0000-7153-8121)(Augusto Murri); Raffaele Galleano (0000-0001-9787-4908), Elena Ronda (0009-0007-8526-7677), Emanuela Massa	(0009-0009-1472-0061), Paolo	Minuto	(0009-0005-3504-9394), Omar Ghazouani (0000-0003-1075-0342), Bianca Angelone (0009-0003-8011-7830)(San Paolo); Marco De Prizio (0000-0003-0872-9229), Lorenzo Maria Fatucchi (0000-0001-6106-5519), Michela Boncompagni (0009-0007-8178-997X), Sara Alunno (0009-0008-3925-0759), Riccardo Malatesti (0000-0001-8384-9728), Alessio Mazzoni (000-0001-7768-3805), Rezart Sulce (0000-0003-2025-035X)(Ospedale San Donato USL Toscana Sud Est); Camillo Leonardo Bertoglio (0000-0002-5925-983X), Lorenza Zampino	(0000-0003-1985-0091), Paola	Lampugnani, Giancarlo Razionale, Antonella Nisi (0000-0002-3787-2326), Martina Pardo	(0009-0004-6901-1194)(Ospedale Giuseppe Fornaroli); Edoardo	Saladino (0000-0003-0521-959X), Giuseppe Cuticone (0009-0006-1713-8788), Elisa Bertilone (0009-0009-1658-1624), Orazio Bonanno (0009-0003-5534-4679)(Azienda Ospedaliera Papardo); Maurizio Ronconi (0000-0002-8491-1703), Silvia Casiraghi (0000-0003-1160-8946	), Magda Manfredini (Gardone Val Trompia Hospital); Fabio Carbone (0000-0003-4743-7181), Roberto Santalucia (0000-0001-8275-3150), Anna Platto (0009-0008-5429-336X), Amerigo Galla, Simona Borin (0000-0001-7463-723X), Marco Realis Luc	(0009-0006-9024-5061)(Istituto Europeo di Oncologia - IRCCS -Milano); Mariano Fortunato	Armellino, Francesco Guida (0009-0003-1820-8010), Sara di Matteo, Serena Testa, Roberta Abete (0000-0001-8770-7946), Gianpaolo Marte (0000-0003-3965-9542), Andrea Tufo (0000-0002-4495-1241)(Ospedale del mare, Naples); Diletta Cassini (0000-0003-1118-9817), Chiara Limongi (0009-0008-5236-1736), Elisabetta Confalonieri, Stefano Clementi, Francesca De Stefano (0009-0004-1580-3487)(Ospedale "Città di Sesto San Giovanni"); Vincenzo Tondolo, Gianluca Rizzo, Luca Emanuele Amodio (Ospedale Isola Tiberina Gemelli Isola); Stefano Zaccone, Dario Sarli (0009-0004-1597-2037), Giovanna Cosetta, Filippo Alberghini, Andrea Pierre Luzzi (0000-0003-4218-3848), Giovanni Spiezio (0000-0002-3164-690X), Marina Pighin (0009-0001-3438-3340)(San Carlo Borromeo); Stefano Amore Bonapasta (0000-0002-4091-0880), Ilaria Peluso, Viola Tordi, Emiliao Mantovani, Matteo Gregori, Simone Santoni, Marco Venza (Policlinico Casilino); Stefania Angela Piccioni (0000-0002-9050-4127), Ludovico	Carbone (0000-0003-2862-6788), Elena Boffa (0009-0006-3202-042X), Franco Roviello (Azienda Ospedaliero Universitaria Senese); Francesco Fleres (0000-0002-1092-8975), Carmelo Mazzeo (0000-0001-9893-7480), Serena Recupero	(0009-0003-9019-2906), Vincenzo F Tripodi (0000-0002-1661-6171), Teresa Sinicropi	(0009-0000-1684-1849), Santino A Biondo (0000-0002-4720-3345), Eugenio Cucinotta (0000-0002-2749-3971)(Policlinico Universitario G. Martino of Messina); Mario Annecchiarico (0000-0002-9760-9386);	Giulio Argenio (0000-0001-9187-9003); Antonio Varricchio (0000-0002-1255-5666); Claudia Cirillo (0009-0005-8936-3565); Francesco Carafa (0000-0002-4311-0838)(AORN San Pio); Nicola Cillara (0000-0001-6877-2267), Antonello Deserra (0000-0002-2950-4670), Maria Provenzano (0000-0002-5034-1650),	Nicola Scalas (0000-0002-4808-8625), Francesca D'Agostino (0000-0001-7632-4480)(Santissima Trinità - ATS Sardegna); Angela Pezzolla (0000-0001-7810-8239), Annunziata Panebianco	, Lulia Lucarelli, Angela	Di Ciaula, Ferdinando Massimiliano Anelli, Mara Sblendorio (Azienda Ospedaliero Universitaria Consorziale Policlinico Di Bari – team 1); Mario Testini (0000-0002-9297-4894), Giovanna Di Meo (0000-0002-7012-8654);Arianna Pontrelli (0009-0002-4306-8104, Lucia Ilaria Sgaramella (0000-0002-6087-4119), Angela Gurrado (0000-0002-2009-5831), Francesco Paolo Prete	(0000-0003-0426-8752), Alessandro Pasculli (0000-0003-2276-2383)(Azienda Ospedaliero Universitaria Consorziale Policlinico Di Bari – team 2); Norma Depalma (0000-0001-5451-2923), William Sergi (0000-0002-4232-5589), Federica Cagnazzo, Nicolas Adam (0000-0002-0933-7705), Marcello Giuseppe Spampinato	(0000-0003-4036-6819), Stefano D'ugo (0000-0002-7073-0565), Claudia De Giorgi (P.O.'Vito Fazzi'); Christian Cotsoglou (0000-0002-6348-0027), Beatrice Torre (0000-0002-7388-3241), Loredana Monina, Francesco Curto (ASST Vimercate); Massimiliano Veroux (0000-0002-2780-6421), Roberta Granata (0000-0003-1857-0805), Ludovica	Alessandro (0009-0009-2767-3196), Luigi La Via	(0000-0002-2156-7554), Giordana Riccioli (0000-0001-8311-2267),	Marianna Scribano (0009-0008-1592-0535); Costanza Distefano (0000-0003-3163-6864)(Azienda Ospedaliero- Universitaria Policlinico San Marco); Matteo Rottoli (0000-0003-0278-4139), Alice Gori	(0000-0002-7780-0618), Gianni Di Croce, Andrea Zanoni, Giacomo Calini (0000-0002-7460-9578), Stefano Cardelli (0000-0001-8870-5176), Nicola Greco (IRCCS Azienda Ospedaliero-Universitaria di Bologna); Guido Mantovani (0000-0002-4590-325X), Marianna Cippini, Federica BenazziI, Antonio Francipane, Luigi Boccia (0000-0002-1490-2879), Aaanlisa Pascariello (0000-0003-1184-1844)( ASST Mantua); Alberto Porcu (0000-0001-6307-8938), Teresa Perra (0000-0001-7032-1289), Vincenzo Pazzola (0000-0001-8868-4205), Alessandra Grazia Giuseppina	Garau (0000-0002-1967-9979), Silvia Oggianu	(0000-0002-0392-6365), Nicolò Canzoneri (0009-0001-1769-6169), Francesca Sassu (0009-0000-1555-4741)(Azienda Ospedaliero Universitaria di Sassari); Marco Palucci (0009-0009-8393-6667), Celeste Del Basso (0000-0003-3606-9512), Gabriele Viticchié (0009-0008-7343-2628), Mirco Leo (0009-0000-8512-5280), Igor Monsellato (0000-0003-3096-7901)(Azienda Ospedaliero-Universitaria SS. Antonio e Biagio e Cesare Arrigo); Gianluca Buzzi, Simone Targa, Barbara Mantovan, Mara Olga Bernasconi, Andrea Sanna (Santa Maria degli Angeli Hospital ULSS5 – Adria); Andrea	Rizzi, Simone Gianazza (0000-0001-9270-1709), Sonia Costabeber, Luca Botta, Luca Broggi, Marco Corbella, Mariacarla Besozzi (Ospedale L Galmarini); Dario Scala (0000-0002-7634-258X), Valerio Sciascia, Stefania Gregorio, Crescenza Guida (AORN S. Anna & S. Sebastiano); Francesco Bianco (0000-0003-4139-685X), Alessandra Novi (0000-0002-5159-078X);	Rosario Esposito, Mario Salvatelli, Antonio Cappiello (0000-0003-1293-2932), Simona Gili (0000-0001-5499-2349), Ersilia Corrado (0009-0008-9813-6948)(Ospedale S. Leonardo - ASL Napoli 3 sud, Castellammare di Stabia); Antonio	Zanghi (0000-0002-7765-9626), Andrea Cavallaro (0000-0002-0311-5191), Mariangela Giummarresi (0009-0003-1769-9399), Luigi La Via (0000-0002-2156-7554), Alessandro Cappellani (0000-0002-9113-2396), Simone Di Majo (Azienda Ospedaliero- Universitaria Policlinico San Marco – team 2); Massimiliano Mistrangelo (0000-0001-8506-6794), Francesca Farnesi (0000-0002-8001-954X),	Francesca Gioia, Gerardo Cortese (0000-0002-1323-6341), Elisabetta Seno (0009-0002-8769-9485), Marco Ettore Allaix (0000-0001-6206-6644), Mario	Morino	(0000-0003-4540-1409)(Città della Salute e della Scienza – team 2)

**Japan**

Akihiko	Horiguchi, Takahiro Tashiro (0009-0005-9691-9541),Hiroshi Komekura, Hiroyuki Kato (0000-0002-5798-2725), Takayuki Ochi, Toki Kawai (Fujita Health University Bantane Hospital)

**Jordan**

Omar Said Mansour (0000-0002-1824-9635), Yazan Alnairah (0009-0001-0042-7739), Orwa Aladaileh (0009-0000-8048-7071), Yaman Al Ghunimat (0009-0004-4643-6579), Younes Shatat (AL Hussein New Salt Hospital); Fadi Maaita (0009-0002-8724-1044),	Mohammad Debian (0009-0003-5649-5639), Tamer Almoumani (0009-0005-7249-8754), Sadeel Alrawashdeh (0009-0008-7725-3272)( Jordanian Royal Medical Services); M S El Muhtaseb (0000-0002-4190-3797), Lana Al-Sabe (0000-0003-3880-0938)(Jordan University Hospital); Bourhan Alrayes (0000-0002-0463-2270), Sief-Addeen Al-Tahayneh (0000-0003-3137-3080), Anas Alhjooj (0009-0003-5858-7860), Ahmad	Alsamnah (0009-0006-0561-3043), Suhel Batarseh (0009-0001-8308-9834), Yanof Al-Naggar (0009-0007-2501-7860), Mohammad Salah (0000-0003-2158-0967)(Islamic Hospital, Jordan)

**Libya**

Ayoub Akwaisah (0000-0002-4446-2747), Ahmed Ahmayda (0000-0003-1869-0292), Arwi Omar Kara (0003-2424-8940), Alhassan Ahmed Kashbour (0009-0009-1982-7706),	Alhussen Ahmed Kashbour (0009-0001-3316-4269), Faraj Abdurabbah	Faraj (0009-0002-9760-0784), Mohammed Younus Alabeedi (0000-0001-8373-4760)(Benghazi Medical Center – team 1); Mohamed Moftah Shanana, Amal Emran Khalleefah Alwirfili (0009-0008-8804-9365), Wanesa Abraheem, Abduljalel Abdulkarim Alzwai, Amira Hamad, Narjis Husayn, Asraa Alwirfili, Abtisam Alharam, Nagat Milad (Benghazi Medical Center – team 2); Fatimah Alzahra Abduljawad, Hibah Bileid Bakeer	(0000-0002-7456-6344), Farah Alshaeri, Malak Alkuwafe, Hawa Beheih, Eman Alkubti, Sharefa Al Teerah (Gharyan Central Hospital); Hibah Bileid Bakeer (0000-0002-7456-6344); Haitam	Shames (0000-0001-9846-5364), Aya Mohammed Alqaarh (0000-0001-9325-6598), Hashim Aborkhis (0009-0008-9286-4987), Haitam Shames (0000-0001-9846-5364), Akram Alkaseek (0000-0002-7915-1826), Laila Shalabi (0000-0001-7334-9711)(Gharyan Central Hospital – team 2); Sarah Aldressi (0000-0002-0462-0122), Mohammed Aziz (0000_0004_0393_9879), Hussin Boaza (0009_0002_3824_6447), Hakim Ibrahim (0009_0001_4158_2540), Naji Elshelmani (0009-0006-5640-9034)(Dar Alshifa Hospital); Mohamed Alsori (0000-0002-4097-2053), Reem Ghmagh (0000-0002-4012-5938), Rawia Jamal Ghmagh (0000-0002-3622-0430), Abdulqudus Deeknah, Eman Abdulwahed	, Entisar Alshareea (Tripoli Central Hospital); Yamen Mohammed, Monia	Ramadan (0009-0002-2559-7344), Ghazalah Masoud, Haeba Khteab, Antisar Almahdi Aeqaab, Marwa Saleh, Najwa Swidek Hamed (Tobruk Medical Center); Ahmed Abdurrahman Said Algeblawi (0009-0004-4321-628X)(Abuslim Trauma Hospital); Adnan Shareif (0009-0009-7927-9588), Mostafa Shafeik (0009-0000-7988-9858), Laila Aoheda (0009-0009-4089-2959), Talat Abu Salem (0009-0002-5546-8623), Mohamed Abed	(0009-0009-3705-4671), Mohamed Basha (0009-0006-1997-0623), Mahmoud Alorani (0009-0003-1443-3800)(Metiga Hospital); Saifaleslam Elsahli (0000-0002-1543-9628), Ezzeddin Elfagih (0009-0000-5507-1031), Amal Abdelaziz (0009-0004-9377-5821), Malak Baie (0009-0003-8130-438X)( Al-Helal, Misurata); Hajer Abusnina (0000-0002-5120-5415), Abdulrahman Yousf Jaber (0009-0006-7913-6315), Svitlana Cheshkivska (0009-0008-8155-8378), Bashir Albakosh (0000-0003-1031-8269), Majdolin Almahjoub	(0000-0002-3674-3485), Najat Ben Hasan (0000-0003-2590-5060), Marway Iqreewi (0009-0005-1658-9424)(Zliten Medical Center)

**Lithuania**

Saulius	Svagzdys (0000-0002-1742-6214), Henrikas Pauzas (0000-0003-1740-0281),	Rima Budrikienė, Andrius Macas (0000-0002-4048-3065), Tadas Latkauskas (0000-0001-7641-2421), Paulius	Lizdenis (0000-0002-4836-9240), Justas Zilinskas (0000-0003-1108-6420)(Lithuanian University of Health Sciences Kaunas Clinics)

**Malaysia**

Mohd Syakir Mohd Azahar (0000-0001-9849-8801), Muhammad Anwar Azrin	(0000-0003-2838-1098), Nurul Azmeera	Zamali,	Collins	Chong (0000-0002-4275-7373), Hizami Amin Tai (0000-0002-8930-673X), Nik Qisti Fathi (0000-0001-9217-9105), Mohd Hady Shukri	Abdul Satar (0000-0003-2966-580X)(Hospital Pengajar Universiti Putra Malaysia (HPUPM)); April Camilla Roslani (0000-0003-2458-965X), Sui Weng Wong (0000-0003-3373-189X), Norhayati	Mohd Hashim (0000-0003-4267-9173), Pui San Loh (0000-0002-3819-1602), Nora Abdul Aziz (0000-0003-3286-3794), Mohamed Rezal Abdul Aziz (0000-0003-2751-8662), Ruben Gregory Xavier (0000-0002-1677-7389)(University Malaya Medical Centre); Sanjay Dev Singh (0000-0002-3459-9360), Mohamad Akmal Aizat	Bakhori, Arbayah Laluroh, Pok	Xiang Yan, Mohammad Izwan Isa (Hospital Lahad Datu); Jothinathan Muniandy	(0000-0002-4519-166X	), Koh Chee Keong (0009-0000-7231-2597), Marcus Gabriel Pitchai Joseph (0009-0009-8771-1000), Nanthini Ganapathy (0009-0002-6668-3775), Chetawati Binti Abu (0009-0008-0747-2189)(Hospital Melaka); Andee Dzulkarnaen Zakaria (0000-0002-4826-9725),	Ong Xin Zen (0009-0002-7356-802X), Kamal Ariff Isa (0009-0000-4792-6116), Mohd Zulfakar Mazlan (0000-0002-3452-1280), Zaidi Zakaria (0000-0003-1644-3546), Wan Mokhzani Wan Mokhter (0000-0002-5622-0307), Mohd Nizam Md Hashim (0000-0002-4066-6558)(Hospital Universiti Sains Malaysia); Nabil Mohammad Azmi (0009-0002-6129-3786), Zairul Azwan Mohd Azman, Nur Afdzillah Abdul Rahman, Ismail Sagap, Soma Balaganapthi, Diana Melissa Dualim (Department of Surgery, Hospital Canselor Tuanku Muhriz, Faculty of Medicine, Universiti Kebangsaan Malaysia)

**Malta**

Josephine Psaila (0000-0001-6531-6514), Sarah Bowman (0000-0002-2737-5414), Marianne Debono (0000-0001-8992-379X), Anne-Marie Camilleri-Podesta (0000-0002-7863-2038), Predrag Andrejevic (0000-0002-4888-3012), Charles Cini (0000-0002-4619-2090), Matthew Sammut (0000-0002-1758-2663)(Mater Dei Hospital)

**Mexico**

Oscar Posadas Trujillo	(0000-0003-0731-7636), Danilo Tueme de la Peña (0000-0001-9520-3633), Diana Estrada Gutiérrez (0009-0007-1854-577X), Diana Díaz Arizmendi (0000-0002-5738-0193),	Noel Salgado Nesme (0000-0002-3559-5070), Oscar Santes Jasso (0000-0002-6393-4607), Alberto Nájera Saldaña	(0009-0002-3470-4834)(Instituto Nacional de Ciencias Médicas y Nutrición 'Salvador Zubirán'); Francisco Emmanuel Alvarez-Bautista (0000-0003-2645-832X), Mario Trejo-Avila (0000-0001-9249-3321), Erika Hernández-Montiel (0009-0007-0892-2164), Jorge M. Antolinez-Motta (0000-0002-3588-9781), Alejandra Nuñez-Venzor (0000-0003-0857-5629), Roberto	Punin-Neira (0009-0003-5510-7366), Asya Zubillaga-Mares (0000-0002-0838-5123)(Hospital General Dr. Manuel Gea González); Jesus Alonso Valenzuela Perez (0000-0003-3549-8219), Juan Carlos Cardozo Aguilar (0009-0005-5241-8913), Patricia Esquivel de Alba (0009-0007-3904-1335), Edson Roberto Zendejas Avelar (0009-0007-2917-5233), Emmanuel Guadalupe Gutierrez Lopez (0009-0007-8874-4969), Jaime Alejandro	Florian Lopez (0009-0004-3376-9446), Javier Luna Garcia (0009-0004-4547-010X )(Hospital Civil Dr. Juan I Menchaca); Carlos M. Nuño-Guzmán (0000-0003-1179-745X), Luis Bravo-Cuéllar	(0000-0003-2891-8561), María del Rosario Rojas-Martínez (0009-0003-1585-0489), Elvia Rocío Flores-Fonseca (0000-0001-9883-1619)(Antiguo Hospital Civil de Guadalajara); Alejandro González-Ojeda (0000-0003-2935-8703), Sergio Vázquez-Sánchez (0009-0007-4109-3695), Kathia Morfin-Meza, Evelia Romo Ascencio, Clotilde Fuentes-Orozco (0000-0001-6230-8359), Luis Suarez Carreón, Edgar Cortes Torres (Hospital de Especialidades, CMNO-IMSS)

**Netherlands**

Joop Konsten (0000-0002-8803-1914),	Maarten van Heinsbergen (0000-0002-8092-6713), Danique Smets (0009-0000-1320-3753), Sjef Schlooz (VieCuri Medisch Centrum)

**Nigeria**

Muhammad Daniyan (0000-0002-1615-6881), George Duke Mukoro (0000-0002-7085-965X), Joy Uwanamodo (0009-0001-6540-0270), Idris Mohammad (0000-0002-1152-186X), Bashir Mohammed (0000-0002-5845-5820), Stephen Gana (0000-0002-7249-5423), Linus Ukwubile (0000-0002-3183-3473)(Ahmadu Bello University Teaching Hospital); Jerry Gofrey Makama (0000-0001-7316-7344), Bashiru Yeshua Aminu (0000-0002-0424-4489), Samaila Joshua (0000-0003-2822-2599), Ifeanyi Kene	Aghadi	(0000-0002-8599-8169), Stephen Akau Kache (0000-0003-1374-5776), Joseph Dekah (0009-0007-1231-8066), Damai Chinyio (0000-0002-7970-8380)(Barau Dikko Teaching Hospital); Michael Aghahowa (0000-0001-6101-9360), Kayode Olofin (0009-0008-9915-6828), Eunice Olokun (0009-0002-0828-9948), Rosemary Nwokorie (0009-0007-0777-9749), Bata Gali (0000-0003-1752-0064), Muslimat	Alada (Asokoro District Hospital); Okechukwu Hyginus Ekwunife (0000-0002-5500-6042), Benedict Mmuodumogu (0009-0004-3993-5951), Chidiogo Ezennwa (0009-0005-7353-8260),	Cyril Nwachukwu (0000-0002-6463-6177), Ochonma Egwuonwu (0000-0001-5978-6392), Chiemelu Emegoakor (0000-0003-0037-8735), Kenneth Ugwuanyi (0000-0003-2327-3995)(Nnamdi Azikiwe University Teaching Hospital)

**Pakistan**

Ahmed Siddique Ammar (0000-0001-9728-7287)(Chaudhry Muhammad Akram Teaching and Research Hospital); Ahmad Faraz (0009-0009-1478-0953), Muhammad Faisal Khan (0009-0003-5951-5658), Farhat Nazneen (0009-0002-1877-412X),	Sayed Shah Husnain (0009-0009-1295-6337), Muhammad Attaullah Khan (0009-0003-5176-9039), Ayaz Gul	(0009-0004-8635-253X), Ihtisham ul Huq (0009-0007-7587-3702)(Lady Reading Hospital); Syed Ali Naqi Zaidi	Zaidi (0000-0001-5691-5291), Ihsan	Baloch	(0000-0001-8880-9231), Adnan Ali Memon (0009-0002-9721-3258), Adeel ur Rehman	(0009-0004-5983-1973	), Ghina Awais (0000-0001-8077-752X), Ghina Shamsi (0000-0003-1438-9451), Nazish Iqbal (0009-0004-5877-3353)(The Indus Hospital)

**Panama**

Reinaldo Isaacs Beron	(0000-0002-3539-9985), Mariela Hurtado (0000-0002-8216-0651), Yazmin	Arauz (0000-0002-9895-3883), Renata Castaño (0000-0002-2497-0613)(Hospital Regional Rafael Hernandez CSS)

**Peru**

César Huaroto Landeo (0000-0003-0419-9080), Juan Carlos Luna Cydejko (0000-0002-0826-6589), Maria Amalia Espinoza Huamán (0009-0008-4980-695X), Carolina Picasso Arias (0009-0006-6745-5984)(Clinica Internacional, Lima)

**Poland**

Wiktor	Krawczyk (0000-0001-8514-4035), Joanna Kosciolek (0009-0005-3963-9076), Genowefa Greif (0000-0002-4563-0036), Aleksandra Pluta (0000-0001-7384-3832), Jakub Wantulok (0009-0001-8649-224X), Andrzej Organ (0009-0009-6370-0024)(Wojewódzki Szpital Specjalistyczny w Sosnowcu); Michał Nycz (0000-0001-6941-9561), Karolina Snopek-Miśta (0009-0001-6589-3482), Monika	Rozkoszny, Ewa Musioł	, Mariusz Kryj (0000-0001-6269-7809),	Dariusz Waniczek (0000-0002-0410-8604), Paweł Musialski (Uniwersyteckie Centrum Kliniczne im. prof. K. Gibińskiego ŚUM w Katowicach); Jakub Migoń	(0000-0002-4294-0768), Michał Bąk (0000-0002-5978-153X), Adrianna Pielech (0000-0003-4270-7541), Wiktoria Kulińska (0009-0005-8521-0916), Jan Nicikowski (0000-0001-6563-2963)(Karol Marcinkowski University Hospital)

**Portugal**

Manuel Rosete	(0000-0002-0312-9825), Rita Andrade	(0000-0003-3650-0640), Leandro Ferreira, Ana Luisa Almeida (0000-0002-5998-8856), Catarina	Lopes (0000-0002-8662-2315), Inês Prior (0000-0002-4444-3597), Tiago	Antunes (0000-0003-0847-2929)(Centro Hospitalar e Universitário de Coimbra); Beatriz Caldeira (0000-0003-2232-9766), Inês Matias (0000-0002-5064-968X), Cristina Ribeiro (0000-0003-1293-5295),	Mariana Silva (0009-0007-1601-6110), Manuel	Cotovio (0000-0001-9120-7012), Joana Bolota (0000-0002-8319-8141), Sofia Leandro (0000-0001-7501-1597)(Hospital do Espirito Santo); Vânia Fernandes (0000-0002-2534-8500), Marta Martins (0000-0003-0017-9533), Carla Rebelo (0009-0001-3359-7179), Nuno Ribeiro (0000-0002-2355-7552), Sílvia	Silva (0000-0003-0080-9902),	Álvaro Gonçalves (0000-0002-4018-2457), Inês Romero (0009-0000-9317-1973)(Centro Hospitalar do Tamega e Sousa); André Caiado	(0000-0002-0356-8326), João Maciel, Sandra Paranhos, Ana Gaspa (Instituto Português de Oncologia de Lisboa Francisco Gentil); Diogo Gomes (0000-0001-9792-1399), Pedro Custódio, Maria José Duarte, José Guerreiro, Rodrigo	Nemésio, Isabel Paixao, Jorge	Balaia (Centro Hospitalar Universitário Lisboa Central); Pedro Santos (0000-0002-6022-4132), Paula Magro (0000-0003-4277-690X), Célia Carvalho (0009-0000-7313-4905), Francisco Neves (0009-0001-0291-5605), João Martins (0009-0005-6969-2177), Bernardo Patrício (0000-0002-7978-9299), Teresa Carvalho (0000-0002-1029-1222)(Hospital de Torres Vedras - Centro Hospitalar do Oeste); Hélder	Além (0000-0002-9338-4044),	Sofia Reis (0000-0002-5598-6538), Ana Carvalho (0009-0006-3157-5466), Alda Martins (0009-0001-8496-5664), Sara Patrocínio (0000-0003-3178-9545), Janine Carmelino	(0009-0000-1668-7335), Frederico Nazareth (0000-0003-2113-9717)(Centro Hospitalar Barreiro Montijo, EPE); Filipe	Ribeiro	(0009-0004-4374-1463), Ana Moreira	(0000-0002-0692-0591), Margarida Ventura (0009-0001-0897-8535),	Catarina Bastos (0000-0001-9646-9986), Maria Costa (0000-0002-8208-1689), Maria Reigota	(0009-0003-3510-917X), Maria Carvalho (0009-0003-5525-7348)(Centro Hospitalar do Baixo Vouga); Sofia Boligo (0000-0002-7327-821X), Gonçalo Figueirôa, Letícia Ribeiro, Daniela Zuzarte (Hospital Sao Francisco Xavier); Guilherme Fialho (0000-0001-9353-7066), Hugo Capote (0000-0002-6932-739X), Hermínia Telo (0009-0005-4202-1007),	Júlio Soares (0009-0009-3510-5834), Cristina Costa (0000-0001-7524-1253),	Sara Morais (0000-0002-8472-6591), Tamiris Mogne (0000-0002-6107-9037)(Hospital Doutor José Maria Grande); Sara	Brás, Ana Catarina Pinho, Vanessa Sousa, Ricardo Dias (Hospital das Forças Armadas); Sofia Pina (0000-0002-7139-9659), Beatriz Mendes (0000-0002-1978-4138), Ana Sanches (0000-0001-5063-5272), Gonçalo Mesquita (0009-0002-3981-2279), Juan Rachadell (0000-0003-1768-7497), Edgar Amorim (0000-0002-5405-4405), Miguel Cunha (0000-0002-7353-2425)(Centro Hospitalar Universitario do Algarve - Unidade de Portimão); Andreia Ferreira (0000-0003-4748-2513), Jéssica Ricardo (0000-0002-6040-5021), Maria João Martins (0000-0002-9824-1747), Nuno Matias (0000-0003-2163-8567), Mariana Claro (0000-0002-9593-2606), Alberto Silva (0000-0002-1170-6960)(Unidade Local de Saúde do Litoral Alentejano); Ana Nunes Vieira (0000-0001-7436-8500), Ana Cláudia Soares (0000-0001-7048-5929), Alexandra Terra (0009-0000-4354-7047), Ana Rita Pinheiro (0009-0001-1990-8196), Diogo Galvão (0000-0003-4728-9705), Débora Melo (0000-0002-1149-5809), António Mora (0000-0003-2304-1932)(Hospital de Santo Espirito da ilha Terceira); Sonia Fortuna Martins (0000-0001-5902-526X),Helena Devesa (0000-0003-4427-5068), Susana Pepino (0000-0002-5421-0842), Alberto Roxo (0000-0001-9783-8800), Sonia Marques (0000-0002-6457-0454), Renato Barradas (0000-0002-0287-0558)(ULS Lezíria); João Nobre (0009-0003-3109-3078), Patrícia	Bárbara (0009-0002-3306-6893), Inês	Damásio (0009-0007-8694-406X), Cátia Domingues (0000-0002-0701-4574), Inês Sousa (0000-0003-1345-4581), Maria Inês	Coelho (0000-0002-9045-924X)(Centro Hospitalar de Leiria, E.P.E.); Fernando Melo (0009-0005-9069-0766), Sara Andrade (0000-0002-4521-6812),	Natércia Santos (0009-0009-7007-8843), Ana Lopes (0009-0008-7803-6878), Daniela Pais (0000-0002-5495-9239),	Inês Colaço (0000-0001-7626-7762), Margarida Luís (0000-0002-4269-2717)(Hospital Distrital da Figueira da Foz); Pedro Caldes	(0000-0002-4344-3392), João Andrade (0009-0005-1603-4197), Paula Nobre	(0009-0005-7758-3894), Ana Rita Saraiva (0000-0002-9815-2074), Maria Adelaide Campos	(0009-0000-9011-7322), Sónia Bispo	(0009-0006-9146-9841), Maria João Vale (0000-0003-1386-1371)(Unidade Local de Saúde Da Guarda); Horacio Perez (0009-0006-1705-5325), Joana Peliteiro (0000-0003-3160-5590), Marina Ângelo (0009-0006-0522-0741), Alexandra	Amaral (0009-0001-3057-7420), Rui Rainho (0009-0001-6692-2265), Vanessa Bettencourt (0000-0003-0262-5955), Tatiana Neves (0000-0001-5086-6927)(Unidade local de Saude de Castelo Branco (ULSCB)); Carlos Serra	(0000-0003-3320-3464), Bernardo Maria (0000-0002-9814-4787), Beatriz	Silva (0009-0009-6117-1245), João Barbosa (Hospital do SAMS); Pedro Febra	(0000-0002-4943-6396), Rita	Galama (0000-0002-9219-4388), Carla Pires (0009-0007-1063-4612), Sérgio Faria Baptista (0000-0001-8497-7609),	Carlos	Bôto (0000-0001-6626-4023),	Maria Inês Seixo (0000-0002-9347-6942)(Centro Hospitalar Médio Tejo); Urânia Fernandes, Carolina Marques, Ana Isabel Rodrigues, Anabela Santos, Rita	Marques, Ricardo Vaz-Pereira. Daniela Martins (Centro Hospitalar de Trás-os-Montes e Alto Douro, E.P.E)

**Romania**

Nicolae Bacalbasa (0000-0001-8465-5079), Irina Balescu (0000-0001-6611-798X), Maria Mandroc (0009-0008-0474-1857), Cristina Martac (0009-0003-6849-8212)(Fundeni Clinical Institute); Octav Ginghina (0000-0003-1594-4357), Andrei Bogdan Vacarasu (0000-0003-3968-3227), Ruxandra Pop, Sorela Radoi, Marius Zamfir (0000-0002-1731-6066), Mara Mardare (0000-0002-5176-2772), Irina Bondoc (0000-0003-2364-5736)(Oncological Institute of Bucharest "Prof. Dr. Al. Trestioreanu")

**Russia**

Yuriy Voronin (0000-0003-1948-5506),	Kazimagomed	Zubailov (0009-0001-5477-8657), Marina Alexandrovna, Alexandr Butiaikin (0009-0001-1268-7626)(Surgut District Clinical Hospital); Petr Tsarkov	(0000-0002-7134-6821), Vladimir Balaban (0000-0002-7226-4641), Petr Tsugylya (0000-0002-0416-9300), Sergey Barkhatov (0000-0003-4702-5558),	Inna Tulina (0000-0002-6404-389X), Valery Nekoval (0000-0002-3192-3786)(Clinic of Coloproctology and Minimally Invasive Surgery, Sechenov Medical State University); Sergei Katorkin (0000-0001-7473-6692), Leonid Lichman (0000-0002-4817-3360), Vasilisa Kuper (0009-0006-8143-651X), Tatiana Shnitman (0000-0002-5743-4577), Pavel	Andreev (0000-0002-0264-7305), Olga	Davydova (0000-0001-7473-6692),Andrei Zhuravlev (0000-0002-0353-840X)(Hospital Surgery Clinic of Samara State Medical University); Aleksei Karachun	(0000-0001-6641-7229), Aleksandra Olkina (0000-0002-1811-6226), Marina Smolina, Sergey Rosengard, Evgeniy Zagaynov (0000-0003-2024-7425), Gleb Samsonov (0009-0002-4998-1762)(N.N. Petrov National Medical Research Center of Oncology); Evgeniy Drozdov (0000-0003-4157-9744)(Siberian State Medical University)

**Singapore**

Ker-Kan Tan (0000-0002-0410-0450), Kai Yin Lee, Siew Ching	Wong, Ne-Hooi Will Loh (0000-0002-4114-1286), Chermaine Ang (National University Hospital); Neng Wei Wong (0000-0003-0028-6128), Jin Yang Crystal Sia (0009-0006-6858-4163), Hehe Shang (0009-0002-4938-0764), Alex Joseph (0000-0002-9157-2172), Chi Yong James	Ngu (0000-0001-5233-457X), Nan Zun	Teo (0000-0002-5475-476X)(Changi General Hospital)

**Somalia**

Abdirahman Mohamed Osman (0000-0003-0604-1375), Abdikhaliq Koshin (0009-0006-3553-1110), Ahmed Abdirahman Hussein (0009-0004-8128-7265),	Nasteho Mohamed Abdi (0009-0000-6133-4264), Mohamed Abdikarin Ismail	(0009-0004-2387-9991)(Hargeisa Group Hospital)

**Spain**

Ismael	Mora-Guzman	(0000-0003-0645-9050), Julian de Pedro (Complejo Hospitalario de Toledo); Santiago Alonso (0000-0002-0187-5242), Daniela Rodriguez, Alicia Rodriguez, Daniel Arcal (Hospital Universitario Fundación de Alcorcón); Virginia Jiménez Carneros (0000-0001-7894-2274), Guillermo	Medina Sanchez (0009-0002-5757-4497), Maria Vanesa García Marrero (0009-0006-1601-9162	), Susana Pretus Rubio	(0000-0002-7036-7815), Ainhoa Valle Rubio (0000-0001-8508-7640	), Alba Manuel Vazquez (0000-0002-3267-0274), Sara	Gortazar de las Casas (0000-0002-5342-2907)(Getafe University Hospital); María Luisa De Fuenmayor-Valera (0000-0001-9892-6298), Elena Viejo-Martinez	(0000-0003-2583-6691), Begoña Toribio-Combarro (0009-0001-3099-6094), Javier Ripollés-Melchor (0000-0002-1958-9316),	Javier Barambio-Buendía (0000-0002-5174-2800), Beatriz Fernández-Escudero (0009-0008-0984-8098), Felipe Acedo-Fernández de Pedro (0000-0002-8594-1349)(Infanta Leonor University Hospital); Manuel Losada (0000-0002-2993-497X), Enrique Calcerrada (0000-0001-6667-3476), Ana García-Nuñez (0009-0001-4537-2532), Amaya Alcojor (0009-0000-2898-955X),	Beatriz Diéguez (0000-0002-0444-9804), Alberto Vaquero (0000-0001-7751-3662), Alfredo Alonso (0000-0002-4690-1643)(Hospital Universitario del Sureste); Victor Eduardo Gonzabay Campos (0000-0003-3106-2477), Alba Torroella Vallejo	, Gloria	Padillo, Alejandro Carvi, Cesar	Ginesta Marti, Gabriel Cárdenas Rivera, Gustavo Mastroiani (Hm nou Delfo, Barcelona); Elena Sagarr Eisaa Cebolla (0000-0001-8869-3494), Eduardo de San Pío Carvajal (0000-0001-8267-1454), Cristina Palomar Puertas (0000-0002-6449-9491), Marta Cuartero Castro (0000-0003-2788-3985), Raquel Rios Blanco (0000-0002-1092-5205), José Manuel Muros Bayo	(0000-0002-4578-9493), Eneida Bra Insa	(0000-0001-8520-7497)(Hospital Universitario Infanta Cristina); Manuel Diez Alonso (0000-0002-0993-8498), Fernando Mendoza Moreno (0000-0002-1046-6344), Silvestra Barrena Blazquez (0000-0003-2715-1979), Javier Hernandez (0000-0002-2656-9620), Belen Matias (0000-0001-9209-7724),	Alberto	Vilar Tabanera (0000-0003-0890-4349), Alma Blazquez Martin (0000-0003-1119-9732)(Hospital Universitario Principe de Asturias); Mercedes Estaire-Gómez	(0000-0001-9153-9201), María Dolores Cancelas-Felgueras (0000-0002-8197-1217), Marina de la Fuente-Rodríguez	(0000-0002-0167-7692), José Miguel Valverde-Mantecón (0000-0001-6867-7710)(Severo Ochoa University Hospital); Beatriz de Andres-Asenjo (0000-0002-2503-8932), Guillermo Cabezudo (0000-0002-0153-5328), Cristina Santos-Gómez (0000-0002-2211-416X), Andrea Sánchez-Miguel (0009-0007-6182-8812), Carlos Jezieniecki (0000-0002-0742-8873), Alejandro Romero (0000-0003-4861-5365), Andrea Vazquez (0000-0003-3569-3890)(Hospital Clínico Universitario de Valladolid); Patricia Tejedor (0000-0001-8648-7697), María Sánchez-Rodríguez (0000-0002-1630-9956), Gladys Anhuaman Centeno, Julio Orallo Martínez (0009-0009-4924-2987), Luis Miguel Jiménez Gómez, Elena Hurtado, Silvia Kayser (Hospital General Universitario Gregorio Marañón); Milagros Carrasco (0000-0003-0151-2297), Isabel Jiménez, Pilar Martínez, Rebeca	Celdrán, Pedro Parra, José Manuel Muñoz, María Ramírez (Hospital General Reina Sofía); Ana Minaya-Bravo (0000-0002-7982-5499), Enrique González-Gonzalez (0000-0003-4815-9857), Mirian Marta Alba (0009-0002-5844-5566), Ana Sánchez Gollarte (0000-0001-9182-3501), Susana Prieto (0009-0005-4592-296X), Carlos Guijarro (Hospital del Henares); Raul Díaz-Pedrero (0000-0003-0412-939X), Ana Minaya-Bravo (0000-0002-7982-5499), Veronica Ongil, Ernesto Josue Ochoa, Diego Cordova (0000-0003-3834-2236), Laura Jimenez (0000-0002-0129-1159), Ricardo Alvarado (Hospital Universitario HM Rivas); David Moro-Valdezate (0000-0003-0425-8677), Vicente Pla-Martí	(0000-0001-7644-8416), Celia San Antonio, Andrea Gutiérrez-Valcárcel	(0009-0000-7364-4753), Stephanie García-Botello	(0000-0001-6921-4902	), José Martín-Arévalo (0000-0001-5337-9577), Leticia Pérez-Santiago	(0000-0002-3090-8330)(Hospital Clínico Universitario de Valencia); Pablo Calvo Espino	(0000-0002-7733-4899), Maria	Donat Garrido (0009-0005-0155-2784), Maria Concepción Olmo García (0009-0001-3237-8994), Ricardo Villamarín Tibaduiza (0000-0001-7274-7531), Javier Páramo Zunzunegui (0000-0002-4308-0501), Justyna Drewniak Jakubowska (0000-0003-2587-1534), Ana Isabel Ariza Ibarra (0009-0008-9794-3975)(Hospital Universitario de Móstoles); Héctor Aguado López (0000-0002-7825-0961), Beatriz Aguado Rodriguez (0009-0008-3143-1969), Maria Díaz Ruiz (0009-0007-3074-1079), Arelis Pérez Fernández (0009-0007-7458-9734), Francisco Javier Ruescas García	(0000-0001-7962-3005), Andrés García Marín (0000-0001-8291-9165)(Hellín Hospital); Graciela Valero-Navarro (0000-0002-7881-4763), Victoriano Soria-Aledo	(0000-0003-0159-4034), Rocio Garcia-Capitan (0009-0004-8736-1032), Fernando Garcia-Sanchez (0009-0003-9717-4428), Enrique Pellicer-Franco (0000-0003-0038-1221), Mónica	Mengual-Ballester (0000-0001-7228-7600), Jose Andres Garcia-Marin (0000-0001-6946-7914)(Morales Meseguer University Hospital)

**Sri Lanka**

Selvaratnam Srishankar (0000-0002-1989-7343)(Teaching Hospital Anuradhapura); Pramodh Chandrasinghe (0000-0002-3485-961X), Aniradha Neligama, Nayana	Abeysinghe (0000-0001-7551-4442), Bhagya Gunetilleke (0000-0002-7019-8222)(University of Kelaniya, North Colombo Teaching Hospital); Dakshitha	Wickramasinghe (0000-0002-2324-5022), Dileesha Wickramasinghe, Rasika Nilangani, Vihara Dasanayaka (National Hospital of Sri Lanka)

**Sudan**

Adam AbdAla (0009-0002-8595-8500), Baraa Ali (0009-0005-5875-1290), Ibrahim Ahmed (0009-0008-6322-0242), Mai	Salah (0009-0006-2556-9094), Iman Abdalla (0009-0002-3023-5620), Sara Yousif (0009-0006-2256-3893), Samar Ali (0009-0002-2879-743X)(Ed Dueim Teaching Hospital); Abuzaid Mohamedali (0009-0007-9794-7012), Yasen Kamal (0009-0002-5798-6432), Renad Fagery (0009-0007-3739-1067), Ehssan Mohamedelnazir (0009-0005-8904-2796), Mawada Abdulhadi (0009-0004-0501-7175), Mohammed Osman Ahmed Osman (0009-0001-4389-1937), Mohaned Mustafa	(0009-0004-5625-8243)(Dongola Specialized Hospital); Ibrahim Nagmeldin Hassan (0009-0003-3331-8138), Khalid Alrwa (0009-0005-8224-5806), Omsalama Ibrahim (0009-0007-7407-4968), Mohamed Ibrahim (0009-0005-7370-1849)(Ibrahim Said Hospital); Abdulrahman Abouh (0009-0007-5665-5486), Mohammedbabalrahma Koko, Thuriya, Modawi, Mai Bushra, Abdalla Adam, Ahmed Idris (El Obeid Teaching Hospital); Ahmed Mohamed Ibrahim Mohamed (0000-0001-5063-2637), Mihad	Abdalla Mahmoud (0009-0009-4601-8779), Khalid Omer (0009-0002-7310-7370), Yassin Moner (0009-0005-0878-3606), Bahaaldeen Osman Hassan Abker (0009-0007-7658-5745), Mohamed Abdalbagi Ahmed Ibrahim (0009-0005-3212-8548), Mohamed Musa Yassin (0000-0002-0696-2978), Hassan Salih Hussein Eisa (0000-0003-2760-5571)(Gadarif Teaching Hospital – team 1); Mohamed Musa Yassin (0000-0002-0696-2978), Mohamed Almahdi (0000-0003-1097-0070), Aljarrah Mohammed (0009-0002-4504-8545), Mohamad Alhadi, Faihaa Siddig, Islam Daboka Fono (Gadarif Teaching Hospital – team 2); Alshima Siddig Mohamed Khalifa, Kamal Ali Mohammed Salih, Zeinab Siddig Ahmed Bakhiet, Anfal Yousif Ahmd Ezat, Mohamad Abdelbagi Sidahmed Ali, Samah Elhussein Mohamed Ahmed, Aziza Ibraheem Suliman Abdalla (Gadarif teaching hospital – team 3); ElSagad Eltayeb Gady (0000-0002-2120-9933), Mohanned Kamal Mahmoud Elmuzamil (0009-0008-4060-6134), Hossam Aldeen Alhareth (0009-0004-1279-3586), Ahmed Alsisi (0009-0004-9181-5869), Nehal Mohamed (0009-0009-6685-1784)(Atbara); Majdi Abuidris Abderrahim Dafa Allah (0009-0000-8332-8279), Ibrahim Mutwakil Gamal Ahmed (0000-0001-8847-8567), Mohaned Awad Ahmed Daoud (0009-0001-6144-9536), Waleed Abdalla Altayeb (0009-0007-6686-3297), Mustafa Khalifa Mohamed Kheir Hamed (0009-0006-9515-9306), Gamal Mutwakil Gamal Ahmed (0000-0001-8192-8000)(Al-Hassahissa Teaching Hospital); Mazin Alzebeer (0009-0008-3115-7402), Hebatallah Fadhl (0009-0004-4498-7510), Muhammed Ali (0009-0004-6962-9947), Magdi Essam (0009-0004-3879-1503), Asmaa Altegani (0009-0006-3872-3783), Ibrahim Gareeb (0009-0001-9329-9405), Hadeel Omara (0009-0005-9571-7171)(Atbara Teaching Hospital); Sami Galal Eldin (0000-0001-7143-0372), Yusra Badri Elhuda Abdelaziz (0009-0006-3322-8353), Abd Elrahim (0009-0006-4651-2644), Razan Khalid (0009-0007-6169-0438), Abdelrahim Abdelrazig Ahmed Ibrahim (0000-0003-0292-0786), Sara Osman (0009-0002-8020-5761), Musab Hafiz (0009-0003-9618-1028)(Al Mobarak Hospital); Asaad Ahmed (0000-0003-0846-286X), Yousef Hamed (0009-0005-7038-4374), Mohamed Hadal (0009-0001-7806-2645), Sanabil Mohamed (0009-0008-9601-1869), Ahmed Hassan (0009-0004-9212-7988), Yasmin Ammar (0009-0001-6494-1630)(Sea Corporation Hospital Portsudan)

**Sweden**

Najia Azhar (0000-0001-6709-4441), Carl-Viktor Hellberg, Jenny Lindgren, Silvia Marchesi (0000-0003-4791-4507), Pamela Buchwald (0000-0001-6407-5552), Valentinus Valdimarsson (0000-0003-0129-674X), Pernilla Hansdotter Andersson (0000-0003-4887-0243)(Skane University Hospital Malmö)

**Switzerland**

Sofia Xenaki (0000-0002-1394-4276), Antonio	Nocito, Alex Ochsner, Andreas Keerl, Andrea Wirsching (BAG Bundesamt für Gesundheit – Schweiz)

**Syria**

Hala Almobi Alsaid Mushaweh	(0000-0002-6654-5973), Youssef Zeeb, Modar Al Ali, Oubada Elmolla	, Mjd Al Khateeb (Harasta National Hospital); Samir Kanaan (0009-0007-6229-5480), Hussein Mohammad (0009-0004-0835-0555), Rawia Dooba (0009-0001-0716-277X), Mohammad Ibrahim (0009-0005-7787-3904), Hamed	Tarboosh (0009-0004-4722-6346), Noor Haidar	(0009-0009-9383-690X), Ammar Kanaan (0009-0000-6501-4911)(Tishreen University Hospital); Altayeb Alhaj Zain (0009-0002-7753-9912),	Ahmad	Alusef (0000-0002-4792-3609), Nawwar Reslan (0009-0004-6559-6009), Saleem Alabdullah (0009-0005-1378-7791), Hadi Alabdullah (0009-0003-1424-5308)(Damascus Hospital – team 1); Alaa Aldirani (0000-0002-6710-1135), Mohammad Alawir (0009-0002-0928-9608), Ehsan Albakri (0009-0008-5728-4514), Mouhamad Al-Khatib (0009-0001-6961-9851), Yulian Mhana (0009-0000-5346-9230)( (Damascus Hospital – team 2); Mohannad Nasani (0000-0001-5093-8986), Jamal Houari (0000-0001-8425-5091), Diaa Mohammad Hamad (0009-0005-3621-3045),	Mohammad Tammam Saffour	(0009-0005-2107-7773), Nazeeh Alaktaa (0000-0001-8281-4064), Mohamed	Alaktaa (0000-0002-6926-8393), Merei	Aweni (0009-0001-3599-2447)(Al-Shahbaa Private Hospital)

**Taiwan**

Jaw-Yaun Wang (0000-0002-7705-2621), I-Chieh Lee (0000-0002-7378-426X), PengJen Huang (0000-0002-4495-0900), Pojung Chen (Kaohsiung Medical University Hospital)

**Tunisia**

Anis Hasnaoui	(0000-0002-7147-2529), Racem Trigui	(0000-0001-8565-1483), Sherif Ben Salem, Mohamed Amine Sassi (Menzel Bourguiba Hospital); Chaima	Yakoubi (0000-0002-5174-2624), Mohamed Ali Mseddi, Raja Younsi, Chokri Elomri, Rami Guizani, Rakia Siala (La Rabta Hospital – team 1); Amine Sebai (0000-0002-8077-5775), Ahmed Ben Mahmoud (0000-0003-4158-6661), Mariem Ben Brahim (0009-0007-7312-0977), Souhaib Atri (0000-0002-6252-6345), Montasser Kacem (0000-0002-7819-0110), Anis Haddad (0000-0002-2617-9753), Houcine Maghrebi (0000-0002-0769-9286)(La Rabta Hospital – team 2); Abdelkader Mizouni,	Marwa Azri, Asma Ounaies, Habiba Ben Hamada, Mehdi Ben Latifa, Amal	Bouazzi, Sami Lagha (Sahloul Hospital)

**Turkey**

Yunus Emre Altuntaş (0000-0002-1908-0085),	Nail Can Adigüzel (0009-0003-5680-5051), Esra Kunduz (0009-0008-2981-5912), Mücahit Okçu (0009-0009-4087-4494), Omar Alomari (0000-0003-3651-4129), Muhammed Edib Mokresh (0000-0002-0239-5814), Gizem Duran (0009-0008-2981-5912)(Kartal Dr. Lutfi Kirdar Training and Research Hospital); Alp Ömer Cantürk (0000-0003-3641-7628), Fatih Altıntoprak (0000-0002-3939-8293), Meryem	Çelik (0000-0003-4932-5263), Ali Fuat Erdem (0000-0001-6994-397X), Erhan	Eröz (0000-0003-1044-1013), Kayhan Özdemir (0000-0002-8041-198X)(Sakarya University Training and Research Hospital); Banu Yigit (0000-0001-7606-9352	), Serhat Meric	(0000-0001-7660-5688), Guler	Erol (0009-0003-7440-8316), Merve Gokce (0009-0002-7461-2117), Ahmet Sencer Ergin (0000-0001-6369-6146), Alparslan Saylar (0000-0001-6187-2556), Nihat Bugdayci (0000-0002-6881-3222)(Bagcilar Research And Training Hospital); Elif Colak (0000-0002-1893-6427), Mine Gizem Bidil (0009-0005-5681-143X), Sadan Somuncuoglu Ozbilgin (0000-0001-6543-2348), Hale Kefeli Celik (0000-0002-0850-4524), Ahmet Burak	Ciftci (0000-0002-1814-4008),	Kursat Yemez (0000-0002-8875-1049), Huseyin Eraslan (0000-0002-3193-221X) Serdar Şenol (0000-0002-6084-2491) (Samsun Training and Research Hospital); Korhan Tuncer (0000-0001-7458-828X), Gizem Kılınç Tuncer (0000-0002-6987-3198), Özge Yolcu (0009-0005-5724-9450),	Hakan Aygün (0000-0002-6152-0857),	Cem Tuğmen (0000-0002-2668-5197), Eyüp Kebapçı (0000-0001-8900-2325), Serdar Ünlü (0000-0002-1147-0628)(Bakircay University Cigli Training and Research Hospital); Yunushan Furkan Aydoğdu (0000-0002-2418-2393), Ömer Kubat (0000-0002-6825-8353), Gizem	Kılıç Aydoğdu (0009-0000-0346-3578), Burak Ersun (0000-0001-6805-314X)(Gölbaşı Şehit Ahmet Özsoy State Hospital); Semra Demirli Atici (0000-0002-8287-067X), Aras Emre Canda (0000-0002-8257-5881), Özgür Bolat (0000-0003-4411-1696),	Mustafa Cem Terzi (0000-0003-2523-5140)(Acibadem Kent Hospital); Afag Aghayeva (0000-0002-5317-8545), Bilgi Baca (0000-0003-1704-2533), Ebru Kirbiyik	(0000-0002-2945-5100), Hasan Cem Guneyli (0000-0002-2450-9552), Mustafa Ege Seker (0000-0001-7664-5786), Bengi Agca (0000-0002-0922-724X), Metincan Erkaya (0000-0002-4707-6059)(Acibadem Altunizade Hospital); Hüseyin Kerem Tolan (0000-0002-0845-8820), Enes Sertkaya (0000-0003-1626-8303), Sevgi Öztürk (0000-0002-4424-6577),	Mustafa Deniz Dinkçi (0000-0001-8213-4341), Mert Gedik (0000-0001-6483-8927), Emre Furkan Kırkan (0000-0001-9534-3396), Muhammed Oğuz çelikkaya (0000-0002-1894-7206); Osman Bozbiyik (0000-0002-1827-2720), Bahadir Emre Baki (0000-0003-3389-1332), Demet Yurtsever, Alev Atalay, Cemil Caliskan	(0000-0002-2652-3390), Erhan Akgün	(0000-0003-3608-7040), Mustafa Korkut	(0000-0002-8077-0536)(Ege University Hospital); Server Sezgin Uludağ (0000-0002-0563-3769), Ergin Erginöz (0000-0002-8349-3298), Dilara Kılınç, Şafak Emre Erbabacan (0000-0001-9314-9178), Seda Erdil, Serkan Akkuş, Mahmut Alper Güldağ (0000-0003-4306-8999)(Cerrahpasa Medical Faculty Istanbul University); Ali Cihat Yildirim (0000-0001-5379-2804), Muhammed Alperen Taş (0000-0002-4060-121X), Hülya Zorbas, Serkan Telli (0000-0001-8045-5205), Sezgin Zeren (0000-0002-9342-1706)(Kutahya Health Sciences University Evliya Çelebi Education and Research Hospital); Cihangir Akyol (0000-0002-3941-5268), Mehmet Ali Koc (0000-0002-4605-048X), Selma Dincel, Suheyla Karadag Erkoc (0000-0001-5086-5916), Siyar Ersoz (0000-0003-1824-8453), Kaan Sunter (0000-0003-3927-1361), Selcuk Gulec (0000-0002-3779-5914)(Ankara University Medical School); Fatma Ayca Gultekin (0000-0002-4148-5871), Yigit Ozaydın (0000-0003-2096-9778), Ozkan Guler (0000-0001-6804-2950), Keziban Bollucuoglu (0000-0002-7111-8685), Ufuk Tali	(0009-0007-0812-1711)(Zonguldak Bulent Ecevit University School of Medicine Research and Training Hospital); Veysel Barış Turhan (0000-0001-5093-4993), Ertuğrul Gazi Alkurt (0000-0002-3044-5428), Furkan Uğur (0009-0001-2382-3080), Murtaza Salih Kepez (0009-0004-6513-8227), Semin Turhan (0000-0002-1014-1179), Bahadır Kartal (0000-0003-1647-9979), Mehmet Yalvaç (0000-0002-4342-4597), Murat Kendirci (0000-0002-6594-3777)(Hitit University Erol Olçok Training and Research Hospital); Fevzi Cengiz (0000-0002-1614-5568), Örgün Güneş	(0000-0002-0576-6086), Meltem Ulus (0000-0001-9779-8597), Murat Aksun (0000-0002-8308-3045)(Izmir Katip Celebi Uni); Rıfat Peksöz (0000-0003-4658-5254), Muhammet Yıldırım (0000-0001-7480-6769), Nurbanu Uzunget (0009-0009-3461-5373), Ali Ahıskalıoğlu (0000-0002-8467-8171), Sabri Selçuk Atamanalp (0000-0003-2561-6461), Yavuz Albayrak (0000-0002-2535-4329)(Ataturk University School of Medicine, Research and Training Hospital); Can Sahin (0000-0003-3946-821X); Umut Emreol (0000-0002-5399-7196), Esra Gunduz	(0000-0001-7610-7603), Semih Aydemir (0000-0002-1087-3070), Furkan Akin (0000-0003-3613-0598)(Yildirim Beyazit University Yenimahalle Training and Research Hospital); Tahsin Colak	(0000-0002-7253-5608), Cumhur Ozcan (0000-0002-6453-025X), Ruhnaz Okyay (0009-0001-0949-2182), Levent Ozdemir (0000-0002-7780-3202), Enver Reyhan (0000-0001-8210-5479),	Hilmi Bozkurt (0000-0003-0389-0523), Mustafa Berkesoglu (0000-0002-5850-5592)(Mersin University Medical Faculty); Sami Acar (0000-0003-4096-3963), Cagil Karaevli (0000-0002-4280-7430), Merve Karanfil (0009-0008-4938-8217), Ahmet Gultekin (0000-0003-4570-8339)(Namik Kemal University Hospital); Ufuk Memiş (0000-0003-3393-3301), Mücahit Taş (0009-0006-0870-563X), Seçkin Karakus (0000-0001-8088-7462), Alparslan Koç (0000-0002-5965-9761)(Erzincan University Hospital); Ökkeş Sezai Leventoğlu (0000-0003-0680-0589), Denizcan Bozkurt (0000-0002-4152-2388), Şerife Naz Bozkurt (0000-0002-5889-8373), Metin Alkan	(0000-0002-0043-8091), Ramazan Kozan (0000-0002-3835-8759), Aydın Yavuz (0000-0003-0091-7997), Ahmet Çağrı Büyükkasap (0000-0002-9141-4289)(Gazi University Medical Faculty Hospital); Tayfun Bisgin (0000-0001-7040-4228), Sevgi Deniz Köse (0009-0004-4082-5565), Sevda Özkardesler (0000-0002-9660-2094), Selman Sokmen (0000-0003-2628-6898), Berke Manoğlu (0000-0003-4755-2200), Caner Bektaş (0000-0002-8907-7738)(Dokuz Eylul Univ. Hospital); Omer Faruk Ozkan (0000-0002-6644-2413); Merve Karadag	(0000-0001-9101-4536), Ozlem Akoglu (0009-0005-8211-3168), Suheyla Abitagaoglu (0000-0003-4937-0080), Haluk Kerim Karakullukcu (0000-0002-1180-8297), Hanife Seyda Ulgur Genc (0000-0003-0896-3285), Hilmi Bora Isceviren (0000-0001-8132-9999)(Sultan 2. Abdulhamid Han Training and Research Hospital, University of Health Sciences); Ahmet Necati	Sanli (0000-0002-1483-8176), Yusuf Iskender Tandogan (0000-0002-9345-5412), Erdem Durum	(0000-0002-8766-9035), Deniz Altiok Tandogan (0000-0002-3723-835X), Alparslan Ertenlice (0000-0002-8227-5328)(Abdulkadir Yuksel State Hospital)

**Ukraine**

Stepan	Grytsenko (0000-0002-6422-093X), Yurii Nedoshytko,	Oksana Shymchuk, Rostyslav Yuryk (Ternopil Regional Oncological Clinic); Andrii Beznosenko, Veronika Rozhkova, Olena Rybachuk, Ivan Lisnyy, Anatoli Shudrak, Vitaliy Zvirych (National Cancer Institute, Kyiv); Sergii Podpriatov (0000-0001-5942-6311), Olexandr	Ivanko (0000-0003-0036-4675), Nataliia Kudelia, Olena Levytska (0000-0002-6283-7397), Andrii Martynenko (0009-0008-1387-0191),	Sergey Trepet, Oleksandr Hryb (0009-0009-2272-8338)(Kyiv City Clinical Hospital № 1); Ievgen Ilin (0000-0001-9183-6652), Alina	Semeniuk (0009-0008-7914-8302), Oksana Grusha (0009-0004-1303-2228), Genadij Farina (0009-0004-5450-333X), Victor Zhylchuk	(0009-0005-4094-2494), Oleksandr Tkach (0009-0000-2782-1181)(Rivne Regional Antitumor Center)

**United Kingdom**

Ramy Shaalan	(0000-0003-0010-1066), Claire Carden (0000-0002-6588-1764), Lauren Harper, Joanna	Lynch (0000-0002-1425-8462), Tarek Khalil (0000-0002-3621-2764), Morven Stewart, Neville	Ekpete	(0000-0002-1184-9283)(Ninewells Hospital); Jayan Jayasinghe (0000-0003-3929-2699), Joussi Ramzi, Pamela Buyson, Charlotte Dempsey, Mohamed Adnan Thaha, Annamaria Minicozzi, Prashanth Chowdary (Royal London Hospital); Prem Thambi, Mohamed Mansour, Rebecca Metcalfe, Ahmed Elfoly, Peter Bennett, Parth Gada (James Cook University Hospital); Frank McDermott (0000-0002-4891-1191),Antonios Gklavas, Emily Fisher, Tom Clark, Rob Bethune, Polly	Estridge, Priya Nandoskar (Royal Devon and Exeter Hospital); Ahmed	Nada (0000-0001-7129-303X), Lahreb Aktar (0000-0001-6361-9108), Sophie Bennett (0009-0007-5160-1200), Christopher Harmer (University Hospital North Durham); Gary Nicholson (0000-0002-9239-2034), Mishal Shahid (0000-0003-4727-4052), Finlay Young (0009-0000-4783-6444), John-Patrick Byars (0009-0008-3356-1003), Aidan Doye (Queen Elizabeth University Hospital, Glasgow); Mewhish Ansar, Claire Kilbride, Tom Kennedy, Rifat Mohamed Oluwaseun, Prashant Nair (Wirral University Teaching Hospital); Panna Pateel (0000-0002-9344-2654), Siddhartha Handa (0000-0001-9389-416X), Karen	Robinson (0009-0000-0420-9934), Wael Abdelrhman (0009-0002-7131-7614	), Ralph Obure	(0000-0002-1150-0831), Chrysanthi Karagianni (0000-0002-0561-4699)(Furness General Hospital)

**Yemen**

Faris Mohammed Abdulwali Alhajami (0000-0003-2526-5358), Nezar Mohammed Mohammed Mahyoub (0009-0007-8529-2258), Sultan Ahmed Ibrahim Al-Hamidi (0009-0007-5462-5697), Hemier Mannsor Nasr	Al-Shaif (0009-0001-6325-103X), Hussein Abdullah Ahmed Doan (0009-0002-0878-405X), Gamal Abdulrahman Khaled Al-Qubati (0009-0006-2153-0176), Mohammed Abdullah Aqlan Al-Hershi (0009-0000-7135-981X)(Al-Gumhouri Teaching Hospital)
