# Supplementary material for: Impact of safe-anastomosis digital training on 30-day anastomotic leak after right colectomy: international prospective cohort study (EAGLE-2)
Source: Br J Surg. 2026 Sep 11;113(9):znag107. doi: 10.1093/bjs/znag107 (PMC13564221; doi:10.1093/bjs/znag107)
Supplement: Supplementary file 1 [file znag107_supplementary_data.zip › Supplementary-Material.docx]

**Impact of safe-anastomosis digital training on 30-day anastomotic leak after right colectomy: an international prospective cohort study (EAGLE-2)**

ESCP EAGLE Safe Anastomosis Collaborative and NIHR Global Health Research Unit in Surgery^1^

^1^European Society of Coloproctology and the University of Birmingham

**Corresponding author.** Elizabeth Li, Applied Health Research, University of Birmingham, UK, B15 2TT. [Y.e.li@bham.ac.uk](mailto:Y.e.li@bham.ac.uk) **ORCID ID** 0000-0001-5961-2894; **Twitter** @yanelizabethli

**Supplementary Materials - Index**

| **Supplementary Methods** |  |
| --- | --- |
| Supplementary Table S1: STROBE checklist for observational studies | *page 2* |
| **Supplementary Figures and Tables** |  |
| Supplementary Table S2. Hospital characteristics  Supplementary Table S3: Additional operative factors  Supplementary Table S4**.** Anastomotic leaks and intraabdominal or pelvic collections in elective and emergency surgery  Supplementary Table S5: Adjusted analysis for anastomotic leak  Supplementary Table S6. Anastomotic leak rates in hospitals recruited to EAGLE-1, EAGLE-2 or both | *page 4*  *page 5*  *page 6*  *page 7*  *page 7* |
| Supplementary Table S7: Patient demographics by if the in-theatre checklist was completed or not  Supplementary Table S8: Operative factors by if the in-theatre checklist was completed or not  Supplementary Table S9: Clinical and operative outcomes by if the anastomotic leak risk was calculated pre-operatively  Supplementary figure S1: Map of number of patients entered the EAGLE-2 study  Supplementary figure S2: Alluvial plot of patients’ urgency of presentation, intraoperative contamination level and anastomotic leaks  Supplementary Figure S3: Different grades of anastomotic leak and pelvic collection in patients where the operating surgeon did and did not complete the training | *page 8*  *page 9*  *page 10*  *page 11*  *page 12*  *page 13* |

**Supplementary Methods**

Supplementary Table S1: STROBE checklist for observational studies

|  | Item No | Recommendation | Page number |
| --- | --- | --- | --- |
| **Title and abstract** | 1 | (*a*) Indicate the study’s design with a commonly used term in the title or the abstract | 1 |
|  |  | (*b*) Provide in the abstract an informative and balanced summary of what was done and what was found | 2 |
| Introduction | | |  |
| Background/rationale | 2 | Explain the scientific background and rationale for the investigation being reported | 3 |
| Objectives | 3 | State specific objectives, including any prespecified hypotheses | 3 |
| Methods | | |  |
| Study design | 4 | Present key elements of study design early in the paper | 5 |
| Setting | 5 | Describe the setting, locations, and relevant dates, including periods of recruitment, exposure, follow-up, and data collection | 5 |
| Participants | 6 | (*a*) Give the eligibility criteria, and the sources and methods of selection of participants. Describe methods of follow-up | 5 |
|  |  | (*b*) For matched studies, give matching criteria and number of exposed and unexposed | 5 |
| Variables | 7 | Clearly define all outcomes, exposures, predictors, potential confounders, and effect modifiers. Give diagnostic criteria, if applicable | 6 |
| Data sources/ measurement | 8* | For each variable of interest, give sources of data and details of methods of assessment (measurement). Describe comparability of assessment methods if there is more than one group | 6 |
| Bias | 9 | Describe any efforts to address potential sources of bias | 6 |
| Study size | 10 | Explain how the study size was arrived at | 6 |
| Quantitative variables | 11 | Explain how quantitative variables were handled in the analyses. If applicable, describe which groupings were chosen and why | 6 |
| Statistical methods | 12 | (*a*) Describe all statistical methods, including those used to control for confounding | 6 |
|  |  | (*b*) Describe any methods used to examine subgroups and interactions | N/A |
|  |  | (*c*) Explain how missing data were addressed | 6 |
|  |  | (*d*) If applicable, explain how loss to follow-up was addressed | N/A |
|  |  | (*e*) Describe any sensitivity analyses | N/A |
| Results | | |  |
| Participants | 13* | (a) Report numbers of individuals at each stage of study—eg numbers potentially eligible, examined for eligibility, confirmed eligible, included in the study, completing follow-up, and analysed | 8, 21 |
|  |  | (b) Give reasons for non-participation at each stage | 21 |
|  |  | (c) Consider use of a flow diagram | 21 |
| Descriptive data | 14* | (a) Give characteristics of study participants (eg demographic, clinical, social) and information on exposures and potential confounders | 8 |
|  |  | (b) Indicate number of participants with missing data for each variable of interest | 21 |
|  |  | (c) Summarise follow-up time (eg, average and total amount) | 8 |
| Outcome data | 15* | Report numbers of outcome events or summary measures over time | 8 |
| Main results | 16 | (*a*) Give unadjusted estimates and, if applicable, confounder-adjusted estimates and their precision (eg, 95% confidence interval). Make clear which confounders were adjusted for and why they were included | 8 |
|  |  | (*b*) Report category boundaries when continuous variables were categorized | 8 |
|  |  | (*c*) If relevant, consider translating estimates of relative risk into absolute risk for a meaningful time period | N/A |
| Other analyses | 17 | Report other analyses done—eg analyses of subgroups and interactions, and sensitivity analyses | 8 |
| Discussion | | |  |
| Key results | 18 | Summarise key results with reference to study objectives | 10 |
| Limitations | 19 | Discuss limitations of the study, taking into account sources of potential bias or imprecision. Discuss both direction and magnitude of any potential bias | 11 |
| Interpretation | 20 | Give a cautious overall interpretation of results considering objectives, limitations, multiplicity of analyses, results from similar studies, and other relevant evidence | 11 |
| Generalisability | 21 | Discuss the generalisability (external validity) of the study results | 11 |
| Other information | | |  |
| Funding | 22 | Give the source of funding and the role of the funders for the present study and, if applicable, for the original study on which the present article is based | 1 |

**Supplementary Figures and Tables**

Supplementary Table S2. Hospital characteristics

| **Hospital factors** | **Levels** | **Number of Hospitals (%)** | **Operating surgeon did NOT complete training (n=633)** | **Operating surgeon completed training (n=2242)** |
| --- | --- | --- | --- | --- |
| **Region** | Africa | 56 (16.9) | 84 (13.3) | 250 (11.2) |
|  | Asia | 52 (15.7) | 61 (9.6) | 414 (18.5) |
|  | Europe | 204 (61.5) | 464 (73.3) | 1447 (64.6) |
|  | South America | 17 (5.1) | 20 (3.2) | 111 (4.9) |
|  | Oceania | 3 (0.9) | 4 (0.6) | 19 (0.9) |
| **Country income** | Low | 19 (5.7) | 13 (2.1) | 65 (2.9) |
|  | Middle | 128 (38.6) | 168 (26.5) | 869 (38.8) |
|  | High | 185 (55.7) | 452 (71.4) | 1307 (58.3) |

Supplementary Table S3: Additional operative factors

| **Factors** | **Levels** | **Operating surgeon did NOT complete training (n=633)** | **Operating surgeon completed training (n=2242)** | **Total (n=2875)** |
| --- | --- | --- | --- | --- |
| If yes, configuration | Side to side | 476 (84.1) | 1671 (81.2) | 2147 (81.9) |
|  | Side to end | 37 (6.5) | 139 (6.8) | 176 (6.7) |
|  | End to end | 53 (9.4) | 247 (12.0) | 300 (11.4) |
| If yes, defunctioning loop | No | 546 (96.5) | 1944 (94.5) | 2490 (94.9) |
|  | Yes | 20 (3.5) | 113 (5.5) | 133 (5.1) |
| If yes, anastomosis tested | No | 417 (73.7) | 1364 (66.3) | 1781 (67.9) |
|  | Yes – air leak tested | 92 (16.3) | 417 (20.3) | 509 (19.4) |
|  | Yes – probed with forceps | 57 (10.1) | 276 (13.4) | 333 (12.7) |
| If yes, anastomosis require revision | No | 133 (89.3) | 631 (91.1) | 764 (90.7) |
|  | Yes | 16 (10.7) | 62 (8.9) | 78 (9.3) |
| Intra-operative complications | Blood loss >1L | 12 (1.9) | 37 (1.7) | 49 (1.7) |
|  | Operating time >4h | 17 (2.7) | 87 (3.9) | 104 (3.6) |
|  | Solid organ or ureteric injury | 7 (1.1) | 12 (0.5) | 19 (0.7) |
|  | Vascular injury | 2 (0.3) | 19 (0.8) | 21 (0.7) |
|  | Blood transfusion | 14 (2.2) | 83 (3.7) | 97 (3.4) |
|  | Hemodynamic instability | 13 (2.1) | 53 (2.4) | 66 (2.3) |
|  | Vasopressor requirement | 8 (1.3) | 47 (2.1) | 55 (1.9) |
| Duration of surgery | 1-60 | 7 (1.1) | 36 (1.6) | 43 (1.5) |
|  | 61-119 | 100 (15.8) | 443 (19.8) | 543 (18.9) |
|  | 120-179 | 266 (42.0) | 962 (42.9) | 1228 (42.7) |
|  | 180-239 | 180 (28.4) | 514 (22.9) | 694 (24.2) |
|  | >=240 | 80 (12.6) | 285 (12.7) | 365 (12.7) |
|  | Missing | 0 | 2 | 2 |

Supplementary Table S4**.** Anastomotic leaks and intraabdominal or pelvic collections in elective and emergency surgery

|  | **Overall** | | **Elective** | | **Emergency** | |
| --- | --- | --- | --- | --- | --- | --- |
|  | **Operating surgeon did NOT complete training** | **Operating surgeon completed training** | Operating surgeon did **NOT** complete training | Operating surgeon completed training | Operating surgeon did **NOT** complete training | Operating surgeon completed training |
| **Intrabdominal or pelvic collection** | 82 (13.0%) | 157 (7.0%) | 32 (7.9%) | 85 (5.2%) | 50 (21.8%) | 72 (12.1%) |
| **Anastomotic leak** | 62 (9.8%) | 117 (5.2%) | 29 (7.2%) | 74 (4.5%) | 33 (14.4%) | 43 (7.2%) |
| **Total** | 102 (16.1%) | 204 (9.2%) | 46 (11.4%) | 117 (7.1%) | 56 (24.5%) | 87 (14.7%) |

Supplementary Table S5: Adjusted analysis for anastomotic leak

|  |  | **No leak** | **Leak** | **OR (95% CI)** | **P-value** |
| --- | --- | --- | --- | --- | --- |
| Surgeon completed training | No | 521 (83.6) | 102 (16.4) | Reference |  |
|  | Yes | 2003 (90.8) | 204 (9.2) | 0.56 (0.41-0.78) | <0.001 |
| Urgency | Elective | 1854 (91.9) | 163 (8.1) | Reference |  |
|  | Emergency | 669 (82.4) | 143 (17.6) | 1.58 (1.15-2.17) | 0.005 |
| ASA grade | Grade 1-2 | 1517 (90.8) | 154 (9.2) | Reference |  |
|  | Grade 3-5 | 1006 (86.9) | 152 (13.1) | 1.44 (1.09-1.80) | 0.009 |
| Contamination | Clean-contaminated | 2249 (91.8) | 202 (8.2) | Reference |  |
|  | Contaminated | 192 (73.3) | 70 (26.7) | 3.04 (2.07-4.48) | <0.001 |
|  | Dirty | 83 (70.9) | 34 (29.1) | 3.25 (1.90-5.54) | <0.001 |

Supplementary Table S6. Anastomotic leak rates in hospitals recruited to EAGLE-1, EAGLE-2 or both

| **Study** | **Group** | **n** | **Anastomotic leak rate** | **95% CI** |
| --- | --- | --- | --- | --- |
| **Eagle 2 only** | **Control** | 396 | 15.2% | 11.7% - 18.7% |
| **Eagle 2 only** | **Intervention** | 1414 | 9.7% | 8.2% - 11.2% |
| **Eagle 1 & Eagle 2** | **Control** | 802 | 11.5% | 9.3% - 13.7% |
| **Eagle 1 & Eagle 2** | **Intervention** | 1273 | 8.4% | 6.9% - 9.9% |

CI = confidence interval

Supplementary Table S7: Patient demographics by if the in-theatre checklist was completed or not

| **Factors** | **Levels** | **Anastomosis leak risk calculated** | | **Checklist completed** | |
| --- | --- | --- | --- | --- | --- |
|  |  | **Yes** | **No** | **Yes** | **No** |
| Age (years) | Median (IQR) | 66.0 (53.0 to 75.0) | 67.0 (54.0 to 76.0) | 67.0 (53.0 to 76.0) | 66.0 (54.0 to 76.0) |
| Sex | Female | 389 (50.5) | 917 (43.6) | 345 (50.2) | 961 (43.9) |
|  | Male | 382 (49.5) | 1188 (56.4) | 342 (49.8) | 1228 (56.1) |
| Previous abdominal surgery | No | 455 (59.0) | 1306 (62.1) | 398 (57.9) | 1363 (62.3) |
|  | Yes | 316 (41.0) | 797 (37.9) | 289 (42.1) | 824 (37.7) |
| BMI > 30 | No | 620 (80.4) | 1674 (79.6) | 555 (80.8) | 1739 (79.6) |
|  | Yes | 151 (19.6) | 428 (20.4) | 132 (19.2) | 447 (20.4) |
| Known diabetes | No | 619 (80.3) | 1588 (75.6) | 557 (81.1) | 1650 (75.5) |
|  | Yes | 152 (19.7) | 513 (24.4) | 130 (18.9) | 535 (24.5) |
| History of IHD or CVA | No | 599 (77.7) | 1658 (78.9) | 533 (77.6) | 1724 (78.9) |
|  | Yes | 172 (22.3) | 444 (21.1) | 154 (22.4) | 462 (21.1) |
| Oral anti-coagulants | No | 648 (84.0) | 1798 (85.6) | 579 (84.3) | 1867 (85.4) |
|  | Yes | 123 (16.0) | 303 (14.4) | 108 (15.7) | 318 (14.6) |
| Pre-operative total protein level (g/dL) | ≤4 | 64 (8.4) | 172 (8.2) | 57 (8.3) | 179 (8.2) |
|  | 4.5 | 47 (6.1) | 168 (8.0) | 41 (6.0) | 174 (8.0) |
|  | 5 | 47 (6.1) | 124 (5.9) | 46 (6.7) | 125 (5.7) |
|  | 5.5 | 47 (6.1) | 197 (9.4) | 35 (5.1) | 209 (9.6) |
|  | 6 | 79 (10.3) | 276 (13.2) | 66 (9.6) | 289 (13.3) |
|  | 6.5 | 97 (12.7) | 332 (15.8) | 90 (13.2) | 339 (15.6) |
|  | 7 | 89 (11.6) | 365 (17.4) | 94 (13.7) | 360 (16.5) |
|  | 7.5 | 36 (4.7) | 188 (9.0) | 40 (5.8) | 184 (8.4) |
|  | ≥8 | 21 (2.7) | 55 (2.6) | 18 (2.6) | 58 (2.7) |
|  | Unknown | 239 (31.2) | 219 (10.4) | 197 (28.8) | 261 (12.0) |
| Pre-operative haemoglobin (g/dL) | < 60 | 7 (0.9) | 6 (0.3) | 2 (0.3) | 11 (0.5) |
|  | 60-69 | 6 (0.8) | 21 (1.0) | 6 (0.9) | 21 (1.0) |
|  | 70-79 | 23 (3.0) | 42 (2.0) | 19 (2.8) | 46 (2.1) |
|  | 80-89 | 74 (9.6) | 161 (7.7) | 61 (8.9) | 174 (8.0) |
|  | 90-99 | 91 (11.8) | 283 (13.5) | 76 (11.1) | 298 (13.6) |
|  | 100-109 | 120 (15.6) | 337 (16.0) | 107 (15.6) | 350 (16.0) |
|  | 110-119 | 117 (15.2) | 391 (18.6) | 115 (16.8) | 393 (18.0) |
|  | 120-129 | 124 (16.1) | 320 (15.2) | 114 (16.6) | 330 (15.1) |
|  | 130-139 | 103 (13.4) | 244 (11.6) | 79 (11.5) | 268 (12.3) |
|  | ≥140 | 95 (12.3) | 286 (13.6) | 99 (14.4) | 282 (12.9) |
|  | Unknown | 11 (1.4) | 10 (0.5) | 8 (1.2) | 13 (0.6) |

BMI = kg/m^2.^ IHD – ischemic heart disease, CVA- cerebral vascular accident

Supplementary Table S8: Operative factors by if the in-theatre checklist was completed

| **Factors** | **Levels** | **Anastomosis leak risk calculated** | | **Checklist completed** | |
| --- | --- | --- | --- | --- | --- |
|  |  | **Yes** | **No** | **Yes** | **No** |
| Timing of surgery | Elective (planned) | 481 (62.4) | 1568 (74.6) | 411 (59.8) | 1638 (75.0) |
|  | Expedited  (within two weeks of decision) | 86 (11.2) | 249 (11.9) | 88 (12.8) | 247 (11.3) |
|  | Emergency (unplanned) | 204 (26.5) | 284 (13.5) | 188 (27.4) | 300 (13.7) |
| Indication for surgery | Malignancy | 552 (71.7) | 1696 (80.7) | 494 (72.0) | 1754 (80.2) |
|  | Inflammatory bowel disease | 69 (9.0) | 163 (7.8) | 61 (8.9) | 171 (7.8) |
|  | Other | 149 (19.4) | 243 (11.6) | 131 (19.1) | 261 (11.9) |
| Bowel preparation | None | 484 (62.8) | 988 (47.0) | 409 (59.5) | 1063 (48.6) |
|  | Mechanical bowel preparation only | 177 (23.0) | 505 (24.0) | 155 (22.6) | 527 (24.1) |
|  | Mechanical bowel preparation with oral antibiotics | 110 (14.3) | 609 (29.0) | 123 (17.9) | 596 (27.3) |
| American Society of Anaesthesiologists (ASA) grade | Grade I | 106 (13.7) | 314 (14.9) | 84 (12.2) | 336 (15.4) |
|  | Grade II | 335 (43.5) | 937 (44.6) | 281 (41.0) | 991 (45.3) |
|  | Grade III | 284 (36.8) | 738 (35.1) | 269 (39.2) | 753 (34.4) |
|  | Grade IV | 42 (5.4) | 108 (5.1) | 45 (6.6) | 105 (4.8) |
|  | Grade V | 4 (0.5) | 6 (0.3) | 7 (1.0) | 3 (0.1) |
| Primary operating surgeon | Consultant colorectal surgeon | 355 (46.0) | 1136 (54.0) | 308 (44.8) | 1183 (54.1) |
|  | Trainee colorectal surgeon | 56 (7.3) | 194 (9.2) | 43 (6.3) | 207 (9.5) |
|  | Consultant general surgeon | 277 (35.9) | 604 (28.7) | 252 (36.7) | 629 (28.8) |
|  | Trainee general surgeon | 83 (10.8) | 168 (8.0) | 84 (12.2) | 167 (7.6) |
| Operative approach | Open | 405 (52.5) | 883 (42.0) | 344 (50.1) | 944 (43.2) |
|  | Laparoscopic (completed) | 285 (37.0) | 899 (42.8) | 266 (38.7) | 918 (42.0) |
|  | Laparoscopic (converted to open) | 56 (7.3) | 151 (7.2) | 49 (7.1) | 158 (7.2) |
|  | Robotic (completed) | 24 (3.1) | 160 (7.6) | 27 (3.9) | 157 (7.2) |
|  | Robotic (converted to open) | 1 (0.1) | 9 (0.4) | 1 (0.1) | 9 (0.4) |
| Operative field contamination | Clean |  |  |  |  |
|  | Clean-contaminated | 635 (82.4) | 1853 (88.2) | 560 (81.5) | 1928 (88.2) |
|  | Contaminated | 86 (11.2) | 182 (8.7) | 77 (11.2) | 191 (8.7) |
|  | Dirty | 50 (6.5) | 67 (3.2) | 50 (7.3) | 67 (3.1) |
| Anastomosis formed | Stapled | 452 (58.6) | 1443 (68.6) | 408 (59.4) | 1487 (68.0) |
|  | Handsewn | 233 (30.2) | 495 (23.5) | 196 (28.5) | 532 (24.3) |
|  | No anastomosis | 86 (11.2) | 164 (7.8) | 83 (12.1) | 167 (7.6) |
| If yes, configuration | Side to side | 548 (80.0) | 1599 (82.5) | 490 (81.1) | 1657 (82.1) |
|  | Side to end | 45 (6.6) | 131 (6.8) | 41 (6.8) | 135 (6.7) |
|  | End to end | 92 (13.4) | 208 (10.7) | 73 (12.1) | 227 (11.2) |
| If yes, defunctioning loop | No | 653 (95.3) | 1837 (94.8) | 584 (96.7) | 1906 (94.4) |
|  | Yes | 32 (4.7) | 101 (5.2) | 20 (3.3) | 113 (5.6) |
| If yes, anastomosis tested | No | 526 (76.8) | 1255 (64.8) | 450 (74.5) | 1331 (65.9) |
|  | Yes – air leak tested | 96 (14.0) | 413 (21.3) | 87 (14.4) | 422 (20.9) |
|  | Yes – probed with forceps | 63 (9.2) | 270 (13.9) | 67 (11.1) | 266 (13.2) |
| If yes, anastomosis require revision | No | 142 (89.3) | 622 (91.1) | 134 (87.0) | 630 (91.6) |
|  | Yes | 17 (10.7) | 61 (8.9) | 20 (13.0) | 58 (8.4) |
| Intra-operative complications | Blood loss >1L | 14 (1.8) | 35 (1.7) | 8 (1.2) | 41 (1.9) |
|  | Operating time >4h | 23 (3.0) | 81 (3.8) | 22 (3.2) | 82 (3.7) |
|  | Solid organ or ureteric injury | 9 (1.2) | 10 (0.5) | 12 (1.7) | 7 (0.3) |
|  | Vascular injury | 7 (0.9) | 14 (0.7) | 4 (0.6) | 17 (0.8) |
|  | Blood transfusion | 27 (3.5) | 70 (3.3) | 22 (3.2) | 75 (3.4) |
|  | Hemodynamic instability | 23 (3.0) | 43 (2.0) | 22 (3.2) | 44 (2.0) |
|  | Vasopressor requirement | 20 (2.6) | 35 (1.7) | 16 (2.3) | 39 (1.8) |
| Duration of surgery | 1-60 | 16 (2.1) | 27 (1.3) | 9 (1.3) | 34 (1.6) |
|  | 61-119 | 171 (22.2) | 372 (17.7) | 142 (20.7) | 401 (18.3) |
|  | 120-179 | 301 (39.0) | 927 (44.1) | 263 (38.3) | 965 (44.1) |
|  | 180-239 | 188 (24.4) | 506 (24.1) | 176 (25.6) | 518 (23.7) |
|  | >=240 | 95 (12.3) | 270 (12.8) | 97 (14.1) | 268 (12.3) |

Supplementary Table S9: Clinical and operative outcomes by if the anastomotic leak risk was calculated pre-operatively

| **Factors** | **Levels** | **Anastomosis leak risk calculated** | | **Checklist completed** | |
| --- | --- | --- | --- | --- | --- |
|  |  | **Yes** | **No** | **Yes** | **No** |
| Post-operative critical care admission | None | 1467 (70.7) | 505 (66.6) | 1539 (71.3) | 433 (64.1) |
|  | Planned from theatre | 543 (26.2) | 201 (26.5) | 560 (26.0) | 184 (27.3) |
|  | Unplanned from theatre | 43 (2.1) | 35 (4.6) | 38 (1.8) | 40 (5.9) |
|  | Unplanned from ward | 21 (1.0) | 17 (2.2) | 20 (0.9) | 18 (2.7) |
| Total length of hospital stay | Days (up to 30 post-operative days)  Median +IQR | 7.0 (5.0 to 10.0) | 7.0 (5.0 to 11.0) | 7.0 (5.0 to 10.0) | 7.0 (5.0 to 11.5) |
| Anastomotic leak | No | 1966 (94.9) | 685 (90.4) | 2048 (95.0) | 603 (89.3) |
|  | Yes | 106 (5.1) | 73 (9.6) | 107 (5.0) | 72 (10.7) |
| Grade of the anastomotic leak | None | 7 (6.5) | 2 (2.7) | 8 (7.3) | 1 (1.4) |
|  | Grade A – requiring no further intervention | 23 (21.5) | 14 (18.9) | 24 (22.0) | 13 (18.1) |
|  | Grade B – requiring radiological reintervention | 20 (18.7) | 13 (17.6) | 22 (20.2) | 11 (15.3) |
|  | Grade C – requiring surgical reintervention | 57 (53.3) | 45 (60.8) | 55 (50.5) | 47 (65.3) |
| If yes: How was the leak diagnosed? | Clinical diagnosis only | 13 (12.5) | 19 (26.0) | 14 (13.3) | 18 (25.0) |
|  | Ultrasound imaging | 9 (8.7) | 3 (4.1) | 9 (8.6) | 3 (4.2) |
|  | CT imaging | 81 (77.9) | 48 (65.8) | 81 (77.1) | 48 (66.7) |
|  | MR imagining | 0 (0.0) | 0 (0.0) | 0 (0.0) | 0 (0.0) |
|  | Intra-operative diagnosis | 1 (1.0) | 3 (4.1) | 1 (1.0) | 3 (4.2) |
| Intra-abdominal or pelvic collection | No | 1923 (92.9) | 666 (87.9) | 2007 (93.2) | 582 (86.4) |
|  | Yes | 147 (7.1) | 92 (12.1) | 147 (6.8) | 92 (13.6) |
| Grade of intra-abdominal or pelvic collection | None | 3 (2.0) | 2 (2.2) | 5 (3.4) | 0 (0.0) |
|  | Grade A – requiring no further intervention | 60 (40.8) | 31 (33.7) | 60 (40.8) | 31 (33.7) |
|  | Grade B – requiring radiological reintervention | 38 (25.9) | 17 (18.5) | 36 (24.5) | 19 (20.7) |
|  | Grade C – requiring surgical reintervention | 46 (31.3) | 42 (45.7) | 46 (31.3) | 42 (45.7) |
| If yes: How was the collection diagnosed? | Clinical diagnosis only | 10 (6.9) | 15 (16.3) | 11 (7.6) | 14 (15.2) |
|  | Ultrasound imaging | 28 (19.4) | 15 (16.3) | 28 (19.4) | 15 (16.3) |
|  | CT imaging | 101 (70.1) | 60 (65.2) | 100 (69.4) | 61 (66.3) |
|  | MR imagining | 0 (0.0) | 0 (0.0) | 0 (0.0) | 0 (0.0) |
|  | Intra-operative diagnosis | 5 (3.5) | 2 (2.2) | 5 (3.5) | 2 (2.2) |
| Anastomotic leak or Intra-abdominal/pelvic collection | No | 1885 (91.0) | 639 (84.3) | 1968 (91.3) | 556 (82.4) |
|  | Yes | 187 (9.0) | 119 (15.7) | 187 (8.7) | 119 (17.6) |
|  |  |  |  |  |  |
| Re-operation within 30 days | No | 1939 (93.9) | 674 (88.9) | 2013 (93.6) | 600 (89.0) |
|  | Yes | 127 (6.1) | 84 (11.1) | 137 (6.4) | 74 (11.0) |
| Re-admission within 30 days | No | 1940 (93.9) | 683 (90.1) | 2013 (93.6) | 610 (90.5) |
|  | Yes | 126 (6.1) | 75 (9.9) | 137 (6.4) | 64 (9.5) |
| Mortality within 30 days | No | 2009 (97.2) | 720 (95.0) | 2094 (97.4) | 635 (94.2) |
|  | Yes | 57 (2.8) | 38 (5.0) | 56 (2.6) | 39 (5.8) |

Supplementary figure S1: Map of number of patients entered the EAGLE-2 study

Supplementary figure S2: Alluvial plot of patients’ urgency of presentation, intraoperative contamination level and anastomotic leaks

Supplementary Figure S3: Different grades of anastomotic leak and pelvic collection in patients where the operating surgeon did and did not complete the training
